# Immunomodulatory Nanosystems

**DOI:** 10.1002/advs.201900101

**Published:** 2019-06-21

**Authors:** Xiangru Feng, Weiguo Xu, Zhongmin Li, Wantong Song, Jianxun Ding, Xuesi Chen

**Affiliations:** ^1^ Key Laboratory of Polymer Ecomaterials Changchun Institute of Applied Chemistry Chinese Academy of Sciences Changchun 130022 P. R. China; ^2^ University of Science and Technology of China Hefei 230026 P. R. China; ^3^ Department of Gastrointestinal Colorectal and Anal Surgery China–Japan Union Hospital of Jilin University Changchun 130033 P. R. China

**Keywords:** disease treatment, immunostimulation, immunosuppression, immunotherapy, nanotechnology

## Abstract

Immunotherapy has emerged as an effective strategy for the prevention and treatment of a variety of diseases, including cancer, infectious diseases, inflammatory diseases, and autoimmune diseases. Immunomodulatory nanosystems can readily improve the therapeutic effects and simultaneously overcome many obstacles facing the treatment method, such as inadequate immune stimulation, off‐target side effects, and bioactivity loss of immune agents during circulation. In recent years, researchers have continuously developed nanomaterials with new structures, properties, and functions. This Review provides the most recent advances of nanotechnology for immunostimulation and immunosuppression. In cancer immunotherapy, nanosystems play an essential role in immune cell activation and tumor microenvironment modulation, as well as combination with other antitumor approaches. In infectious diseases, many encouraging outcomes from using nanomaterial vaccines against viral and bacterial infections have been reported. In addition, nanoparticles also potentiate the effects of immunosuppressive immune cells for the treatment of inflammatory and autoimmune diseases. Finally, the challenges and prospects of applying nanotechnology to modulate immunotherapy are discussed.

## 1 Introduction

Our immune system is able to protect us from a variety of illnesses based on a process termed “immune surveillance.”^1^ Theoretically, viruses, bacteria, and cancer cells can be rapidly identified as alien antigens and eliminated by immune cells. However, the disturbing reality is that successful pathogens have developed a range of effective mechanisms to evade immune clearance by inhibiting phagocytosis, blocking antigen presentation, or directly killing immune cells.^2^ Worse still, cancer cells can alter the tumor microenvironment (TME) into a highly immunosuppressive state by recruiting immunosuppressive immune cells and by expressing a series of inhibitory cytokines, enzymes, and checkpoint molecules, thus facilitating tumor immune evasion.^3^ These barriers undoubtedly hinder the efficiency and intensity of the immune responses. On the contrary, aberrant activation of immune cells can arouse uncontrolled inflammation and cause inflammatory diseases, autoimmune diseases, or allergic diseases.^4^ Abnormal inflammation can also lead to transplant rejection and hinder tissue and organ regeneration.^5^ Therapy interventions are necessary to maintain the homeostasis and function of the immune system.

**Scheme 1:**
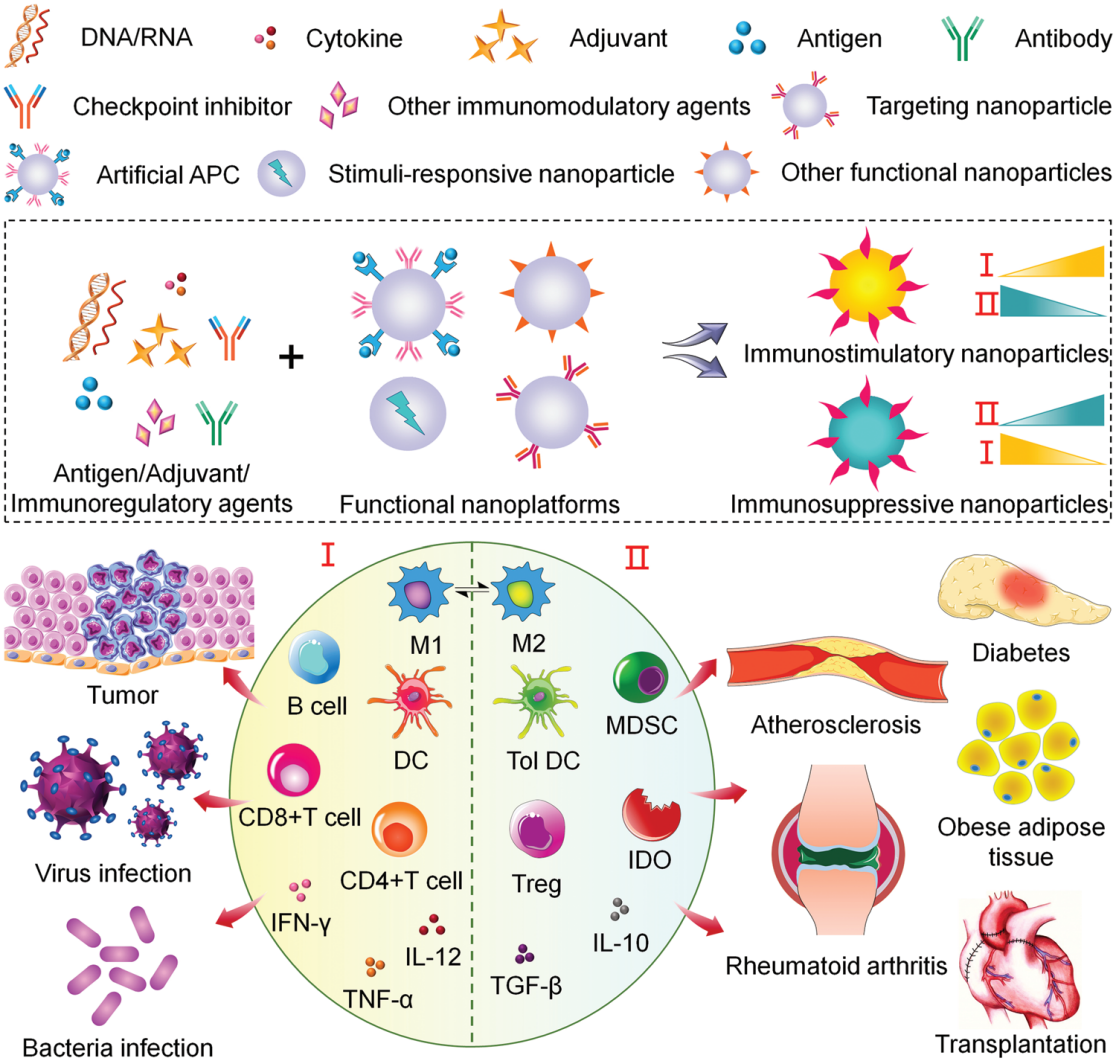
Engineering of immunostimulatory nanoparticles and immunosuppressive nanoparticles based on functional nanoplatforms, and their applications in treatment of various diseases by regulating immune‐related cells, cytokines, and enzymes.

The concept of treating a disease by activating or suppressing immune system is referred to as immunotherapy. In the treatment of cancer and infectious diseases, immunostimulatory therapy should be used for the activation of immune response to detect and eliminate non‐self‐antigens, and to establish memory effects for these diseases. On the contrary, for overactive immune response in diseases like atherosclerosis, rheumatoid arthritis (RA), diabetes, obesity, and transplantation, immunosuppressive therapy is needed to downregulate immune reaction and generate certain immune tolerance (**Scheme** 1). Our immune environment can be regulated by a variety of immune cells, cytokines, and enzymes, which can be investigated to properly control and prevent immune‐related disorders or illnesses. Many immunotherapeutic methods have achieved impressive outcomes in treating various diseases,^6^ but performances of immunoregulatory agents can be negatively affected by poor solubility, high immune‐mediated toxicity, and loss of bioactivity after long circulation.^7^

Encouragingly, nanotechnology is in a position to solve the existing problems, and thereby achieves the desired therapeutic effect. Studies have shown that the nanoplatforms manifest numerous advantageous properties, including 1) codelivery of antigens and adjuvants to the same antigen presenting cells (APCs) or intracellular compartments;^8^ 2) prolonged half‐lives of bioactive cargo molecules by avoiding degradation by enzymes during blood circulation;^9^ 3) increased accumulation in tumor tissues through size‐dependent enhanced permeability and retention (EPR) effect;^10^, ^11^, ^12^ 4) surface modification for targeting toward specific tissues or cells;^13^, ^14^ 5) stimuli‐sensitive behavior for safe trafficking and intelligent drug release;^15^, ^16^, ^17^ 6) higher tolerant dosages of drugs due to less accumulation at off‐target organs and tissues;^18^ 7) surface coupling of both antigens and costimulatory molecules to engineer artificial APCs (aAPCs) for potent T cell activation;^19^ 8) diversified drug delivery routes, such as intranasal administration or subcutaneous delivery by microneedle patch;^8^, ^20^, ^21^ 9) intrinsic immunomodulatory functions of engineered nanoparticles.^22^, ^23^

To date, researchers have synthesized nanoparticles with diverse structures and biological functions for drug delivery. Some of the most frequently explored nanosystems are polymer nanoparticles,^24^, ^25^ liposomes,^26^, ^27^ micelles,^28^, ^29^, ^30^ nanogels,^14^, ^31^, ^32^ gold nanoparticles (Au NPs),^33^, ^34^ and carbon nanomaterials.^35^ These nanoplatforms have shown phenomenal capabilities in assisting immunostimulatory or immunosuppressive regulation by targeted delivery and stimuli‐responsive controlled release of antigens, adjuvants, and immunoregulatory agents (Scheme 1). The aAPCs and nanoparticles with other functions are also engineered for diverse purposes. One strategy to improve the localization of encapsulated cargoes in target tissues or cells is chemical modification of nanoparticles with targeting moieties. For example, nanomaterials decorated with DEC‐205 antibody (Ab), CD40 Ab, CD11c Ab, or mannose can be preferentially internalized by dendritic cells (DCs) through receptor‐mediated endocytosis.^36^, ^37^, ^38^ Similarly, folate, lectins, and CD44 are utilized for recognition by corresponding receptors overexpressed on macrophages.^39^, ^40^, ^41^ Surface coupling of CD3 Ab or tLyp1 peptide has shown increased uptake by T cells and regulatory T cells (Tregs), respectively.^42^, ^43^ Besides, nanoplatforms consisting of dextran or dextran sulfate have intrinsic targeting property toward macrophages.^44^, ^45^ More recently, through a method termed “albumin hitchhiking,” nanoparticles with albumin‐binding domains are capable of draining to lymph node (LN).^46^ Additionally, researchers have paid more attention to the specific functionalization of nanoparticles in the treatment of a wide range of diseases, where nanoparticles perform as a main constituent rather than a delivery vehicle.

Specially, in tumor immunotherapy, stimuli‐responsive nanomaterials are engineered in order to maintain structural integrity in serum and facilitate specific payload release in TME.^47^, ^48^, ^49^, ^50^ The internal and external stimuli‐responsive strategies (pH,^16^, ^51^, ^52^, ^53^ reduction,^15^, ^54^, ^55^ enzymes,^27^, ^56^ light,^57^, ^58^, ^59^ heat,^13^ and reactive oxygen species (ROS)^60^) are involved in nanoparticle design and have achieved improved antitumor effects. In addition, other antitumor molecules and agents can be coloaded into these nanoplatforms for combinational therapy with synergistic effect.

Collectively, the structure and functional characteristics of nanoparticles have a great modulatory impact on immunotherapeutic efficiency. Herein, we highlight the recent development of nanosystems in improving immune stimulation and immunosuppression. Different modalities of nanomaterial‐incorporated strategies in enhancing tumor immunotherapy and combining immunotherapy with other antitumor therapies are specifically discussed. We also outline the nanoparticle‐based immunomodulation toward virus and bacterial infection, autoimmune diseases, and other inflammatory disorders. The future prospects and challenges in this field are also predicted.

## 2 Nanoparticles for Immunostimulation

### 2.1 Immunomodulatory Nanoparticles for Cancer Treatment

In the past few decades, immunotherapy has become a fast‐growing approach to treat cancer.^121^, ^122^ Unlike chemotherapy, radiotherapy, and surgery, it aims to activate immune cells to detect and eradicate tumor cells. In this way, the side effects toward normal organs and tissues can be significantly reduced. Moreover, immunotherapeutic strategy also provides long‐term protection against tumor relapse by inducing immunological memory.^123^, ^124^, ^125^ Lately, immune checkpoint blockade therapy targeting cytotoxic T‐lymphocyte antigen 4 (CTLA‐4), programmed cell death 1 (PD‐1), or programmed cell death ligand 1 (PD‐L1) has raised wide attention in relieving the negative regulation over T cells.^126^ Adoptive cell transfer (ACT) especially chimeric antigen receptor (CAR)‐T cell therapy using ex vivo expanded and genetically engineered T cells for antigen‐specific tumor therapy, was also approved recently by U.S. Food and Drug Administration (FDA) for B‐cell and non‐Hodgkin lymphoma therapy.^127^ Based on the fascinating advancement, cancer immunotherapy efficacy can be further improved with the assistance of nanotechnology. However, immunotherapeutic methods are limited in the treatment of solid tumors owing to highly immunosuppressive TME as well as abnormal extracellular matrix. More seriously, the “off‐target” effects of the immune‐modulatory agents can cause damage to normal tissues and cells.

Nanotechnology improves the therapeutic efficacy of cancer immunotherapy mainly through three aspects: 1) protection of antigens and adjuvants, especially in the case of nucleic acid; 2) efficient delivery to APCs and initiation of potent tumor antigen‐specific immune response; 3) reprogramming of TME to resume immune surveillance. Until now, a large number of nanoparticle‐based delivery systems aiming at the modulation of immune cells have been developed for cancer treatment,^128^, ^129^ and some of them have come into various clinical trial stages (**Table** 1).^130^, ^131^ These clinical researches confirmed their great therapeutic potential as antitumor agents. For example, in a phase II clinical trial (NCT00157209), non‐small‐cell lung cancer (NSCLC) patients vaccinated with tecemotide (L‐BLP25) containing immunoadjuvant monophosphoryl lipid A and a synthetic mucin 1 (MUC1) lipopeptide, showed an enhanced three‐year survival of 49% compared with 27% in patients that received the best supportive care only.^67^ To achieve precise and controlled drug delivery, smart nanoparticles with more complex structures and specific drug release properties are also being produced according to the hallmarks of TME, such as weak acidic pH (6.5–6.8), high level of glutathione and hydrogen peroxide (H_2_O_2_), and disorder of proteinases production, such as matrix metalloproteinase‐2 (MMP‐2).^56^, ^126^, ^132^, ^133^, ^134^ In this section, we overview the recent developments in the use of engineered nanoparticles to enhance cancer immunotherapy. As a booming research area, the combination therapy with chemotherapy, phototherapy, or radiotherapy is also discussed. Nanoparticle‐based immunomodulatory systems for cancer immunotherapy were listed in **Table** 2.

**Table 1:** Overview of clinical trials using nanoparticle‐based immunotherapy against cancer and infectious diseases

Drug	Basic material	Mode of administration	Company	Indication	Status	Responsible party	ClinicalTrials.gov identifier	Combinatorial treatments	Country	Patient enrollment	Duration	Reference
Tecemotide (L‐BLP25)	Liposome	s.c.	Oncothyreon Canada Inc.	Prostate cancer	Phase II	EMD Serono	NCT01496131	Radiation therapy, goserelin, CPA	U.S.	28	2011–2016	61
				Rectal cancer	Phase II	Merck KGaA, Darmstadt, Germany	NCT01507103	CPA, chemoradiotherapy	Netherlands	124	2012–2014	–
				Slowly progressive multiple myeloma	Phase II	Merck KGaA, Darmstadt, Germany	NCT01094548	Single or multiple low dose CPA	Germany	34	2008–2012	62
				NSCLC	Phase III	EMD Serono	NCT02049151	CPA	U.S.	35	2014–2015	63
				Stage III unresectable NSCLC	Phase I/II	Merck KGaA, Darmstadt, Germany	NCT00960115	Single low dose CPA	Japan	178	2008–2015	64
				Stage III, unresectable NSCLC	Phase III	Merck KGaA, Darmstadt, Germany	NCT01015443	Single low dose CPA	China, Korea, Singapore	285	2009–2015	65
				Lung cancer	Phase II	ECOG‐ACRIN Cancer Research Group	NCT00828009	Bevacizumab, carboplatin, CPA, paclitaxel, radiotherapy	U.S.	70	2010–2019	–
				NSCLC	Phase II	Merck KGaA, Darmstadt, Germany	NCT00157196	Single low dose CPA	–	22	2005–2012	66
				Stage IIIB or stage IV NSCLC	Phase II	Merck KGaA, Darmstadt, Germany	NCT00157209	Single low dose CPA	Germany	171	2000–2012	67
				Unresectable stage III NSCLC	Phase III	EMD Serono	NCT00409188	Single low dose CPA, chemoradiotherapy	U.S., Argentina, Australia, Austria, etc. 23 countries	1513	2007–2015	68, 69, 70
				Advanced breast cancer	Phase III	EMD Serono	NCT00925548	CPA	U.S., Australia, Austria, Belgium, etc. 12 countries	16	2009–2010	71
Lipo‐MERIT	Liposome	i.v.	BioNTech	Advanced melanoma	Phase I	Biontech RNA Pharmaceuticals GmbH	NCT02410733	–	Germany	115	2015–2019	72
DPX‐0907	Liposome	s.c.	IMV Inc.	HLA‐A2 positive advanced stage ovarian, breast, prostate cancer	Phase I	IMV Inc.	NCT01095848	–	U.S.	23	2010–2011	73
DPX‐ Survivac	Liposome	s.c.	IMV Inc.	Recurrent ovarian cancer	Phase I/II	IMV Inc.	NCT02785250	Epacadostat, low dose oral CPA	U.S.	85	2016–2020	–
				Advanced stage ovarian, fallopian, peritoneal cancer	Phase I	IMV Inc.	NCT01416038	Low dose oral CPA	U.S. and Canada	19	2011–2013	74
				Advanced ovarian, primary peritoneal, fallopian tube cancer	Phase II	University Health Network, Toronto	NCT03029403	Pembrolizumab, low dose oral CPA	Canada	42	2018–2024	–
				Advanced stage ovarian, fallopian, peritoneal cancer	Phase I	IMV Inc.	NCT03332576	Low dose oral CPA	U.S. and Canada	37	2013–2017	–
				Ovarian, hepatocellular, bladder cancer, NSCLC	Phase II	IMV Inc.	NCT03836352	Pembrolizumab, low dose oral CPA	U.S. and Canada	232	2018–2022	–
				Persistent or recurrent/refractory DLBCL	Phase II	Sunnybrook Health Sciences Centre	NCT03349450	Pembrolizumab, low dose CPA	Canada	25	2018–2021	–
				Recurrent survivin‐expressing DLBCL	Phase II	IMV Inc.	NCT02323230	Low dose oral CPA	Canada	24	2015–2018	75
CHP‐NY‐ESO‐1	Cholesteryl pullulan	s.c.	ImmunoFrontier, Inc.	HER2 and/or NY‐ESO‐1‐expressing esophageal, lung, stomach, breast, ovarian cancer	Phase I	–	NCT00291473	CHP‐HER2, OK‐432	Japan	9	2005–2008	76
				Esophageal cancer	Phase I	ImmunoFrontier, Inc.	NCT01003808	–	Japan	25	2009–2012	77
				NY‐ESO‐1‐expressing breast, prostate cancer	Phase I	–	NCT00106158	–	Japan	9	2004–2006	78
CYT‐6091	Colloidal Au NP	i.v.	CytImmune Sciences, Inc.	Unspecified adult solid tumor	Phase I	–	NCT00356980	–	U.S.	60	2006–2009	79
				Adrenocortical, breast, colorectal, gastrointestinal, kidney, ovarian, pancreatic, liver cancer, melanoma, sarcoma	Early phase I	–	NCT00436410	Conventional surgery	U.S.	108	2006–2009	–
Oncoquest‐L	Proteoliposome	s.c.	XEME Biopharma Inc.	Stage III or IV, asymptomatic, nonbulky follicular lymphoma	Phase II	XEME Biopharma Inc.	NCT02194751	–	U.S.	30	2020–2023	–
AS15	Liposome	i.m.	GlaxoSmithKline	Metastatic breast cancer	Phase I/II	Duke University	NCT00952692	dHER2, lapatinib	U.S.	12	2009–2012	80
				Localized breast cancer at high‐risk of relapse	Phase I	Institut Pasteur	NCT02364492	MAG‐Tn3	France	30	2017–2021	–
				Muscle‐invasive bladder cancer after cystectomy	Phase II	European Association of Urology Research Foundation	NCT01435356	recMAGE‐A3	Czechia, France, Germany, Italy, etc. 10 countries	83	2011–2017	81
				HER2‐overexpressing high‐risk breast cancer	Phase I	GlaxoSmithKline	NCT00058526	dHER2	U.S., France, Australia, Belgium, Italy	61	2003–2006	82
				NSCLC after tumor removal	Phase II	GlaxoSmithKline	NCT01853878	recPRAME	U.S., Estonia, France, Germany, etc. 9 countries	137	2013–2016	83
				Melanoma	Phase I	GlaxoSmithKline	NCT01149343	recPRAME	Czech Republic, France, Germany, Italy, etc. 6 countries	107	2010–2016	84
				Multiple myeloma	Phase I	Ludwig Institute for Cancer Research	NCT01380145	recMAGE‐A3	U.S.	14	2011–2014	85
				Melanoma after tumor removal	Phase III	GlaxoSmithKline	NCT00796445	recMAGE‐A3	U.S., Argentina, Australia, Austria, etc. 32 countries	1351	2008–2016	86
				Stage IIB–IV resected melanoma	Early phase I	Human Immune Therapy Center, University of Virginia	NCT01425749	recMAGE‐A3	U.S.	25	2011–2015	87
				Melanoma	Phase II	M.D. Anderson Cancer Center	NCT01266603	recMAGE‐A3, high‐dose IL‐2	U.S.	30	2011–2018	88
				Melanoma	Phase II	H. Lee Moffitt Cancer Center and Research Institute	NCT01437605	recMAGE‐A3, with or without Poly IC:LC	U.S.	14	2019–2020	–
Lipovaxin MM	Liposome	i.v.	Lipotek Pty Ltd.	Melanoma	Phase I	Lipotek Pty Ltd.	NCT01052142	–	Australia	12	2009–2012	89
ISCOMATRIX	Liposome	i.m.	CSL Biotherapies Inc.	High‐risk, resected melanoma	Phase II	–	NCT00199901	NY‐ESO‐1	Australila, New zealand, U.K.	111	2005–2011	90
				Mesolthelioma, esophageal, lung, thoracic cancer, sarcomas, thymoma	Phase I	NCI	NCT01258868	Celebrex	U.S.	44	2010–2016	–
				Sarcoma, melanoma, epithelial malignancies, pleural malignancy	Phase I	NCI	NCT01341496	Epigenetically modified autologous tumor, celecoxib, CPA	U.S.	41	2011–2016	–
				Thoracic sarcomas, thorasic cancers, sarcoma, melanoma	Phase I/II	NCI	NCT02054104	H1299 cell lysate, celecoxib, CPA	U.S.	21	2014–2020	–
				Measurable stage III or IV melanoma	Phase II	Ludwig Institute for Cancer Research	NCT00518206	NY‐ESO‐1, CPA	Australia	46	2003–2010	91
JVRS‐100	Liposome	s.c.	Juvaris BioTherapeutics, Inc.	Relapsed or refractory leukemia	Phase I	Milton S. Hershey Medical Center	NCT00860522	Plasmid DNA	U.S.	23	2009–2017	92, 93
Melan‐A VLPs	VLPs	Intra nodal injection	Cytos Biotechnology	Stage III/IV malignant melanoma	Phase II	Head Clinical Development, Cytos Biotechnology AG	NCT00651703	Montanide, and imiquimod	Switzerland	21	2008–2010	94
				Stage IV and recurrent melanoma	Phase I	Centre Hospitalier Universitaire Vaudois	NCT00324623	IMP321, fludarabine phosphate, and CPA	Switzerland	8	2005–2011	95
JVRS‐100	Liposome	i.m.	Juvaris BioTherapeutics, Inc.	Influenza	Phase II	Maggie Sisti, Juvaris	NCT00936468	Fluzone vaccine	–	472	2009–2010	96
				Influenza	Phase I	Maggie Sisti, Juvaris BioTherapeutics, Inc.	NCT00662272	Fluzone vaccine	U.S.	128	2008–2009	97
Epaxal	Virosome	s.c.	Crucell Company, Berna Biotech Ltd.	Hepatitis A	Phase I	Crucell Holland BV	NCT01307436	DTPaHibIPV, OPV, and MMR vaccines	Israel	327	2011–2013	–
				Response to Hepatitis A vaccine in patients with IBD	Phase IV	Asan Medical Center	NCT01341808	–	Republic of Korea	493	2011–2013	98
				Hepatitis A	Phase II	Crucell Holland BV	NCT01405677	–	Belgium	308	2004–2012	99
				Hepatitis A	Phase IV	Helsinki University Central Hospital	NCT01926860	Prevenar13	Finland, Sweden	300	2013–2015	–
				Response to Hepatitis A vaccine in RA patients	Phase II	Sormland County Council, Sweden	NCT01360970	–	Finland, Sweden	68	2009–2011	100
				Response to Hepatitis A vaccine in RA patients	–	University of Zurich	NCT01947465	With or without tetanus vaccination	Switzerland	645	2013–2016	101
Inflexal V	Virosome	i.m.	Crucell Company, Berna Biotech Ltd.	Influenza	Phase III	Crucell Holland BV	NCT01229397	–	Italy	205	2010	102
				Influenza	Phase III	Crucell Holland BV	NCT01310400	–	China	1356	2009–2010	–
				Influenza	Phase III	Crucell Holland BV	NCT01893177	–	Switzerland	110	2013	–
				Influenza	Phase III	Crucell Holland BV	NCT01631110	–	Switzerland	110	2012	–
				Influenza	Phase IV	Crucell Holland BV	NCT01306305	–	Switzerland	110	2010	–
				Influenza	Phase IV	Crucell Holland BV	NCT01303510	–	Switzerland	111	2008	–
				Influenza	Phase IV	Crucell Holland BV	NCT01306253	–	Switzerland	114	2009	–
				Influenza	Phase IV	Crucell Holland BV	NCT01457027	–	Italy	52	2011–2012	–
				Influenza	Phase III	Crucell Holland BV	NCT01229371	–	Switzerland	440	2010	–
				Falciparum malaria	Phase I	Swiss Tropical & Public Health Institute	NCT00513669	–	Tanzania	50	2008–2009	103
				Influenza	Phase I	Crucell Holland BV	NCT01617239	–	Belgium	84	2012–2013	–
				Influenza	Phase I/II	Baxter Healthcare Corporation	NCT00976469	–	Austria and Germany	400	2009–2011	104
				Influenza	Phase III	Crucell Holland BV	NCT01412281	–	Switzerland	440	2011	–
				Influenza	Phase I	Crucell Holland BV	NCT02148328	–	Belgium	240	2014	–
CAF01	Liposome	i.m.	Statens Serum Institut	Tuberculosis	Phase I	Statens Serum Institut	NCT00922363	Ag85B‐ESAT‐6	Netherlands	38	2009–2011	105
				Chlamydia trachomatis	Phase I	Statens Serum Institut	NCT02787109	CTH522 chlamydia antigen	U.K.	35	2016–2017	106
				HIV	Phase I	Statens Serum Institut	NCT01141205	AFO‐18	Guinea‐Bissau	18	2009–2012	107
				HIV	Phase I	Hvidovre University Hospital	NCT01009762	AFO‐18	Denmark	11	2009–2012	108
Rpg120/HIV‐1SF2 (BIOCINE)	Liposome	i.d.	NIAID	HIV	Phase I	NIAID	NCT00001042	Liposome‐encapsulated monophosphoryl lipid A	U.S.	112	1996	109
ISCOMATRIX	Liposome	i.m.	CSL Biotherapies Inc.	Influenza	Phase I	Merck Sharp & Dohme Corp.	NCT00851266	BIPCV and aluminum	–	187	2006–2009	110
RTS,S/AS01	Liposome	i.m.	GlaxoSmithKline	Malaria	Phase III	London School of Hygiene and Tropical Medicine	NCT03143218	–	Burkina Faso, Mali	5920	2017–2020	–
				Plasmodium falciparum malaria	Phase I/II	University of Oxford	NCT01883609	ChAd63 ME‐TRAP, MVA ME‐TRAP	U.K.	48	2013–2014	111
				Malaria	Phase II	GlaxoSmithKline	NCT00380393	–	Kenya, Tanzania	894	2007–2008	112, 113, 114
				Malaria	Phase I/II	GlaxoSmithKline	NCT03824236	–	U.S.	64	2019	–
				Plasmodium falciparum malaria	Phase I/II	University of Oxford	NCT02252640	ChAd63 ME‐TRAP, MVA ME‐TRAP	U.K.	48	2015	115
				Malaria	Phase I/II	GlaxoSmithKline	NCT03162614	–	U.S.	154	2017–2018	–
				Malaria	Phase II	GlaxoSmithKline	NCT03276962	–	Ghana, Kenya	1500	2017–2022	–
				Malaria	Phase III	GlaxoSmithKline	NCT02207816	–	Burkina Faso, Kenya, Tanzania	3084	2014–2017	–
				Malaria	Phase II	GlaxoSmithKline	NCT00307021	–	Gabon	180	2006–2007	116, 117
				Malaria	Phase I/II	GlaxoSmithKline	NCT01857869	–	U.S.	64	2013–2014	118
				Malaria	Phase I/II	U.S. Army Medical Research and Materiel Command	NCT00075049	–	U.S.	104	2003–2006	119
				Malaria	Phase II	GlaxoSmithKline	NCT01231503	Engerix‐B, Tritanrix HepB Hib, BCG, OPV, and Rouvax	Malawi	480	2011–2014	120
				Malaria	Phase III	GlaxoSmithKline	NCT02699099	Measles, rubella and yellow fever vaccines	Ghana	721	2017–2020	–

BCG, Bacille Calmette Guerin tuberculosis vaccine; BIPCV, bivalent influenza peptide conjugate vaccine; CPA, cyclophosphamide; DLBCL, diffuse large B‐cell lymphoma; DTPaHibIPV, diphtheria, tetanus, bordetella pertussis, haemophilus influenzae type b, and inactivated polio vaccine; IBD, inflammatory bowel disease; i.d., intradermal injection; i.m., intramuscular injection; IMV Inc., ImmunoVaccine Technologies, Inc.; i.v., intravenous injection; MMR, measles mumps and rubella; NCI, National Cancer Institute; NIAID, National Institute of Allergy and Infectious Diseases; OPV, oral polio vaccine; PRAME, preferentially expressed antigen of melanoma; s.c., subcutaneous injection; VLP, virus‐like particle.

**Table 2:** Nanoparticle‐based immunomodulatory systems for cancer immunotherapy

	Nanoparticle	Payload	Targeting	Stimuli‐sensitivity	Reference
APC activation	PLGA NP	Imidazoquinoline‐based TLR7/8 agonist	–	–	9
	HDL‐mimicking nanodisc	Patient‐derived neoantigen and cholesterol‐modified CpG	–	–	135
	PLGA NP	OVA, Pam3Csk4, and Poly(I:C)	CD40 on DCs	–	136
	Chitosan nanoparticle	Cell lysate from B16 melanoma	Mannose receptor on DCs	–	38
	Lipo‐CpG micelle	CpG	Albumin hitchhiking	–	137
	γPGA‐based CNNP	OVA and poly(I:C)	–	–	138
	PLGA‐based AC‐NP	–	–	–	139
	mBiNE	CRT	HER‐2 on tumor cells	–	140
T cell activation	DMAEMA, PAA, and butyl methacrylate	OVA	–	pH	134
	polyPAA	OVA	–	pH	133
	CNT	MHC1 peptide, anti‐CD28, and PLGA NPs encapsulating IL‐2 and magnetite	–	–	141
	Magnetic nanocluster	MHC1‐OVA, anti‐CD28, and leukocyte membrane fragments	Magnetic navigation	–	128
	PD‐1 receptor‐expressing NV	–	–	–	142
	PEG–PLA NP	CTLA‐4 siRNA	–	–	143
	Platelet‐derived microparticle	Anti‐PD‐L1 Ab	–	–	144
	PEG–PLGA NP	Anti‐PD‐1 Ab and aOX40	–	–	145
	Super‐paramagnetic iron oxide nanoparticle	Anti‐PD‐L1 Ab, anti‐CD3/anti‐CD28 Ab, fucoidan, and dextran	Magnetic navigation	–	129
Regulation of TME	PLGA NP core with lipid shell	Imatinib	Nrp1 receptor on Tregs	–	43
	CDNP consisting of CD and lysine	R848	–	–	146
	NV derived from type 1 macrophage	–	–	–	147
	Carboxyl‐functionalied and aminofunctionalized polystyrene nanoparticle	–	–	–	148
	Super‐paramagnetic iron oxide	–	–	–	149
	Ferumoxytol	–	–	–	150
	HDL NP	–	Scavenger receptor B1 on MDSCs	–	151
	PEGylated LNC	lmGem	–	–	152
	LPH NP	HMGA1 siRNA	Sigma receptor on tumor cells	–	153
	LCP NP	TGF‐β siRNA, tumor antigen, and CpG	–	–	154
	PEG–PLGA NP	SD‐208	PD‐1 on T cells	–	155
	Nanoparticle assembled from DEAP molecule, PD‐L1 antagonist, NGL919, and a substrate peptide of MMP‐2		–	pH and MMP‐2	56
Combination with chemotherapy	NDP based on cationic liposome and HA	CpG and mitoxantrone‐induced DTC	–	–	26
	PEGylated OXA prodrug and homodimer of NLG919		–	pH and reduction	126
	MSNP	DOX, ATRA, and IL‐2	–	–	156
	PTX derivative		–	–	157
	Netrophil‐based cationic liposome	PTX	–	–	158
	PEI–PLGA NP	R300 and DOX	–	MMP‐2	159
Combination with phototherapy	Chitosan‐coated hollow CuS NP	CpG	–	–	57
	PLGA NP	Indocyanine green and imiquimod	–	–	58
	PLGA NPs	APP and HAuNS	–	–	33
	Chimeric peptide PpIX‐1MT	–	–	Caspase	160
	Hollow silica nanoparticle	Catalase and Ce6	Mitochondria	pH	161
	PEG–PLGA NP	Indocyanine green, titanium dioxide, and NH_4_HCO_3_	Mannose receptor on TAM	pH	162
Combination with radiotherapy	PEG‐modified liposome	Catalase and H_2_O_2_	–	–	27
	MnO_2_ NP	ACF	–	ROS	163
	PLGA‐based AC‐NP	–	–	–	139
	PLGA NP	Catalase and imiquimod	–	–	164
Triple combination therapy	Au NR	DOX and CpG	–	NIR	165
	PDA‐coated SGNP	–	–	–	34

#### 2.1.1 Cancer Immunotherapy

*Nanoparticles for Activation of Antigen Presenting Cells*: In initiation of protective immune response, APCs play an irreplaceable role in catching, processing, and presenting antigens to T cells. The antigen presenting efficiency can be significantly improved by adjuvants, including toll‐like receptor (TLR) 4 agonist lipopolysaccharide (LPS), TLR7/8 agonist imiquimod and imidazoquinoline, TLR9 agonist cytosine‐guanine oligodeoxynucleotide (CpG), TLR3 agonist polyinosine‐polycytidylic acid (poly(I:C)), stimulator of interferon (IFN) genes agonist cyclic dinucleotides, and nature‐derived adjuvants, such as pullulan and chitosan.^166^, ^167^, ^168^ In this process, nanomaterials show advantageous function in coencapsulation and simultaneous release of antigens and adjuvants, which is fundamental for APC‐mediated T cell response.^169^ They also protect the payload from being rapidly removed at the injection site. Recently, the advances in the nanotechnology have encouraged the exploration of a surge of nanomaterials for the activation and maturation of APCs. At present, liposome is the most favorable material of immunotherapeutic nanosystems in clinical application due to neglectable toxicity and immunogenicity, including tecemotide, Lipo‐MERIT, iscomatrix, Lipovaxin MM, etc. (Table 1). Besides liposome, several other nanomaterials have also been proved safe in human body. For example, Oncoquest‐L, a cancer vaccine under phase II clinical trial (NCT02194751) is manufactured from an extract of patient's own cancer cells and IL‐2 delivered by proteoliposome. Two cholesteryl pullulan‐based cancer vaccines, CHP‐NY‐ESO‐1 and CHP‐HER2, aroused antigen‐specific immune response against NY‐ESO‐1 and HER2 at the presence of OK‐432 adjuvant in esophageal cancer patients (NCT00291473).^76^

Nanoparticles with appropriate diameters (<500 nm) were liable to be internalized by DCs.^170^ TLR7/8 agonists delivered in poly(lactic‐*co*‐glycolic acid) nanoparticles (PLGA NPs) were easily accumulated and retained in draining LNs and were potent in boosting DC activation.^9^ Codelivery of patient‐derived neoantigen and cholesterol‐modified CpG in a synthesized high‐density lipoprotein (HDL)‐based immunotherapeutic nanodiscs strongly promoted APC maturation and elicited robust T cell response.^135^ The immature DCs incubated with HDL‐antigen/CpG showed increased cell uptake that lasted for as long as 48 h, while those cultured with free antigen and CpG only showed marginal antigen presentation at 6 and 24 h.

The strength of CD8+ T cell response is mainly dependent on the adequate delivery of nanoparticles to APCs. In order to achieve targeted delivery of antigens and/or adjuvants, nanoparticles are decorated with diverse targeting ligands or Abs on the surface.^171^ As a DC‐targeting tumor vaccine in phase I trial stage (NCT01052142), Lipovaxin MM contains melanoma cell‐derived antigens, human IFN‐γ, and an Ab fragment targeting DC‐specific intercellular adhesion molecule‐3‐grabbing nonintegrin. Lipovaxin‐MM showed effective DC‐targeting property in vitro and well tolerance in metastatic melanoma patients, which makes it a promising platform for immunotherapeutic applications.^89^ In a study by Rosalia et al., protein antigens and two adjuvants (a TLR2 agonist and a TLR3 agonist) were encapsulated in PLGA NPs, with CD40 Ab coupling to the surface.^136^ The CD40 Ab specifically bound to CD40 that was abundantly expressed on DCs. The CD40 Ab‐coupled nanoparticles showed fourfold higher internalization in DCs in draining LN than nanoparticles without CD40 targeting ligand. Mannose is another commonly used targeting moiety for recognition of mannose receptor on DCs.^172^ Chitosan nanoparticles conjugated by mannose (Man‐NPs) were developed for delivery of B16 melanoma tumor cell lysate.^38^ After incubation with Man‐NPs, the bone marrow‐derived DCs manifested elevated expression of CD80, CD86, and CD40, indicating enhanced DC maturation. In addition, a larger amount of Man‐NPs migrated to the inguinal LN and remained detectable after 24 h. This migratory advantage of Man‐NPs might be the result of both active and passive targeting effects. PLGA NPs coated by B16 melanoma cell membrane were decorated with mannose for targeted delivery of imiquimod.^37^ After intradermal administration, the nanoparticles showed effective migration to draining LNs and stimulated intense immune responses.

In 2002, Tsopelas and Sutton found that serum albumins could trace to LN and accumulate in APCs.^46^ Later, the strategy named “albumin hitchhiking” approach was successfully used to deliver an albumin binding domain‐decorated amphiphilic vaccine to LNs.^137^ Based on the fact that albumin was capable of transporting fatty acid, Liu et al. conjugated CpG with diacyl lipid to form lipo‐CpG micelles. Lipo‐CpG migrated along with endogenous albumin and achieved eightfold higher accumulation in draining LN than soluble CpG after subcutaneous injection.

**Figure 1:**
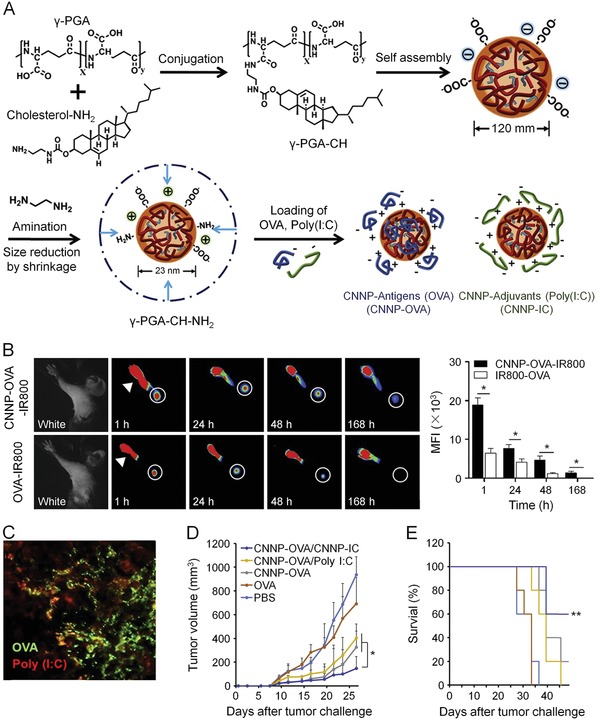
Separated delivery of antigen and adjuvant via γPGA NPs for effective anticancer immunotherapy. A) Synthetic process of CNNPs. B) Near‐infrared (NIR) fluorescence image of IR800‐labeled CNNP‐OVA and IR800‐labeled soluble OVA at different time points after injection through footpad of mice. The axillary LN was circled. Mean fluorescence intensity (MFI) was calculated. C) Histological evaluation of LNs at 24 h after coadministration of CNNP‐OVA‐FITC and CNNP‐IC‐rodamine B. D) Tumor volume and E) survival curve of vaccinated mice after tumor challenge. Reproduced with permission.^138^ Copyright 2017, Elsevier.

Kim et al. presented a different theory that separate delivery of antigens and adjuvants might be a competitive strategy in tumor immunotherapy (**Figure** 1).^138^ One reason was that antigens and adjuvants had different chemical properties, and thus required varied encapsulating materials. On the other hand, it was more practical and reproducible to control the loading amount of each component in isolated delivery systems. One major challenge, however, was to ensure the payloads were colocated to the same site of cells. To test this assumption, they conjugated poly(γ‐glutamic acid) nanoparticles (γPGA NPs) with cholesterol‐NH_2_ into cationic cholesterol‐NH_2_ nanoparticles (CNNPs) for loading of anionic model antigen ovalbumin (CNNP‐OVA) or adjuvant poly(I:C) (CNNP‐IC).^173^, ^174^ The OVA‐loaded nanoparticles were internalized by APCs within 24 h, and the signal of CNNP‐IC overlapped with that of CNNP‐OVA after simultaneous injection. Subcutaneous injection of CNNP‐OVA and CNNP‐IC either before or after tumor challenge could arouse specific CD8+ T cell response and dramatically suppression of tumor growth.

Nanomaterials, such as PLGA, iron oxide nanoparticles, virus‐like particle (VLP), and conjugated polymers, could enhance the cell uptake by APCs and stimulate immune response.^150^, ^175^, ^176^, ^177^ A VLP‐based vaccine, Melan‐A VLP, was used to treat stage III–IV malignant melanoma in a phase II clinical trial (NCT00651703).^94^ The Melan‐A VLP vaccine consisted of a protein shell derived from bacteriophage Qbeta, CpG, and a peptide antigen from melanoma cells. 76% of patients generated more than two‐fold increase in antigen‐specific T cell response. In some other occasions, the nanoparticle can also be engineered as an essential constituent component of the final product with the purpose of facilitating the exposure of tumor antigens to host's immune system. In 2017, Min et al. presented a fascinating idea that nanoparticles could “capture” tumor‐derived protein antigens on the surface and transport them to APCs.^139^ They prepared PLGA NPs modified with varied chemical groups and found that these antigen‐capturing nanoparticles (AC‐NPs) successfully captured the tumor‐specific neoantigens as well as histone proteins and alarmins, indicating great potential to arouse strong antitumor immune response. After injection into irradiated tumors, the AC‐NPs efficiently trafficked to the adjacent tumor‐draining LNs and enhanced the antigen presentation to APCs and the activation of CD8+ T cells. One superiority of this strategy was that it could capture tumor antigens in a patient‐specific manner and provided a possible way of personalized cancer vaccine.

Calreticulin (CRT), a prophagocytic protein, can interact with low‐density lipoprotein receptor‐related protein 1 on APCs to trigger the CRT‐mediated phagocytosis and promote the activation of APCs.^178^, ^179^ Based on this theory, Yuan et al. developed a multivalent bi‐specific nanobioconjugate engager (mBiNE) for targeted immune‐mediated cancer treatment.^140^ A colloidal nanoparticle core was used as substrates to conjugate CRT and a targeting moiety for specific tumor cell recognition. The CRT on the mBiNE recruited professional APCs to induce cancer‐cell clearance and promote antigen processing and presentation. In this way, mBiNE effectively killed tumor cells through both innate and adaptive immune responses.

*Nanoparticles for T Cell Activation*: The presentation of tumor antigens with the participation of major histocompatibility complex I (MHCI) molecules is pivotal for the activation of CD8+ T cell response, which is mainly responsible for recognition of endogenous cytosolic antigens and elimination of infected or cancer cells.^180^ A process named cross‐presentation allows presentation of exogenous antigen via MHCI pathway, where antigens escape from endosome, get processed by cytosolic proteasome, and then form epitope‐MHCI complex before being presented to CD8+ T cells.^181^, ^182^ To protect tumor antigens from degradation by endosome, considerable nanoparticle‐based delivery systems have been developed to escape the endosome for cytosolic antigen release.^183^, ^184^, ^185^

Keller et al. synthesized an endosome‐releasing polymer micelle consisting of a neutral hydrophilic corona segment for conjugation of model antigen OVA, and a pH‐responsive core containing dimethylaminoethyl methacrylate (DMAEMA), propylacrylic acid (PAA), and butyl methacrylate.^134^ When the antigen‐loaded micelles entered endosomal compartments, the acid environment protonated the carboxylate residues of PAA and increased the positive charge in DMAEMA residues. This change led to a transition of hydrophilic conformation to hydrophobic polycation, and then mediated endosomal membrane disruption.^134^, ^186^ The authors found that a majority of OVA delivered by pH‐responsive micelles was able to retain within DC cells in vitro for nearly 1.5 h, while non‐pH‐responsive micelles showed more than 60% antigen exocytosis in 15 min. Mice immunized with endosome‐escaping conjugate showed a higher level of antigen‐specific CD8+ T cells and remarkably improved CD8+ T cell response. More recently, Qiu et al. utilized endosome‐destabilizing polymer polyPAA, featuring pH‐sensitive activity and endosomal membrane destabilizing property to deliver OVA peptide antigen.^133^ The polyPAA/peptide nanoplexes showed extended retention in DC2.4 cells and enhanced presentation on MHCI. This was because polyplex platform kept sustained antigen release by providing an intracellular reservoir and increased the interaction of MHC on DCs and T cell receptor (TCR) on T cells. Intranasal administration of nanoplexes with T cells activator elicited increased CD8+ T cell response and inhibited lung metastases of B16 melanoma.

Another strategy circumvents the necessity of endosome escaping and achieves effective T cells activation and tumor eradication by engineering aAPCs based on modification of nanosized particles, including magnetic beads,^187^ liposomes,^188^ polymer nanoparticles,^19^ and paramagnetic nanoparticles.^189^ The aAPCs basically contain two signals for T cell activation, that is, MHCI‐antigen complex and a costimulatory signal, such as anti‐CD28 and anti‐CD3 Abs.^190^, ^191^ They can be either intravenously injected in vivo or used in ACT ex vivo. For example, a PLGA‐based aAPC was synthesized by functionalization with MHCI‐tumor antigen peptide and anti‐CD28 monoclonal Ab.^190^ The dosage of aAPCs was positively related with the proliferation of antigen specific CD8+ T cells. The advantages of the treatment were also manifested by delayed tumor growth and prolonged survival time. Fadel et al. engineered carbon nanotube (CNT)‐polymer composite as aAPCs containing MHC1 peptide, anti‐CD28, and PLGA NPs encapsulated with interleukin‐2 (IL‐2) and magnetite.^141^ Incubation with CNT aAPCs significantly increased the generation of CD8+ T cells, equivalent to the effect of 1000‐fold less soluble IL‐2. After the CNTs were magnetically separated, a large number of activated CD8+ T cells were transferred to B16 tumor‐bearing mice via peritumoral injection and significantly suppressed tumor growth. Given that the loss of natural membrane functions in aAPCs might interfere with T cell activation, Zhang et al. conjugated MHC1‐OVA and anti‐CD28 to azide‐modified leukocyte membrane fragments and then coated them onto magnetic nanoclusters.^128^ The reinfused CD8+ T cells could be visually monitored by using magnetic resonance imaging (MRI), and the accumulation at tumor tissue could be manipulated with the assistance of magnetic field.

**Figure 2:**
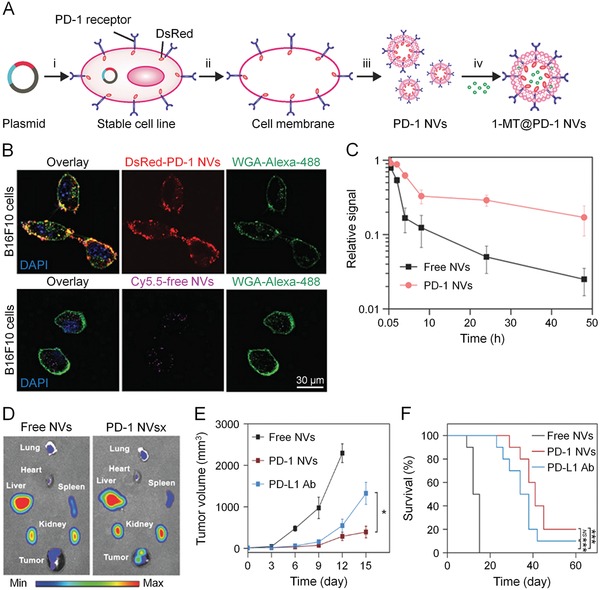
PD‐1 blockade NVs for melanoma immunotherapy. A) Schematic illustration of preparation process of PD‐1 NVs. B) DsRed‐PD‐1 NVs and Cy5.5‐labeled NVs bound with B16F10 cell membrane after incubation for 2 h. WGA Alexa‐Fluor 488 dye highlighted cell membrane. C) Fluorescence of Cy5.5‐labeled free NVs and PD‐1 NVs after intravenous injection. D) Distribution of PD‐1 NVs and free NVs shown by in vivo imagine system (IVIS). E) B16F10 tumor growth curves and F) survival rates of the mice in different groups. Reproduced with permission.^142^ Copyright 2018, John Wiley & Sons.

The immune checkpoint pathways play an important role in preventing the activation of T cells. Until now, a CTLA‐4 Ab (ipilimumab), a PD‐L1 Ab (atezolizumab), and two PD‐1 Abs (nivolumab and pembrolizumab) have been approved by FDA.^192^, ^193^, ^194^, ^195^ Nanoparticles used for delivery of immune checkpoint inhibitors have also shown great antitumor ability in a variety of malignancies. PD‐1 receptor‐conjugated nanovesicles (NVs) were capable of binding PD‐L1‐expressing tumor cells to block the PD‐1/PD‐L1 axis and successfully inhibited the growth of B16F10 melanoma (**Figure** 2).^142^ In this study, Zhang et al. engineered a mammalian cell line that stably expressed PD‐1 receptor on the membrane and then prepared NVs that displayed PD‐1 receptor. PD‐1 NVs manifested longer circulation time in blood as well as more intensive aggregation in tumor tissue compared with free NVs. Moreover, PD‐1 NVs drastically suppressed the tumor growth of mice in comparison with free anti‐PD‐L1 Ab, and the survival time of mice was also prolonged. In another study, CTLA‐4 small interfering RNA (siRNA) was loaded into poly(ethylene glycol)‐*block*‐poly(D,L‐lactide) (PEG−PLA) nanoparticles.^143^ The nanoplatform effectively delivered siRNA into T cells and downregulated the level of CTLA‐4. Systemic administration of this nanoplatform evidently enhanced CD4+ T cells and CD8+ T cells, and decreased the ratio of Tregs. After surgical resection of tumor, the circulating tumor cells are a major cause of cancer recurrence. Worse still, the platelets migrating to the inflamed site would harbor TME with increased expression of PD‐L1 on tumor cells. To enhance the delivery efficacy of anti‐PD‐L1 Ab at surgical bed, Wang et al. conjugated anti‐PD‐L1 Abs to the surface of platelets, which transported to residue tumors and generated platelet‐derived microparticles at activation before releasing anti‐PD‐L1 Abs.^144^ This treatment managed to stimulate the proliferation of CD8+ T cells and reverse the immunosuppressive TME. Moreover, in both melanoma and breast carcinoma models, the tumor recurrence was avoided, and the survival of mice was also prolonged.

The combination of immune checkpoint inhibitor with T cell agonist is a promising method that synergizes the blockade of T cells immunosuppression and activation of T cells. A dual immunotherapy nanoparticle (DINP) conjugating anti‐PD‐1 Ab and T cells agonist antitumor necrosis factor receptor superfamily member 4 (aOX40) was developed to ensure that T cells could simultaneously bind the two agents.^145^ The DINP significantly elevated the rate of T cell activation and showed superior antitumor effect in B16F10 melanoma model and 4T1 breast cancer model compared with treatment with the two free antibodies. In order to enhance the localization of nanoparticles at tumors, Chiang et al. synthesized fucoidan and dextran‐based super‐paramagnetic iron oxide nanoparticles functionalized with anti‐PD‐L1 Ab and anti‐CD3/anti‐CD28 Ab on the surface.^129^ With magnetic navigation, higher tumor accumulation of nanoparticles was achieved and off‐target effect was evidently averted. The findings demonstrated great potential of combining immune checkpoint inhibitors with T cell activators as therapeutic nanomedicine.

*Nanoparticles for Regulation of Tumor Microenvironment*: The antitumor immunotherapy is adversely affected by the immunosuppressive TME due to the presence of Tregs, tumor‐associated macrophages (TAMs), and myeloid‐derived suppressor cells (MDSCs), along with cytokines or enzymes, including transforming growth factor (TGF)‐β, IL‐10, indoleamine 2,3‐dioxygenase (IDO), etc.^3^, ^18^, ^196^ Regulating the TME by interfering the undesired cells or molecules can be a practical method to promote the anticancer immunity. Until recently, many immunotherapies targeting TME have gained intriguing results.^43^, ^56^ The combination of nanotechnology with tumor immunotherapy can be particularly suitable for further improvement in cancer treatment. For example, nanoparticles with targeting ligands are capable of increasing drug accumulation in TME and even increasing internalization by specific immune cell type, thereby overcoming potential systemic hazard and enhancing drug efficiency.^43^ Therapeutics conjugated to nanoparticles could also be transferred to deeper tumor tissue and achieved better efficacy because free form of the agents may only aggregate at the superficial area of tumors.^18^ In some cases, biomimetic nanoparticle platforms are designed to perform intrinsic regulatory effect toward immunosuppressive cells.^197^

Tregs prevent the immune response in TME by hindering APC function and suppressing T cell proliferation and activation.^198^, ^199^ Angiogenetic factors, including vascular endothelial growth factor‐A (VEGF‐A) secreted by Tregs, even help to promote tumor progression.^200^ It was found that specifically depleting the CD25‐expressing Tregs in TME could stimulate the activation of CD8+ T cells and inhibit tumor progression.^201^ In one study, hybrid nanoparticles consisting of PLGA core and lipid shell were synthesized for encapsulation of imatinib, a tyrosine kinase inhibitor that disturbs Treg proliferation.^43^ Then a targeted peptide tLyp1 was conjugated onto the nanoparticle for targeted binding to Nrp1 receptor expressed on most Tregs. The tLyp1‐modified nanoparticles were rapidly internalized into Tregs instead of tumor cells or CD8+ T cells, which led to stronger antitumor effect toward B16 tumors in comparison with nanoparticles without Treg‐targeting peptide.

TAMs have the tendency of polarizing to M2 type at the presence of IL‐10 and TGF‐β. High ratio of M2/M1 would in turn further raise the level of these immunosuppressive cytokines and at the same time downregulate the proinflammatory cytokines, such as IL‐1β and tumor necrosis factor‐α (TNF‐α).^202^, ^203^ In addition, M2 macrophages also hamper the function of DCs and CD8+ T cells, and promote the expression of VEGF for tumor angiogenesis.^204^, ^205^ Since M1 macrophages maintain the antigen‐presentation ability and positive regulation of proinflammatory cytokines, multiple researches attempted to decrease the M2/M1 ratio by repolarizing M2 to M1 macrophages.^206^

**Figure 3:**
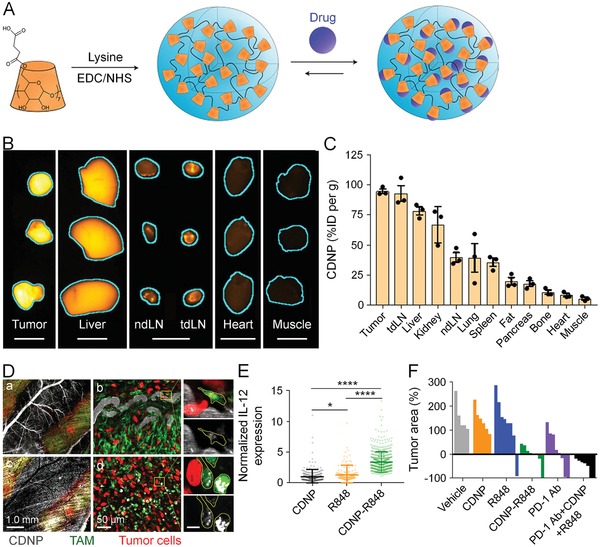
R848‐loaded nanoparticles improved cancer immunotherapy by regulating the polarization of TAMs. A) Preparation of CDNPs by cross‐linking CD with lysine and loading of R848 through host–guest interaction. B) Fluorescence imaging of CDNP accumulation in the tumor and organs at 24 h after administration. C) Quantified distribution of CDNP. D) Uptake of CDNPs by TAMs in tumor bearing mice detected by confocal laser scanning microscopy (CLSM) at (a,b) 60 min or (c,d) 24 h postinjection. Scale bars: 10 µm (b, d, expanded). E) Quantification of IL‐12 in TAMs within tumors at 24 h after different administrations. F) Change of individual tumor area at day 8 after treatment with distinct formulations. Reproduced with permission.^146^ Copyright 2018, Springer Nature.

R848, a dual TLR7/8 agonist, could drive the re‐education of TAMs into M1 phenotype at nanomolar concentration (**Figure** 3).^146^ Succinyl‐β‐cyclodextrin (CD) and lysine was cross‐linked into cyclodextrin nanoparticles (CDNPs) via amide bond, and CDNP was subsequently loaded with R848 through host–guest interaction. CDNP had preferential accumulation at tumors and draining LN. After intravenous injection, CDNP rapidly travelled to the vascular near the tumors within 1 h and internalized into TAMs in 24 h. Administration of CDNP‐R848 in mice not only enhanced the uptake of R848 by TAMs but also promoted re‐education of TMAs to M1 phenotype, characterized by elevated IL‐12 expression. In addition, CDNP‐R848 treatment also suppressed tumor growth and assisted anti‐PD‐1 therapy.

Exposure to specifically engineered nanoparticles without encapsulation of payload could also lead to reprogramming of immunosuppressive macrophages. It was reported that the macrophage‐derived exosomes promoted the adaptive immune response.^197^ The exogenous exosomes secreted by M1 or M2 macrophages were able to stimulate the differentiation of naive macrophages into the corresponding type. M1 exosome could function as an effective adjuvant that triggered CD8+ T cell immunity. Inspired by this finding, Choo et al. acquired NVs derived from type 1 macrophages (M1NVs).^147^ After treatment with M1NVs for 24 h, M2 macrophages showed evident polarization to M1 type characterized by increased expression of M1 marker CD86 and decreased expression of M2 marker CD206. In addition, M1NVs in combination with anti‐PD‐L1 Ab therapy significantly improved the ratio of CD8+ T cells/Tregs and reduced tumor progression in CT26 tumor‐bearing mice model.

Apart from exosome‐mimetic NVs, carboxyl‐functionalized and amino‐functionalized polystyrene nanoparticles also inhibited the polarization toward M2 phenotype.^148^ Similarly, super‐paramagnetic iron oxide nanoparticles were proved to hamper the transition from M1 to M2 TAMs accompanied by decreased expression of IL‐10 and reduced phagocytic activity toward *Streptococcus pneumoniae*.^149^ In 2016, Zanganeh et al. discovered the antitumor effect of ferumoxytol, which was originally approved by the FDA for treatment of iron deficiency.^150^ Ferumoxytol managed to suppress the growth of MMTV‐PyMT breast tumor and inhibit liver metastasis mainly by inducing macrophage polarization to M1 type.

MDSCs are another critical cell type in TME that are related to angiogenesis, tumor progression, and metastasis.^207^, ^208^ MDSCs elevated the expression of anti‐inflammatory cytokines and IDO, leading to Treg activation and antigen‐specific T cell suppression.^209^ Therefore, nanomedicine aiming at eliminating MDSCs could improve cancer immunotherapy. For example, based on the fact that the interaction between HDLs and scavenger receptor B1 expressed on the MDSCs could generate antitumor response, HDL‐like nanoparticles that mimicked nature HDLs were synthesized and showed higher binding ability to scavenger receptor B1.^210^, ^211^ Treatment with HDL NPs significantly minimized the activity of MDSCs and exerted phenomenal antitumor effect characterized by reduced tumor size and metastasis, and prolonged survival time.^151^ Suzuki et al. found that chemotherapy drug gemcitabine posed a specific inhibition on MDSCs without causing damages to T cells or natural killer (NK) cells.^212^ PEGylated lipid nanocapsules (LNCs) containing lauroyl‐modified gemcitabine (lmGem) were developed for targeting depletion of monocytic MDSCs.^152^ A low dose of lmGem‐LNC was sufficient to induce remarkable decrease of monocytic MDSCs both in the spleen and tumor. This suppressive effect could sustain for a period of 48 h, much longer than free lmGem. Furthermore, injection of lmGem‐LNCs 24 h before ACT treatment provided a more suitable microenvironment for T cell proliferation, which facilitated the activation of CD8+ T cells and drastically improved the survival rate of mice. It was reported that the high‐mobility group protein A1 (HMGA1) could stimulate the expression of cyclooxygenase‐2, an influential factor for the production of MDSCs.^213^, ^214^ HMGA1 also causes tumor progression by activating Wnt signaling pathway.^215^ The siRNA that knocked down HMGA1 (siHMGA1) expression was loaded in a liposome‐protamine‐hyaluronic acid (LPH)‐siHMGA1 nanosystem, which was decorated with a targeting moiety on the surface for specific recognition of sigma receptor on CT26‐FL3 cells.^153^ In the highly metastatic colon cancer model, treatment with siHMGA1 NPs significantly increased the frequency of DCs and CD3+CD45+ T cells and reduced the number of MDSCs. In addition, the expression of IL‐10 and TGF‐β was both suppressed, while proinflammatory cytokines, such as IFN‐γ, TNF‐α, and IL‐12a, showed elevated expression levels. The combination therapy of LPH‐siHMGA1 and PD‐L1 blockade effectively inhibited tumor growth and nearly doubled the survival time of individual treatment groups.

TGF‐β promotes tumor progression by suppressing CD8+ T cells activation, inhibiting DC and NK cell function, and most importantly, assisting the proliferation of Tregs.^216^ Xu et al. reported that a lipid‐calcium‐phosphate nanoparticle (LCP NP)‐based vaccine that consisted of tumor antigen and CpG failed to elicit effective immune response in late stage B16F10 melanoma, mainly due to the increased expression of TGF‐β.^154^ In this regard, they delivered TGF‐β siRNA in a LPH nanoplatform to tumor tissue, which significantly reduced the number of Tregs and boosted CD8+ T cell response. The silencing of TGF‐β amplified the immune stimulatory effect of LCP vaccine without inducing systemic toxicity. In another study, a TGF‐β inhibitor SD‐208 was targeted to the cells of interest to restore the immune cell activation.^155^ PEG–PLGA NPs were prepared and conjugated with anti‐PD‐1 on the surface for specific binding to PD‐1‐expressing T cells. Delivery of SD‐208 by PD‐1‐targeting nanoparticles in vivo induced significant tumor inhibition and longer mouse survival time while combination of free anti‐PD‐1 and SD‐208 achieved minimal effect. The therapeutic effect of TGF‐β inhibitor‐loaded anti‐PD‐1 NP was equivalent to one logarithm higher dose of each drug in soluble form.

IDO is expressed by numerous cancers, and high level of IDO is related to accelerated tumor development and metastasis.^217^ It is a critical enzyme that degrades L‐tryptophan (Trp) into L‐kynurenine (Kyn). The depletion of Trp hurdles T cell activation, and the production of Kyn promotes Tregs activation and MDSCs infiltration.^217^, ^218^ Cheng et al. developed a pH‐ and MMP‐2‐sensitive nanosized delivery platform for simultaneous inhibition of IDO and PD‐L1.^56^ They synthesized an amphiphilic peptide, including a 3‐diethylaminopropyl isothiocyanate (DEAP) molecule, a substrate peptide of MMP‐2, and a PD‐L1 antagonist, for assembly with IDO inhibitor NLG919. When entering the acid niche in tumor cells, the protonated DEAP molecule caused unconsolidation of nanoparticle, and high level of MMP‐2 triggered the rapture of substrate peptide, leading to complete disruption of nanoparticle and instant drug release. The precisely controlled drug delivery method potently inhibited the conversion of Try to Kyn and promoted T cell replication. The treatment also delayed tumor growth without causing overt toxicity.

#### 2.1.2 Combination of Immunotherapy and Traditional Managements

Lots of studies showed that the combination of immunotherapy with other anticancer approaches, such as chemotherapy, phototherapy, and radiotherapy, exerted synergistic effect and greatly improved the therapeutic efficacy against a wide range of malignancies.^139^, ^219^, ^220^, ^221^ Chemotherapeutic agents or the external interventions (light and radiation) not only directly exterminate tumors but also participate in the immune process by inducing immunogenic cell death (ICD) of tumor cells.^222^

The concept of ICD proposed in recent years demonstrates that dying tumor cells (DTCs) can generate a mass of antigens and increase the generation of damage associated molecular patterns (DAMPs), such as adenosine triphosphate, CRT, heat shock proteins, and high mobility group box‐1.^223^ DAMPs provide “eat me” signals for antigen recognition and phagocytosis by DCs and trigger activation of adaptive immune response.^224^ More importantly, ICD‐derived tumor antigens can be manipulated by immune system and pose threat to abscopal and metastatic tumors, referred to as “abscopal effect.”^139^, ^225^ For example, the AC‐NPs could bind to tumor‐derived antigens after radiotherapy and present them to DCs.^139^ The secondary tumor, which was protected from radiation also, showed delayed tumor growth, indicating systemic immune response. The AC‐NPs approach also had synergistic effect with anti‐PD‐1 regimen, illustrated by enhanced tumor suppression and improved survival rate. To date, a growing number of successful attempts have revealed the potential of combining ICD inducer with other immunotherapeutic drugs.

*Combination with Chemotherapy*: Chemotherapy is a predominant therapeutic regimen in clinic, but the application is severely hampered by its notorious side effects and tumor recurrence.^226^ Chemotherapy combined with immunotherapy can well relieve the toxicities and also improve its treatment effect. In the chemoimmunotherapy, low dose chemotherapy is capable of inducing ICD of tumor cells and leading to tumor antigen release, so severe side effects are averted. At the same time, immunoregulatory agents provide a preferable environment to ensure the highly efficient antigen presentation and the activation of APCs and cytotoxic T cells.^227^ Many studies that encapsulate the chemo/immunotherapeutic agents in nanosized drug delivery systems have turned out successful.^26^, ^126^, ^228^

For example, the TLR9 agonist CpG was loaded into a nanodepot platform (NDP) composed of cationic liposomes and thiolated hyaluronic acid (HA).^26^ The CpG‐NDP was then conjugated onto the surface of immunogenic DTCs, induced by mitoxantrone, an anthracenedione antitumor agent. Experiment on B16F10 mice model showed that DTC‐CpG‐NDP vaccination significantly stimulated the generation of tumor antigen‐specific CD8+ T cells and strongly protected against melanoma challenge.

**Figure 4:**
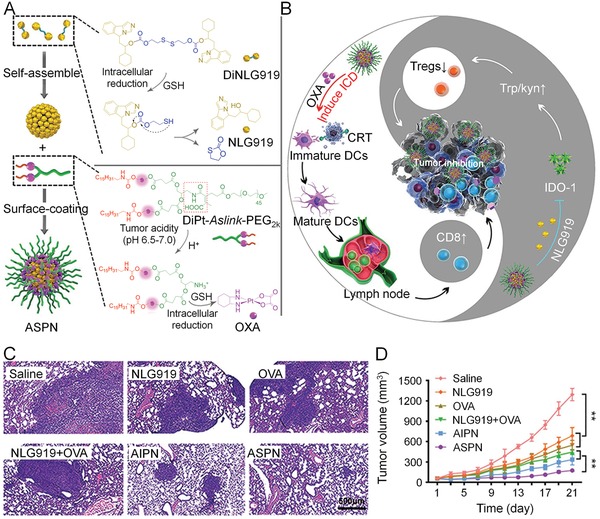
Prodrug nanoparticles containing OXA and NLG919 improved immunotherapy by dual modulation of tumor immune microenvironment. A) Self‐assembly of ASPN from stimuli‐responsive OXA prodrug and NLG919 prodrug. B) Schematic illustration of synergistic antitumor effect from activated CD8+ T cells and suppressed Tregs. C) H&E staining of lung metastasis in 4T1 tumor‐bearing mice at the end of study. D) Survival curve of 4T1 tumor‐bearing mice during therapy. Reproduced with permission.^126^ Copyright 2018, John Wiley and Sons.

ICD can also be induced upon the administration of chemotherapeutic agents in vivo. Simultaneous administration of ICD inducers and immunomodulatory agents remodels the TME and significantly amplifies the antitumor efficacy. Recently, a binary prodrug nanoparticle was constructed from an acid and reduction dual‐sensitive PEGylated oxaliplatin (OXA) prodrug and a reduction‐responsive NLG919 prodrug (**Figure** 4).^126^ At the acid pH of TME, PEG corona was cleaved and nanoparticle surface switched to positive charge for deeper penetration and increased cell uptake. Then OXA prodrug and NLG919 prodrug were safely activated in the reduction environment of TME. The stimuli‐sensitive nanosystem‐based treatment drastically inhibited the primary tumor growth and lung metastasis of 4T1 tumor. In another study, doxorubicin (DOX), all‐trans retinoic acid (ATRA), and IL‐2 were codelivered in the biodegradable hollow mesoporous silica nanoparticle (MSNP) to treat B16F10 melanoma.^156^ ATRA induces differentiation of MDSCs into matured DCs and macrophages, and IL‐2 stimulates the proliferation and activation of CD8+ T cells.^229^ In this combinational treatment strategy, a reduced dosage of DOX (2.5 mg kg^−1^, lower than conventional injection dose of 5 mg kg^−1^) was able to generate adequate immunogenic tumor peptide antigens, so the systemic toxicity was minimized. This nanoparticle platform significantly suppressed tumor growth and lung metastasis of melanoma, and also exhibited excellent safety.

A portion of traditional chemotherapeutics unexpectedly pose an immunoregulatory effect on immune cells. For example, the chemotherapeutic drug paclitaxel (PTX) exerts a modulatory effect on the macrophage polarization to M1 phenotype at low concentrations.^157^ Unlike free PTX, NP‐PTX could be efficiently endocytosed by macrophages and stimulate macrophage polarization in a dose‐dependent manner without causing obvious toxicity to immune cells. Although the mechanism underlying this phenomenon was not clear, it presented an unconventional strategy to research into the immunomodulatory function of chemotherapeutics.

Researchers have also explored the immunomodulatory effect of nanomaterial to improve the therapeutic efficacy of encapsulated drugs. For example, nanoparticles can be engineered for targeting delivery of drugs to tumor cells depending on the immune environment. Xue et al. encapsulated PTX‐loading cationic liposomes into neutrophils, which intrinsically penetrate the glioma site and migrate along the chemotactic gradient of inflammatory factors.^158^ Intravenous injection of the neutrophil‐based nanoparticle formulation delivered PTX into inflamed brain after surgery and effectively prevented glioma recurrence. In addition, TME could also be remodeled for favorable drug delivery efficiency. The tumor‐associated platelets protect the integrity of tumor blood vessels and hamper the accumulation of nanomedicines at tumor site.^230^, ^231^ To increase the perfusion and retention of drug‐loaded nanoparticles, Li et al. developed a dual drug delivery system containing a polymer core of polyetherimide (PEI)–(PLGA)_2_ that was loaded with antiplatelet Ab R300, chemotherapeutics DOX, and a shell layer with MMP‐2‐sensitive peptides.^159^ Depletion of platelets created openings in the tumor vessels for easier entrance of the drug‐loaded core nanoparticles. With increased DOX accumulation at tumor tissue, the tumor progression and metastasis of 4T1 breast tumor were considerably suppressed. Collectively, nanoparticles can be modified or engineered according to specific clinical situation of certain tumor types. The aforementioned nanoparticle‐based delivery systems are also applicable for delivery of a variety of anticancer drugs, molecular targeted drugs, and anti‐inflammatory drugs.

*Combination with Phototherapy*: Phototherapy is an efficient tumor treatment method based on the manipulation of NIR with a wavelength ranging from 650 to 1350 nm.^232^ The two most important forms of phototherapy for cancer treatment are photothermal therapy (PTT) and photodynamic therapy (PDT). Photothermal agents used in PTT convert NIR to thermal energy. Through increasing the temperature of targeted region, PTT induces necrosis of tumor cells and causes ablation of tumor with little invasion to nearby tissues.^233^ PDT employs photosensitizers to initiate photochemical reaction at certain wavelength of light. It requires the existence of abundant oxygen to provoke the release of cytotoxic ROS, such as singlet oxygen (^1^O_2_) and H_2_O_2_.^234^

Recently, it has been found that apart from direct cytotoxicity toward tumor cells, phototherapy can also stimulate ICD of tumor cells and release DAMPs in TME, thus inducing antitumor immune response.^235^, ^236^ According to this theory, Guo et al. engineered a chitosan‐coated hollow CuS NP encapsulating CpG, which achieved effective tumor ablation mainly through three levels.^57^ First, the primary tumor was largely eradicated by photothermal effect under NIR radiation. Second, the tumor antigens originated from disrupted cancer cells were presented to CD8+ T cells by APCs and aroused systemic tumor‐specific cytotoxicity, eliciting suppression toward the distant unirradiated tumors. Third, the CpG in nanoparticles was drained to LN and internalized by DCs, which further boosted the antigen‐presentation efficiency. The synergy of phototherapy and immunotherapy endows superior therapeutic effect compared with single treatment.

Notably, this method can be especially beneficial for inhibition of metastatic tumors, because metastatic tumors are usually located deep beneath the skin where light is not able to penetrate. For example, encapsulation of indocyanine green, a photothermal agent, and imiquimod into PLGA NP showed efficient in situ tumor inhibition upon NIR laser.^58^ A combination therapy with CTLA‐4 blockade for Treg suppression further eradicated tumor residues and reduced lung metastasis. More recently, Luo et al. reported a combination therapeutic platform that encapsulated anti‐PD‐1 peptide (APP) and hollow gold nanoshell (HAuNS) into PLGA NPs.^33^ Under NIR irradiation, the HAuNS generated PTT effect in tumor site and simultaneously released APP. In a triple‐cell system containing T cells, DCs, and 4T1 cells, mixed cells incubated with HAuNS‐ and APP‐loaded PLGA NPs plus CpG under laser irradiation secreted a significantly higher level of IFN‐γ and TNF‐α than soluble CpG and APP, indicating stronger T cell proliferation and activation. Combining PTT with CpG treatment showed the most potent antitumor effect against untreated distant tumors and metastatic tumors in the lung. This was because the tumor antigens produced by NIR laser and adjuvant CpG stimulated the maturation of DCs, activating T cell‐mediated immune response and arousing systemic immunity.

**Figure 5:**
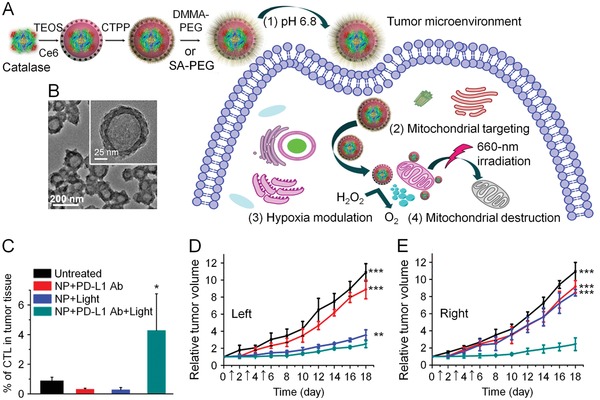
Smart nanoreactor enhanced antitumor effect to both primary and distant tumors by combination of PDT with PD‐L1 blockade. A) Synthetic process of DPEG‐coating mitochondria‐targeting nanoreactor loaded with catalase and Ce6, and schematic depiction of using nanoreactor to improve PDT process. B) Transmission electron microscopy (TEM) image of synthetic nanoreactor. C) Changes of tumor volume and D) cytotoxic T cell infiltration in primary tumors after treatments. E) Tumor growth curve for nonirradiated tumors. Reproduced with permission.^161^ Copyright 2018, American Chemical Society.

Versatile nanoplatforms have been continuously developed for targeted delivery of phototherapy agents to tumor cells or cell organelles in order to optimize tumor‐specific ablation and minimize the side effects. Song et al. designed a nanoparticle formed by photosensitizer PpIX and IDO inhibitor 1‐methyl‐Trp (1MT), connected by a caspase‐sensitive peptide sequence.^160^ After accumulation at tumor site via the EPR effect, PpIX−1MT NPs generated ROS under the light irradiation, rapidly killing tumor cells and producing tumor antigens. Simultaneously, the elevated production of caspase‐3 in apoptosis tumor cells liberated 1MT to block the IDO pathway and averted the immunosuppressive TME. This platform could not only significantly suppress the primary tumor growth of CT26‐bearing mice, but also effectively eradicate the metastatic tumor in the lung. In another study, Yang et al. developed a charge‐convertible and mitochondria‐targeting smart nanoreactor for combination of PDT and immune checkpoint blockade therapy (**Figure** 5).^161^ Catalase, a H_2_O_2_‐sensitive enzyme, and a photosensitizer Ce6 were loaded into a hollow silica nanoplatform as nanoreactor. The nanoparticle was further decorated with a molecule specifically targeting mitochondria, (3‐carboxypropyl)triphenylphosphonium bromide, and a surface coating of PEG, yielding a pH‐responsive polymer nanoplatform DPEG. The polymer exhibited positive charge in acid TME that improved the local retention and the uptake by tumor cells. Afterward, the catalase‐ and Ce6‐loaded nanoparticles were drawn to mitochondria through targeting pathway. Under external NIR, the nanoreactor released catalase and decomposed the abundant H_2_O_2_ in tumor cells into oxygen for ^1^O_2_ production, which enhanced PDT effect. Since mitochondria is a ROS‐sensitive organelle, ^1^O_2_ can mediate cell apoptosis in the early stage of PDT.^60^ In a 4T1 tumor model, nanoreactor synergized with anti‐PD‐L1 Ab showed elevated antitumor effect and upregulated CD8+ T cell infiltration. Most interestingly, the mice also showed delayed progression in nonirradiated distant tumors, due to the systemic immune response.

In addition to providing a source of tumor antigens, phototherapy could also participate in immunoregulatory therapy through other mechanisms. Shi et al. found that the intracellular photogeneration of ROS was able to regulate the reprogramming of TAMs toward M1 phenotype.^162^ They synthesized endosome‐escaping and TAM‐targeting PEG–PLGA NPs for encapsulation of photosensitizers with NH_4_HCO_3_. The nanoparticles entered macrophages through mannose‐mediated endocytosis, and then the acid environment of endosome elicited the production of CO_2_ and NH_3_ from NH_4_HCO_3_, which rapidly damaged the endosome membrane and facilitated the release of photosensitizers into cytoplasm. Of note, ROS generated at 808 nm laser radiation could rapidly switch M2 TAM to M1 type indicated by increased level of M1 marker inducible nitric oxide synthase (iNOS) and cytokine IL‐12, as well as decreased expression of M2 marker CD206 and lowered level of IL‐10. Moreover, the antigen presentation property of TAMs was restored and the CD8+ T cell response was also enhanced.

Additionally, the ICD induced by phototherapy is tunable by adjusting NIR to maximize the therapeutic effect. In other words, it is not necessary to follow “more is better” pattern in tumor eradication. For example, Sweeney et al. discovered a thermal “window” of ICD in Prussian blue nanoparticle‐based PTT treatment of neuroblastoma, confirming that there was an optimal temperature range and thermal dose window to generate the most efficient ICD.^237^ Although higher temperature induced by higher dosage of PTT agent positively contributed to more efficient local tumor suppression, the immune response elicited by ICD had the greatest impact on the elimination of disseminated tumors. In this study, mice showed the highest survival rate after inoculation with Neuro2a cells that endured optimal thermal dose determined in vitro.

*Combination with Radiotherapy*: Ionizing radiation is considered to be an effective local cancer therapy that induces direct cell death by causing DNA damage, cell membrane and organelles dysfunction, and disorder of gene and protein expression.^238^ Interestingly, the irradiated tumor cells go through ICD that is featured by increased tumor antigen quantity and release of DAMPs, permitting the activation of APCs and damage of abscopal tumor cells through T cell‐mediated immune response.^239^ This abscopal effect is reported to assist in eradication of metastatic tumors outside the radiation region.^240^ In this regard, abscopal effect of radiotherapy can be hopefully boosted in combination with immunotherapy. However, on the downside, radiotherapy can also exacerbate the immune energy in TME by stimulating the expression of immunosuppressive cytokines TGF‐β and IL‐10, and recruiting M2 TAMs and Tregs to the radiated site.^206^, ^241^ This phenomenon was reported to be related to the hypoxia condition accompanied by rapid oxygen consumption and ROS production during radiotherapy.^242^ Hypoxia also helps prevent DNA damage and promote VEGFA production, leading to high tumor recurrence rate after radiotherapy.^243^ To this end, Song et al. developed a PEG‐modified liposome to separately encapsulate catalase and H_2_O_2_ to acquire self‐supplied oxygen.^27^ The catalase‐nanoparticle and H_2_O_2_‐NP were intravenously injected into mice successively at an interval of 4 h to enhance tumor oxygenation, which offered long‐lasting effect to reduce the decomposition of endogenous H_2_O_2_, thus relieving the hypoxia burden in TME. Upon X‐ray irradiation, this treatment method evidently stimulated the TAM transition toward M1 type and lowered the secretion of IL‐10. The CTLA‐4 checkpoint inhibition remarkably improved the level of CD8+ T cell infiltration and downregulated the Tregs ratio in TME. The synergistic radioimmunotherapy achieved significantly enhanced antitumor effect with alleviated hypoxia condition. In another study, catalase and imiquimod were coloaded into PLGA NPs to combine tumor hypoxia relief with robust immune response in TME.^164^ The authors developed core–shell PLGA NPs by classical double emulsion method where catalase was encapsulated inside the hydrophilic core and imiquimod was loaded into the shell. Under local X‐ray radiation, catalase rapidly reversed hypoxia condition and alleviated immunosuppressive TME. At the same time, tumor antigens from dying tumor cells were captured by imiquimod‐activated APCs and triggered robust antitumor immune response against both primary and secondary tumors. Furthermore, a combination with CTLA‐4 blockade completely eliminated primary CT26 colorectal tumors, and also drastically improved the abscopal effect by increasing cytotoxic lymphocyte (CTL) infiltration in distant tumors.

**Figure 6:**
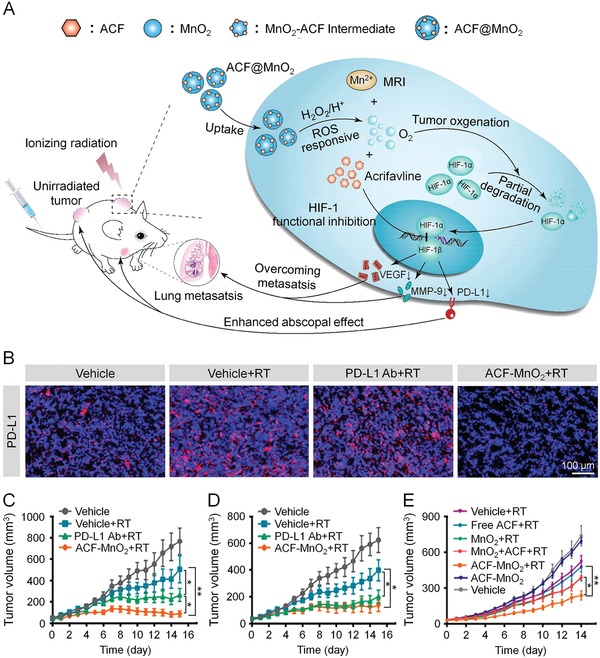
ROS‐responsive nanoplatform for enhanced radiation therapy and abscopal effect. A) Mechanism of ACF‐MnO_2_ NPs eliminating primary and abscopal tumors by tumor oxygenation and HIF‐1 dysfunction. B) Immunofluorescence images of primary tumor slices stained with PD‐L1 Ab. C,D) Tumor volume changes of C) primary and D) distant tumors in CT26 mice model. E) Primary tumor growth in 4T1‐bearing mice after different treatments. Reproduced with permission.^163^ Copyright 2018, American Chemical Society.

To explore another way to relieve tumor hypoxia, Meng et al. conjugated a potent inhibitor of hypoxia inducible factor‐1 (HIF‐1), acriflavine (ACF), to MnO_2_ NP via a ROS‐responsive bond for radiation therapy (**Figure** 6).^163^ At the reaction with overexpressed H_2_O_2_ in TME, MnO_2_ was released and generated oxygen molecules to relieve hypoxia and promoted radiation sensitization. Additionally, Mn^2+^ could be used to guide radiation therapy through MRI. In the meantime, ACF could hamper the formation of HIF‐1, which was responsible for tumor resistance to radiation and upregulated VEGF secretion. More importantly, HIF‐1 was also involved in hypoxia‐dependent PD‐L1 gene transcription. The researchers established a mice model bearing primary and abscopal CT26 tumors, and only primary tumor was given radiation therapy. It was observed that PD‐L1 level in both tumors was notably decreased, leading to enhanced cytotoxic T cell activation and IFN‐γ production, and tumor mass was significantly reduced. Similar effect was observed in a 4T1 metastatic tumor model, where ACF‐MnO_2_ NP dominated other treatments with free ACF or without ACF.

*Triple Combination Therapy*: Combination therapy strategy is able to synergize the therapeutic effects of more than two treatment mechanisms in one nanosystem, thus further improving the antitumor outcomes. For example, NIR‐responsive gold nanorods (NRs) were produced as a deliver vehicle for DOX and CpG.^165^ The self‐complementary CpG was assembled into Y shape and immobilized onto gold NRs. DOX, carrying flat aromatic rings and positive charge, was subsequently intercalated into CpG molecules. Upon NIR irradiation at 808 nm, DOX and CpG were controllably released at the tumor site. It was found that the accumulation of DOX in tumor was markedly increased compared with free DOX. CpG rapidly activated APCs and promoted the release of proinflammatory cytokines TNF‐α and IL‐6. This photo‐chemo‐immuno‐nanoplatform resulted in evidently enhanced antitumor efficacy against H22 hepatoma without causing significant weight loss.

**Figure 7:**
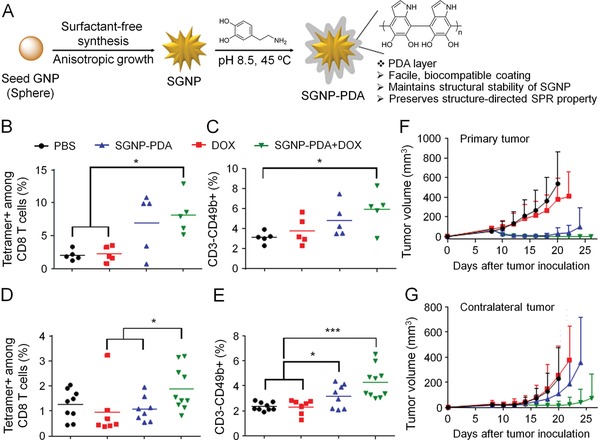
Chemo‐photothermal therapy potentiated antitumor immunity against primary and metastatic tumors. A) Synthesis of SGNPs with PDA coating. B–E) Frequencies of antigen‐specific CD8+ T cells and NK cells in tumor‐infiltrating LN in B,C) primary tumors and D,E) contralateral tumors. F,G) Tumor volume curve of F) treated primary tumors and G) untreated contralateral tumors. Reproduced with permission.^34^ Copyright 2018, Springer Nature.

Nam et al. demonstrated that chemo‐phototherapy alone was competent to elicit immune activation in local TME and amplified antitumor immunity against disseminated and metastatic tumors (**Figure** 7).^34^ In this study, they developed polydopamine (PDA)‐coated spiky gold nanoparticles (SGNPs) with anisotropic morphology and large surface area, both of which were important parameters for high photothermal efficiency. PDA coating functioned as a passivation layer to reduce thermal diffusion and shape reconstruction. After intratumoral injection, tumor debris derived from a single round of PTT synergized with ICD of tumor cells induced by sub‐therapeutic dose of DOX (1.36 mg kg^−1^) markedly increased the infiltration of antigen‐specific CD8+ T cells and NK cells in TME. This treatment achieved complete tumor regression in both local and untreated contralateral tumors in a CT26 colorectal cancer model. Besides, tumor re‐challenge was successfully rejected on account of the immunological memory.

### 2.2 Immunomodulatory Nanoparticles against Infectious Diseases

Many attractive characteristics of nanoparticles in cancer immunotherapy are also applicable to prevent or resist bacteria or virus infections, such as human immunodeficiency virus (HIV), influenza, encephalitis, hepatitis, Ebola, pneumonia, etc.^21^, ^244^, ^245^, ^246^, ^247^, ^248^ In the prevention of these diseases, anti‐infective vaccines used specific antigenic components instead of whole microbes in order to increase immune efficiency. However, these antigens are more easily degraded by enzymes and eliminated in blood circulation. Moreover, they usually require the assistance of adjuvants to effectively activate immune systems. Additionally, DNA vaccines also show great potential in recent years, but their usage in clinical practice is limited by poor safety and low efficiency. Nanotechnology offers a possibility for a new generation of anti‐infective vaccines. At present, virosome and liposome‐based nanovaccines against infectious diseases have shown good efficacy in human body (Table 1). Until now, two nanoparticle‐based vaccines, Inflexal V and Epaxal, have been approved by FDA for the prevention of malaria, influenza, and hepatitis A.^249^ With suitable sizes, nanoparticles could deliver antigens and adjuvants to immune cells by either encapsulation or surface conjugation. Nanoparticles are also engineered as reservoir for slow release of antigens to increase the exposure to APCs. As for DNA vaccine, nanoparticles provide a nonviral delivery strategy that transport genetic material in a site‐specific manner. Numerous evidences have confirmed the encouraging effects of nanotechnology‐based vaccine formulations against infectious diseases, which benefit from improved delivery efficiency, convenient nanoparticle engineering, and intrinsic adjuvant function.^250^ Immunomodulatory systems that use nanoparticles for prevention and treatment of infectious diseases are listed in **Table** 3.

**Table 3:** Nanoparticle‐based immunomodulatory systems against infectious diseases

	Nanoparticle	Payload	Targeting	Reference
Vaccine for HIV	Ag NR modified with PVP and PEG	–	–	251
	Nanofiber self‐assembled from NMe	HIV DNA	–	252
	Nanofiber self‐assembled from a mixture of NMe, HIV DNA, and ALP		–	246
	PLGA NP core coated with CD4+ T cell plasma membrane	–	Gp120 on HIV	253
	LNC attached with HIV‐specific CTL	IL‐15 superagonist	Antigen on HIV‐infected cells	254
	Glycosylated HIV antigen nanoparticle		–	255
Vaccine for influenza	Gold NR	Low‐molecular‐weight poly(I:C)	–	8
	Two‐layer protein nanocluster assembled from 3HMG		–	256
	Protein nanoparticle of 4M2e coated with stalk domain of HMG		–	247
	NPep core and 4M2e coating layer		–	257
	γPGA/chitosan nanogel		–	248
Vaccine for bacteria infection	Au NP	Flagellin	–	258
	Au NP loaded with capsular polysaccharide antigens	T‐helper peptide OVA	–	245
	RBC membrane‐coated PLGA NP	α‐hemolysin	–	259, 260
	RBC membrane‐coated PLGA NP	Virulence factors from hSP	–	261
	Au NP coated with OMV from bacteria		–	262
Vaccine for other infectious diseases	Plasmonic Au NP	DNA plasmid of hepatitis C virus	–	244
	PLGA–PLL/γPGA NP	Ebola DNA vaccine	–	21
	MDNP consisting of dendrimer and lipid‐PEG	Replicon mRNA	–	22

#### 2.2.1 Nanoparticle Vaccine for HIV

HIV causes acquired immunodeficiency syndrome by infecting and destroying the host's immune cells, including CD4+ T cells and macrophages.^263^ Although highly active antiretroviral therapy has been proved effective in stabilizing the symptoms and extending patients' survival time, it requires a lifetime medication and treatment interruption will result in uncontrolled viral rebound.^264^ Moreover, long‐term treatment can also develop a variety of side effects, including lipodystrophy, hyperlipidemia, and damage to the liver and immune system.^265^ Worse still, the HIV virus could only be reduced but not completely removed from infected patients. Scientists have suggested that HIV vaccine might be a new hope to eliminate virus by boosting immune response. However, the immunity generated by neutralizing Abs targeting HIV envelope glycoproteins is far from satisfactory.^266^ In this case, nanomaterials can enhance the therapeutic effect of HIV vaccine as adjuvants or carriers. For example, silver nanoparticles (Ag NPs) and polyvinylpyrrolidone (PVP) both have immunostimulation property, and nanosilver also has intrinsic HIV inhibition effect. Silver NRs modified with PVP and PEG on the surface evidently enhanced the IgG response and T cell proliferation against HIV.^251^ The authors had a preference for NRs but not nanospheres mainly due to safety consideration. They found the uptake of silver NRs by host cells was lower than that of nanospheres, indicating less toxicity. In another study, HIV DNA vaccine was delivered in nanofiber prepared by self‐assembly from an immune active peptidic precursor NMe in vitro with addition of alkaline phosphatase (ALP).^252^ It showed that the HIV DNA compressed in a compact nanofiber structure was more effectively internalized by APCs. Besides, the nanofiber itself as adjuvant enhanced antigen‐specific T cell response and maturation of B cells. However, this method was rather inconvenient because nanofibers had to be prepared every time before vaccination. Later on, in order to simplify this approach by omitting the require for exogenous ALP, they mixed DNA vaccine and NMe together with ALP before injection to mice.^246^ In this way, the vaccine‐loaded nanofibers could be formed in situ and had the same immunological effect as those formed in vitro.

Nanoparticles wrapped with membranes of natural cells, including red blood cells (RBCs), bacteria, platelets, cancer cells, etc., have exhibited cell‐mimicking properties.^267^ Similarly, T cell‐membrane‐coated nanoparticles resemble parent cells in surface antigens like CD4 receptor, which combines to HIV envelope glycoproteins gp120 and initiates virus entry and fusion. Based on this hypothesis, CD4+ T cell‐derived plasma membrane was coated onto PLGA cores as a decoy with selective binding ability to gp120 on HIV.^253^ In this way, the membrane‐functionalized nanoparticles neutralized virus and prohibited gp120‐induced attack to host cells. In addition to CD4+ T cells, CTLs with complete function were also engineered to conjugate immunomodulatory agents‐loaded nanoparticles for targeted drug delivery.^254^ In this study, the HIV‐specific CTLs were attached to LNCs encapsulating IL‐15 superagonist. CTLs recognized the antigens expressed on the surface of infected cells and initiated perforin‐mediated cytotoxicity, which led to membrane destruction of both HIV‐infected cells and LNCs, followed by the release of IL‐15 agonist. This LNC‐based vaccine showed an excellent antiviral effect in a mouse model of HIV.

New vaccines made of nanoparticulate HIV immunogens were also developed and showed enhanced B cell, T cell, and germinal center responses, as well as increased generation of neutralizing Abs.^268^ Tokatlian et al. discovered that glycosylated HIV antigen nanoparticles were more likely to aggregate in germinal centers and triggered Ab responses compared with monomers.^255^ The mechanism was associated with mannose‐binding lectin‐mediated innate immune recognition and dense arrays of immunogen glycan. Their work might reverse the conventional option that dense envelope glycan would impair Ab response and present a new theory for vaccine design.

#### 2.2.2 Nanoparticle Vaccine for Influenza

The development of influenza vaccine has made a great progress since the H1N1 influenza pandemic during 2009–2010. Current vaccines are effective in preventing both seasonal and pandemic influenza infections.^269^ However, due to the constant shifty nature of influenza virus antigens, it is still a major challenge for timely control of this infectious disease. The nanomaterial‐based vaccine provides opportunities for a novel generation version to enhance immune protection against influenza viruses.^270^

The immunogenicity of vaccine can be drastically enhanced by delivering adjuvants and antigens via nanoparticles.^271^ Studies have highlighted the benefits of organizing immunomodulatory adjuvants onto Au NPs to enhance cell uptake and improve immune activity in cancer immunotherapy.^272^ Inspired by this finding, Tazaki et al. immobilized low‐molecular‐weight poly(I:C) onto gold NRs and inoculated them into mice together with influenza virus antigen hemagglutinin (HMG) through intranasal administration.^8^ Compared with the current standard subcutaneous or intramuscular vaccination methods, intranasal administration can be a competitive way because of its simulation to mucosal immune response and needle‐free injection, but it has a higher requirement for adjuvant activity. In this study, the gold NR‐poly(I:C) achieved improved adjuvanticity by eliciting strong mucosal IgA Ab activity against viral infection even at low antigen doses.

Compared with soluble antigens, the antigens loaded in nanoparticle can be more easily recognized and internalized by APCs and stimulate their maturation.^256^ In addition, nanoparticles as an antigen reservoir maintain certain immunogen concentration in the draining LNs for a longer time than soluble antigen. In one study, the trimeric form of HMG (3HMG) from H7N9 virus was assembled into nanoclusters before being cross‐linked with an extra 3HMG layer on the surface.^256^ The two‐layer protein nanocluster not only multiplied the immunogenicity of HMG, but also achieved prolonged and continuous release of antigenic proteins into immune cells. The results demonstrated that mice after intramuscular immunization showed increased serum level of HMG‐specific IgG, which protected them from later challenge with live H7N9 virus. In another study, Deng et al. isolated the stalk domain of HMG, which was more conservative than head domain, to envelope a protein nanoparticle core desolvated from four tandem copies of matrix protein 2 ectodomain (4M2e, a conservative amino acid segment of influenza A virus) from four different species to broaden the protection range.^247^ The nanoparticle core showed strong immunogenicity and the coating layer mimicked the size and surface antigens of influenza virus. It was found the layered protein nanoparticles elicited Ab‐dependent immune protection against H1N1 and H3N2.

**Figure 8:**
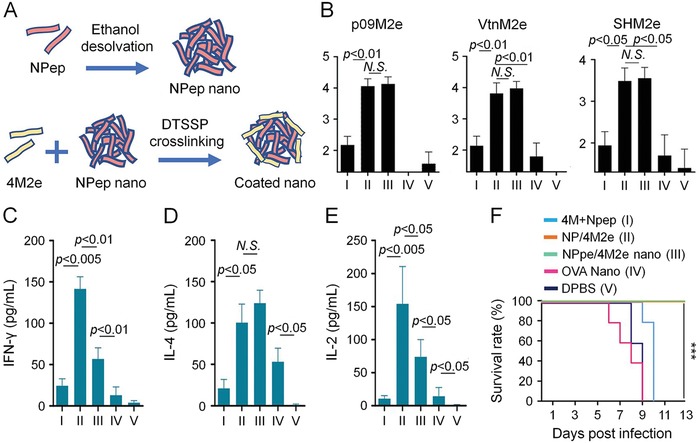
Double‐layered polypeptide nanoparticles induced potent protection against influenza. A) Schematic illustration of core nanoparticle fabrication and double‐layered nanoparticle formation. B) Evaluation of M2e IgG binding ability to p09M2e, VtnM2e, and SHM2e. C–E) Examination of splenocyte cytokine secretion, including C) IFN‐γ, D) IL‐4, and E) IL‐2. F) Survival rate of immunized mice challenged with H5N1 virus. Reproduced with permission.^257^ Copyright 2018, National Academy of Sciences.

Later this year, the same group of researchers developed a peptide‐only double‐layered nanoparticles based on a nucleoprotein peptide (NPep) core derived from H3N2 strain and 4M2e coating layer for skin vaccination by dissolvable microneedle patch (**Figure** 8).^257^ Mice immunized with NPep/4M2e via intramuscular injection showed potent IgG response against M2e and strong cellular response with elevated IFN‐γ, IL‐4, and IL‐2 levels. The M2e Ab had cross‐reactivity with other M2e peptides of H1N1 (p09M2e), H5N1 (VtnM2e), and H7N9 (SHM2e), which was further proved by that nanoparticle vaccination successfully protected mice from intranasal challenge of H5N1 virus. It was shown that microneedle patch achieved comparable immune protection as intramuscular injection. The microneedle patch was a promising vaccine route due to its convenience and painless self‐administration, as well as its ability to boost immunity through the abundant amounts of APCs in the skin tissues.

Some studies take advantage of the intrinsic adjuvant properties of nanoparticles in vaccination. For example, γPGA/chitosan nanogel was used as adjuvant for pandemic H1N1 influenza vaccine.^248^ Mice vaccinated with H1N1 antigen and γPGA/chitosan nanogel showed a higher level of IgG titers and H1N1‐specific CTL, revealing that γPGA/chitosan nanogel was a more potent adjuvant than aluminum compound, a well‐known vaccine adjuvant in human. The same result was duplicated in a ferret model. γPGA/chitosan vaccine effectively protected ferrets from intranasal administration of H1N1 virus. In addition, it also induced long‐term virus‐specific T cell memory and exerted heterosubtypic protection against H3N2 virus infection.

#### 2.2.3 Nanoparticle Vaccine for Bacteria Infection

At the approach of post‐antibiotic era, multiple adverse effects and antibiotic resistance have become a serious alarm for antibiotic usage against bacteria infection.^273^ In this regard, antibacterial vaccines hold promises to reduce exposure to antibiotics and in the meantime manage bacteria infection.^274^ Nevertheless, the development of effective antibacterial vaccines is still a tricky task due to the highly complex protein compositions of microbes and their defense mechanisms to evade host immune surveillance.^275^ The nanomaterial‐based vaccines have been introduced to activate host immune response for effective bacteria defense.

Nanoparticles, including inorganic nanoparticles and polymer nanoparticles, as efficient delivery vehicles have shown effective immunization in bacteria‐infection defense. Au NPs are preferable for nanovaccine preparation due to good biocompatibility, simple synthetic process, and most importantly, the adjuvant activity.^276^ For example, Au NPs conjugated with flagellin of *Pseudomonas aeruginosa* performed comparable titers of antiflagellin antibodies as flagellin formulated in Freund's adjuvant.^258^ In another study, Vetro et al. designed a glycoconjugate nanoparticle vaccine that modified Au NPs with pneumococcal capsular polysaccharide antigens, which were important component in current commercial vaccine and also essential in the infection of pneumococcus.^245^ A glucose derivative was added as inner component of Au NPs to increase the water solubility, and a T‐helper peptide OVA was loaded onto the Au NP as well. This glycoconjugate vaccine triggered potent and specific IgG Ab‐dependent immune response against *S. pneumonia* in mice. Many other researches have highlighted the enhanced anti‐infection outcome by using nanotechnology for delivery of antigen and/or adjuvants.^275^, ^277^, ^278^, ^279^ In the following part, we will put more emphasis on discussing the design and application of surface‐modified biomimetic nanoparticles as vaccine against bacterial infection. The nanoparticle platforms engineered with intrinsic toxin neutralizing ability and immune‐potentiating ability have superior properties compared to traditional methods on account of improved safety and more efficient toxin‐ or antigen‐specific elimination.

Pore‐forming toxins (PFTs), as an important virulence factor, could damage normal cells by forming pores in cell membranes. Numerous researches proved that elimination of PFTs had therapeutic effect on a variety of pathogens, including *Staphylococcus aureus*,^280^ *Escherichia coli*,^281^ *Listeria monocytogenes*,^282^ etc. On the basis of these findings, Hu et al. fused RBC membrane vesicles onto PLGA NPs to form a toxin nanosponge because RBC membrane could absorb and neutralize a wide range of PFTs with high affinity.^283^ In the meantime, the PLGA core guaranteed the stability of RBC membrane shell and extended the circulation time for prolonged elimination of toxins in the bloodstream. This PLGA NP‐based nanosponge remarkably neutralized staphylococcal α‐hemolysin, a model PFT, and diverted it away from intended target cells followed by metabolism through ingestion by hepatic macrophages. This method significantly reduced hepatotoxicity of α‐hemolysin and prolonged survival time of toxin‐challenged mice. Later, the broad‐spectrum detoxification ability of PLGA‐RBC membrane nanosponge was confirmed by rapid absorption and neutralization of four PFTs, that was, melittin, α‐hemolysin of methicillin‐resistant *S. aureus* (MRSA), listeriolysin O of *L. monocytogenes*, and streptolysin O of Group A *Streptococcus*, indicating that this nanoparticle‐based formulation provided potential therapy against various bacterial infections caused by PFTs.^284^ Despite excellent antibiotic efficiency, the coating of cell membrane onto PLGA NP might be complicated and membrane protein can be denatured during this process. To address this problem, He et al. fused RBC membrane with PEGylated artificial lipid membranes to facilitate extrusion through polycarbonate membrane and also protect the components on cell membranes, thereby the detoxification capacity of RBC membrane was maintained. The treatment could effectively protect mice from the damage induced by a model PFT α‐hemolysin.

Additionally, the PFT was also a routinely used candidate as vaccine to activate the immune system. However, the traditional protein denaturation method could reduce the vaccine potency and safety of toxoid. By using nanotechnology, the RBC membrane‐coated nanoparticle system was mixed with staphylococcal α‐hemolysin into nanotoxoid.^259^ The nanotoxoid neutralized the toxin's virulence without disrupting its structural integrity. Compared with heat‐treated toxin vaccination, the nanotoxoid exhibited improved immunogenicity and stronger protection to immunized mice. Later on, the vaccination efficacy of this RBS membrane and α‐hemolysin‐containing nanotoxoid was examined in a mouse model of MRSA.^260^ The antivirulence nanoparticle vaccination elicited strong and durable antigen response against α‐hemolysin, and alleviated both superficial damage and MRSA invasiveness. Different from antibiotics, nanotoxid does not directly target the elimination of single bacterium but aim to disturb the interaction between pathogen and host by neutralizing the harmful toxoids, so this method is less likely to develop resistance.^285^

**Figure 9:**
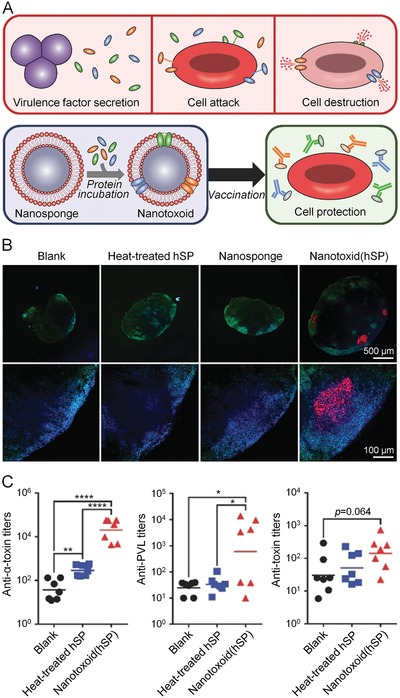
Antivirulence vaccination against bacteria infection via in situ capture of bacterial toxins. A) Fabrication process of nanotoxoid carrying pathogen‐specific virulence factors and its protection against toxic effect. B) Fluorescence imaging of B220 (green), IgD (blue), and GL‐7 (red) in draining LN at different magnifications. C) Multivalent Ab responses in mice vaccinated with different formulations. Reproduced with permission.^261^ Copyright 2017, John Wiley and Sons.

More recently, vaccination approaches that utilize more than one kind of virulence have been proposed and exerted enhanced performance, given that various toxins participate in the pathogenesis of bacteria infection. To this end, RBC membrane vesicles were coated onto PLGA NPs as nanosponges to entrap a wide range of virulence factors from hemolytic secreted protein (hSP) fraction of MRSA (**Figure** 9).^261^ The pathogen‐specific virulence factors delivered in nanotoxoid‐hSP elicited virulence‐specific antigens in the vaccinated mice, which effectively neutralized the toxicity caused by MRSA challenge. This phenomenon could attribute to the high concentration of germinal center marker GL‐7 in the draining LNs, representing promoted proliferation of B cells and accordingly stronger B cell immune response.

Apart from RBC membrane vesicles, bacterial outer membrane vesicles (OMVs) are another favorable option in the construction of biomimetic nanoparticle platforms. It was reported that OMVs were capable of inducing intense humoral responses in protection against bacterial infection mainly owing to the presence of plentiful immunogenic antigens and pathogen‐associated molecular patterns, which increased the secretion of proinflammatory cytokines and promoted the activation of DCs.^286^, ^287^ For example, Gao et al. collected OMVs secreted by *E. coli* and wrapped them onto the surface of Au NPs.^262^ After subcutaneous injection, the OMV‐NPs migrated to the nearby draining LN and rapidly induced the activation of DCs. Compared with treatment of OMVs, OMV‐NP vaccination generated stronger T cell and B cell immune response that protected mice from bacteria challenge, confirming a synergistic effect of bacteria membrane and Au NPs.

#### 2.2.4 Nanoparticle Vaccine for Other Infectious Diseases

The development of DNA vaccination is a critical improvement in medicine. However, in spite of low cost and rapid manufacture of DNA vaccination, its inferior stability and insufficient immunogenicity have limited the application in the prevention and treatment of various infectious diseases. Nanotechnology provides a new possibility in engineering DNA vaccine‐loaded nanoparticle platforms for controlled and targeted delivery to certain cells. Draz et al. reported a DNA vaccination against model hepatitis C virus by using electrically oscillating plasmonic Au NPs.^244^ The plasmonic Au NPs can be activated by certain electric pulsing to facilitate pore‐forming in nearby cell membrane and increase membrane permeability for DNA transfection. In this case, the DNA vaccine uptake by myocytes was significantly magnified after coadministration of free DNA plasmid and Au NPs in mice, allowing for more efficient expression of encoded genes. Moreover, in consideration of the low electric field needed in this process, cell destruction or lysis could be avoided.

The Ebola virus outbroke in West Africa in 2014 was a detrimental health threat with a mortality rate of more than 50%. In face of current obstacles in DNA vaccine, Yang et al. synthesized cationic PLGA–poly(L‐lysine)/γPGA (PLGA–PLL/γPGA) NPs coated with Ebola DNA vaccine on the surface and immunized mice by using microneedle patches made of water soluble poly(vinyl alcohol).^21^ The DNA vaccine delivery formulation achieved increased immunogenicity and stronger immune response.

Unlike DNA‐based vaccines, nonretroviral RNA vaccines are free from the risk of integration into patient's genome. It was reported that replicon mRNA could achieve sustained translation and amplification of encoded protein. Chahal et al. developed a modified dendrimer nanoparticle (MDNP) vaccine consisting of a cationic and ionizable dendrimer, a lipid‐PEG segment, and a self‐replicating antigenic RNA.^22^ This formulation elicited both Ab secretion and antigen‐specific CTL response to protect against lethal dose of pathogen. Interestingly, by encapsulating different RNAs encoding various antigens, the MDNP vaccine could be applied to prevent several virus challenges, such as H1N1 influenza, Ebola virus, *Toxoplasma gondii*, and Zika virus.^288^

## 3 Nanoparticles for Immunosuppression

In addition to the capability to improve proinflammatory immune response, nanoparticle platforms are also envisioned to promote immune tolerance against chronic or acute inflammations, autoimmune diseases, transplant rejection, and allergies. Contrary to cancer and infections that invade human body on account of insufficient immune reaction, these diseases result from inappropriate overreaction of immune system to self‐antigens, allogenic antigens in transplantation, or environmental factors.^5^ Given that immunostimulation of nanotechnology has gained much attention, monitoring immunosuppressive properties of nanomaterials is equally important in relieving immune‐mediated burdens. Immunosuppressive drugs, mostly small molecules, have shown improved therapeutic efficiency in recent years. However, long‐term treatment with immunosuppressant can lead to severe systemic toxicity or immunodeficiency.^289^ Many immunosuppressive agents, such as methotrexate, rapamycin, and dexamethasone, are hydrophobic drugs and have limited bioactivity. These agents are randomly and extensively distributed in the body after administration, thereby leading to severe side effects to off‐target tissues and causing damage to the liver, muscle, and gastrointestinal tract.^290^, ^291^ Anti‐inflammatory cytokines, such as IL‐4, have been widely studied in the treatment of various autoimmune diseases.^292^ However, their short half‐life determines high‐dose administration and inevitable systemic toxicity.^293^ Therapeutic delivery of microRNA (miRNA) for symptom control can be also challenging due to limited potency, low stability, and lack of targeting.^5^ Nanotechnology overcomes the current shortcomings of immunosuppressive agents through multiple aspects, such as providing protection against degradation, prolonging circulation, and facilitating immune cell‐targeting delivery.^294^ Nanoparticle itself can also be engineered into immunomodulatory component and nanoparticles delivering antigen‐MHC complex can expand antigen‐specific Tregs to control inflammation disorders. In this section, we will discuss the approaches of inducing immune tolerance against overactive immune response by regulating two essential cell types, macrophages and Tregs. The nanoparticle‐based immunomodulatory systems for immunosuppression were listed in **Table** 4.

**Table 4:** Nanoparticle‐based immunomodulatory systems for immunosuppression

	Nanoparticle	Payload	Targeting	Stimuli‐sensitivity	Reference
Modulation of macrophages	Nanoparticle self‐assembled from dextran sulfate‐5β‐cholanic acid	Methotrexate	Scavenger receptor on macrophages	–	45
	HA‐NP		CD44 and TLR4 on macrophages	–	295
	Dextran–dexamethasone prodrug		Lectin and scavenger receptor on macrophages	Esterase	44
	Au NP	IL‐4	–	–	296
	MSNP	IL‐4	–	–	297
	MTC cross‐linked with miRNA‐146b		Mannose receptor on BMDMs	–	298
	Silica nanoparticle conjugated with KGM	–	Mannose receptor on BMDMs	–	299
	RGD‐coated Au NP protected by a magnetic nanocage		–	–	300
Modulation of Tregs	Au NP	Hyperforin	–	–	301
	Au NP	Hexapeptide	–	–	302
	PLGA NP	Protein or peptide antigens and rapamycin	–	–	303
	PLA–PLGA NP	PLP and rapamycin	–	–	304
	Porous silicon nanoparticle	Rapamycin and OVA peptide	CD11c on DCs	–	36
	PLGA NP	Anti‐CD3 Ab	PNAd on HEV	–	42
	PLGA NP	Diabetogenic peptide	–	–	305
	Iron oxide nanoparticle	T1D‐relevant peptide‐MHC class I complexe	–	–	306
	Iron oxide nanoparticle	Diabetogenic antigen peptide‐MHC class II complex	–	–	307
	PLGA NP	IL‐2 and TGF‐β	Biotin on CD4+ T cells	–	308

### 3.1 Modulation of Macrophages

As discussed above, M1 macrophages as a typical APC perform antigen presentation and proinflammatory effect in antitumor therapy. On the contrary, M2 is a prohealing phenotype that assists in anti‐inflammation process and tissue repair by enhancing the secretion of IL‐10, TGF‐β, and VEGF.^309^ Transition of M1 and M2 phenotypes is associated with the initiation and progression of inflammatory diseases, infection, atherosclerosis, obesity, diabetes, asthma, and sepsis.^310^ Many nanoparticle platforms have been developed to reduce proinflammatory macrophages or regulate macrophage polarization for treatment of dysfunctional macrophage‐associated diseases.^44^, ^295^, ^311^, ^312^

Activated macrophages are reckoned to participate in the inflammation and pathogenesis of RA. Varieties of nanoparticle carriers have been adopted in targeted delivery of anti‐inflammatory drugs to inflamed sites through selective combination with molecules overexpressed on the surface of activated macrophages, such as folate receptor, scavenger receptor, and CD44 receptor.^39^, ^40^, ^41^ In a study by Heo et al., nanoparticle self‐assembled from dextran sulfate‐5β‐cholanic acid was designed for targeted delivery of antiarthritis drug methotrexate to collagen‐induced arthritis (CIA) mice based on the specific interaction between dextran sulfate and scavenger receptor.^45^ After intravenous injection, the nanoparticles could aggregate at the inflamed joints of CIA mice and the accumulation maximized at 12 h after injection. The targeted therapy gained much better anti‐inflammatory effect compared with free methotrexate, characterized by better clinical scores and lower paw thickness.

Macrophages are also important propagators in atherosclerotic plaques. In order to alleviate atherosclerosis‐associated inflammation, polymerized HA NPs were prepared for specific targeting through interacting with receptors, such as CD44 and TLR4 expressed on macrophages.^295^ The polymerized HA was associated with phagocytosis inhibition and anti‐inflammatory effects, contrary to angiogenesis and inflammation‐stimulatory ability of low‐molecular weight HA.^313^, ^314^ HA‐NPs showed higher internalization by proinflammatory macrophages than by anti‐inflammatory phenotype in vitro. Moreover, HA‐NPs treatment effectively decreased immune cell infiltration in a rabbit model of atherosclerosis.

Obesity increases the M1 macrophage infiltration in adipose tissue and promotes the secretion of pro‐inflammatory cytokines, such as TNF‐α and IL‐6, which lead to high risk of glucose intolerance and insulin resistance.^315^ Ma et al. synthesized a nanosized dextran–dexamethasone prodrug that was linked through an esterase‐sensitive ester bond.^44^ Dexamethasone is an anti‐inflammatory drug against M1 macrophage and has shown therapeutic effect in obesity and diabetic patients, while dextran can selectively bind to lectins and scavenger receptors on macrophages. It was found that, after regional peritoneal administration, the dextran–dexamethasone conjugate specifically accumulated at visceral adipose tissue, then entered macrophages via the receptor‐mediated pathway, and was finally cleaved by intracellular esterase. A single dose of dexamethasone prodrug administrated to obese mice achieved a trend of lower levels of TNF‐α, IL‐6, and monocyte chemoattractant protein‐1, a typical chemoattractants of macrophage, in adipose tissues. This targeted delivery of anti‐inflammatory drugs was a promising method to prevent the occurrence of obesity comorbidities, including type 2 diabetes, heart disease, and stroke.

In tissue injury or infection, acute M1 response is necessary to eliminate the invading pathogens, whereas uncontrolled and prolonged activation of M1 macrophage or imbalanced M1/M2 ratio can lead to tissue damage or bad regeneration.^296^ IL‐4 is a potent inflammation‐inhibitory cytokine that induces the transition of M1 macrophage to M2 state. It has been widely used in the treatment of autoimmune disease and chronic skin inflammation.^292^, ^316^ In order to overcome the drawbacks of IL‐4, such as short half‐life and off‐target side effects, Raimondo and Mooney conjugated IL‐4 onto Au NPs to treat ischemic skeletal muscle injury.^296^ IL‐4‐Au NP injection induced more evident skew toward M2 macrophage and more significant decrease of M1 type in comparison with bolus IL‐4 in a mouse model, leading to functional muscle improvement and muscle regeneration. In another study, MSNPs with 30 nm extra‐large pores were produced to deliver IL‐4 at a high loading content.^297^ Researchers found that IL‐4‐MSNPs were preferably engulfed by phagocytic cells, including macrophages, DCs, neutrophils, and monocytes, but did not arouse migration or proliferation of inflammatory cells. Most importantly, M2 macrophage polarization could be efficiently induced in vitro and in vivo without obvious ROS production, and the effect of M1 phenotype was also suppressed.

Inflammatory bowel disease (IBD) is featured by dysregulation of macrophages and impaired mucosal repair.^317^ In a study by Deng et al., the mannose‐modified trimethyl chitosan (MTC) was cross‐linked with miRNA‐146b via ionic interaction to produce nanoparticles for selective endocytosis by intestinal macrophages in treatment of ulcerative colitis.^298^ Previous study found the enhanced expression of miRNA‐146b inhibited the orientation to M1 macrophage and suppressed inflammation in an IL‐10‐dependent manner.^318^, ^319^ The in vitro examination suggested that bone marrow‐derived macrophages (BMDMs) treated with miRNA‐146b exhibited M2 phenotype and elevated IL‐10 expression, which stimulated epithelial cell proliferation. After oral administration of miRNA‐146b nanoparticles, the dextran sodium sulfate‐induced colitis mice showed drastic body weight recovery, and rapid restoration of colonic epithelial cells and gut barrier integrity. In another study, Gan et al. presented an interesting hypothesis that mannose receptor clustering on macrophages might lead to polarization to M2 phenotype.^299^ To this end, they conjugated konjac glucomannan (KGM), a ligand of mannose receptor, to silica nanoparticles with varied sizes. It was observed that KGM‐NPs of 30 nm in diameter could induce the formation of mannose receptor nanoclusters on BMDMs and increase the gene expression of arginase‐1, MRC, and IL‐10, indicating a transition into M2 type. Intracolonic administration of KGM‐NPs to 2,4,6‐trinitrobenzenesulfonic acid solution‐induced IBD mice notably alleviated mucosal inflammation and colitis symptoms, and largely prolonged survival time.

**Figure 10:**
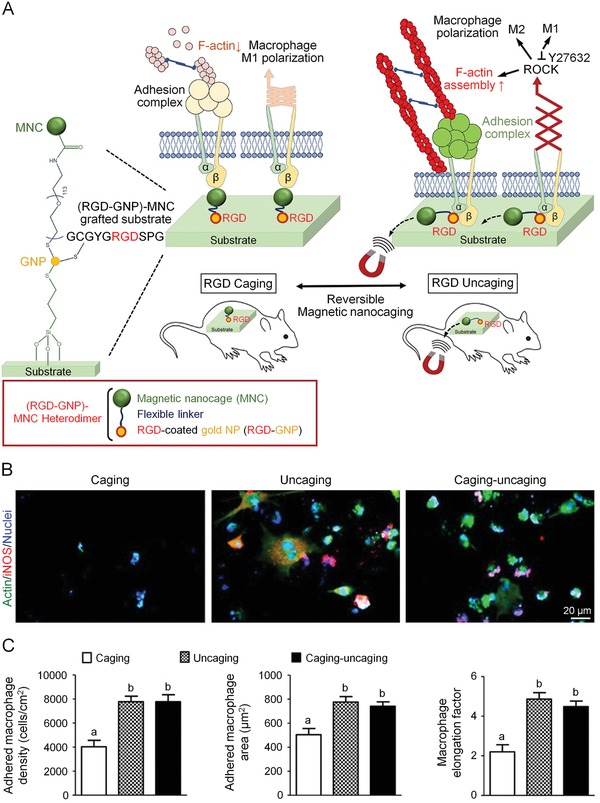
Reversible magnetic manipulation of RGD nanocaging controlled macrophage polarization. A) Fabrication of RGD‐Au NP‐magnetic nanocage heterodimer nanostructure, and manipulation of macrophage polarization via a magnetic field. B) Immunofluorescence micrographs of host macrophages staining actin (green), Arg‐1 (red), and nuclei (blue) following 24 h of RGD caging or uncaging. RGD caging–uncaging group received RGD caging for the initial 12 h followed by uncaging in the next 12 h. C) Quantification of cell density, area, and aspect ratio or macrophages after 24 h. Reproduced with permission.^300^ Copyright 2018, American Chemical Society.

Some versatile nanoparticles were designed with fascinating properties in order to realize selective delivery to cells of interest and smart control of macrophage polarization. For example, Lee et al. proposed that the inflammatory reaction to implanted materials in vivo could be controlled by presenting bioactive molecules on nanomaterials.^320^ They found that the light‐triggered activation of RGD (Arg‐Gly‐Asp) peptide on biomaterial implant could regulate macrophages adhesion in vivo. Based on this finding, Kang et al. further discussed whether RGD activation could influence the transition of the two macrophage phenotypes in vivo (**Figure** 10).^300^ In this study, RGD‐coated Au NP was conjugated to the substrate and protected by a magnetic nanocage to form the heterodimer nanostructure. The caging and uncaging of RGD could be remotely and reversibly controlled by a magnetic field. They discovered that RGD uncaging elicited a temporal recruitment of macrophages and pro‐regenerative M2 polarization, while in the meantime inhibited the skew to M1 type. The magnetic field allowed for deeper tissue penetration and better cytocompatibility than ultraviolet light, and conveniently manipulated RGD activation to reduce inflammation at the implanted material and promote tissue regeneration.

### 3.2 Modulation of Regulatory T cells

Tregs are another important participant in regulating peripheral immunological tolerance.^321^ These cells inhibit the function of other immune cells, including CD4+ and CD8+ T cells, DCs, and macrophages through various mechanisms.^322^ For example, Tregs inhibit T cells survival and proliferation by depleting IL‐2, and prevent APCs maturation and T cell activation by releasing immunosuppressive cytokines IL‐10 and TGF‐β.^323^, ^324^ Until now, immune‐modulatory methods that regulate function of Tregs have been used in the treatment of autoimmunity, chronic inflammation, and tissue regeneration. Nanotechnology drastically improves the anti‐inflammatory efficacy of encapsulated payload. Moreover, the intrinsic immunomodulatory capability of nanoparticles has also been explored to induce Tregs expansion and hinder the progression of diseases.

Multiple sclerosis (MS) occurs after aberrant activation of pathogenic T cells and dysfunction of Tregs. In one study, Nosratabadi et al. evaluated the anti‐MS function of hyperforin‐encapsulating Au NPs in an experimental autoimmune encephalomyelitis (EAE) model, a typical model for the study of MS.^301^ Free hyperforin, as a traditional anti‐inflammation medicine, caused expansion of Treg population in splenocytes, whereas this effect was surpassed by hyperforin‐Au NPs. This result could be explained by the efficient entrance of Au NP into various cell types with low cytotoxicity and high biocompatibility.^325^ Through the induction of Tregs, hyperforin‐Au NP treatment achieved improved disease clinical score of EAE and less infiltration of inflammatory cells.

Acute lung injury is a life‐threatening illness that features severe inflammation in the lung and excessive TLR activation.^326^ Xiong et al. developed a peptide‐Au NP hybrid that contained a potent TLR inhibitor hexapeptide on the surface to enhance stability and inhibit TLR2, TLR3, TLR4, and TLR5 signaling pathways after cell uptake.^302^ In a LPS‐induced acute lung injury model, intratracheal instillation of hexapeptide‐Au NPs exhibited evident inhibition toward TLR activation and significantly increased the population of Tregs in the lung. The latter promoted the apoptosis of neutrophils, leading to reduced inflammation infiltration.

Although immunomodulatory medicine is a conventional treatment method for various autoimmune diseases, they usually have broad immunosuppression, and long‐term usage can lead to the activation of potential pathogens and even the development of tumors.^327^, ^328^ To this end, antigen‐specific immunological tolerance is more beneficial in efficacy and safety concern. Immunosuppressants, such as rapamycin, can stimulate the generation of tolerogenic DCs featuring low expression level of costimulatory molecules and minimal production of proinflammatory cytokines. In this circumstance, the antigens presented to DCs do not enable the induction of effector T cells but contrarily promote the differentiation of antigen‐specific Tregs.^329^ However, free rapamycin therapy requires long‐term systemic treatment and tends to induce uncontrolled immunosuppression. By using nanotechnology, rapamycin and antigen could be simultaneously presented to APCs where rapamycin transiently acts on DCs to prevent the induction of systemic immunosuppression. For example, Maldonado et al. synthesized tolerogenic PLGA NPs containing peptide antigens and rapamycin to induce the antigen‐specific tolerance by inhibiting T cell activation and generating Tregs in multiple animal models, including EAE.^303^ In this study, the tolerogenic nanoparticles could inhibit the differentiation of B cells into Ab‐producing cells and attenuate preexisting anti‐OVA Abs even at the presence of potent adjuvants CpG and R848. More importantly, the immune tolerance could be maintained for as long as 111 days without systemic immunosuppression. Mice vaccinated with nanoparticles encapsulating myelin proteolipid protein peptide fragment (PLP) and rapamycin before induction of EAE showed evidently reduced severity of paralysis, and this tolerogenic nanoparticle therapy during the peak of disease thoroughly prevented EAE relapse. Therefore, the antigen‐specific immune tolerance could be a potential strategy for prevention and treatment of various autoimmune diseases. Later on, the therapeutic effect of PLA–PLGA NPs delivering PLP and rapamycin was verified in a model of relapsing EAE.^304^ Interestingly, the authors discovered that splenocytes from tolerogenic nanoparticle‐vaccine mice had a protective effect against antigen challenge in naive mice.

In some other cases, nanomaterials are modified with extra properties for targeting delivery and improved efficacy of the payload. For example, Stead et al. proposed a nanoparticle‐based method for generation of Tregs in vivo with the purpose of inhibiting chronic graft rejection after organ transplantation.^36^ Specifically, rapamycin‐ and OVA peptide‐loaded porous silicon nanoparticles were endowed with murine DC‐targeting ability by surface coupling with CD11c Ab. The DC‐targeting nanoparticles were predominantly internalized by DCs in the spleen and peripheral blood. Injection of CD11c‐NP in OVA sensitized mice strongly triggered Tregs proliferation compared to control group.

**Figure 11:**
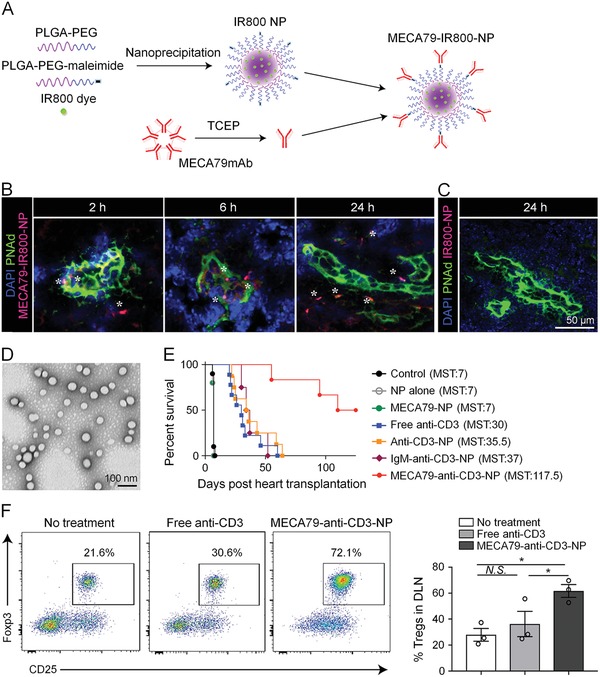
Targeted delivery of anti‐CD3 Ab to LN to suppress transplant rejection. A) Schematic illustration of IR800‐NP synthesis process and conjugation with MECA79 monoclonal Ab. B,C) Detection of B) MECA79‐IR800‐NPs and C) nontargeted IR800‐NPs trafficking in the draining LN by staining IR800 (red), PNAd (green), and nuclei (blue). D) TEM of IR800‐NPs. E) Survival of C57BL/6 recipients receiving different treatments after transplantation with BALB/C hearts. F) Proportion of Tregs in draining LN of recipient mice by flow cytometric analysis and representative flow plots. Reproduced from with permission.^42^ Copyright 2018, American Society for Clinical Investigation.

In another study, a LN‐targeting PLGA NP was used as a carrier for anti‐CD3 monoclonal Ab to improve the transplant survival rate of cardiac allograft (**Figure** 11).^42^ The anti‐CD3 Abs have wide applications in organ transplantation by expanding Treg population and inhibiting pathogenic T cells.^330^, ^331^ MECA79 monoclonal Ab was coated onto nanoparticles for selective recognition of peripheral node addressin (PNAd) on the high endothelial venule (HEV) of LN. MECA79‐conjugated nanoparticle could traffic to HEV as early as 2 h after intravenous administration and migrate to adjacent LNs at 24 h. The MECA79‐anti‐CD3‐NP increased the Treg ratio in the draining LN and remarkably prolonged the survival time in a murine heart transplant model in comparison with unconjugated nanoparticle.

Type 1 diabetes (T1D) is a typical autoimmune disease characterized by overactive CD4+ and CD8+ T cells due to anergic status of peripheral immune tolerance. In one study, diabetogenic peptides were either conjugated to or encapsulated by PLGA NPs for the treatment of T1D.^305^ Antigen‐PLGA NP therapy effectively enhanced the uptake by APCs, and upregulated the level of anti‐inflammatory cytokines IL‐10 and TGF‐β. Most importantly, this process evidently induced the systemic expansion of peptide‐specific Tregs, along with high expression of CTLA‐4 and PD‐1, which directly restored tolerance in both CD4+ and CD8+ T cells. It was reported that peptides bound to MHC molecules were more tolerogenic than those used alone.^332^ Moreover, the number of antigen‐specific Tregs expanded by peptide‐MHC NPs in vivo can be hundreds of times larger than that of Tregs expanded in vitro to produce clinical response in patients with inflammatory disorders.^333^, ^334^ For this reason, Tsai et al. coated iron oxide nanoparticles with T1D‐relevant peptide‐MHC class I complex as a tolerogenic vaccine.^306^ Systemic administration of the nanoparticle vaccine expanded autoregulatory CD8+ T cells and severely blunted the progression of T1D. Later, Clemente‐Casares et al. treated T1D mice with iron oxide nanoparticles coated with diabetogenic antigen peptide‐MHC class II complex.^307^ The treatment induced the differentiation of autoreactive T cells into antigen‐specific CD4+ Tregs. They developed a series of peptide‐MHCII‐based nanovaccines that had biological effects not only on diabetic mice but also on other autoimmune diseases like EAE and arthritis.

In order to define the engineering principles for the optimal design of peptide‐MHC nanoformulations, the same research group prepared iron oxide nanoparticles with stronger peptide‐MHC binding capacity.^335^ In this study, the researchers found that the density of peptide‐MHC on the nanoparticles might have an effect on the formation of Tregs while the dose controlled the expansion of Tregs. It also reported that the peptide‐MHC NPs prolonged the ligation with TCR on cognate T cells and therefore promoted Tregs conversion. More importantly, the optimal nanoformulation did not promote cytokine or chemokine secretion, and no off‐target toxicity was detected in zebrafish embryos.

As is well known, the survival and proliferation of Tregs is largely determined by the expression of IL‐2 and TGF‐β. Previous studies reported that these two cytokines were able to induce the generation of Tregs from naive CD4+ cells ex vivo.^336^, ^337^ To this end, a CD4‐targeted nanoparticle was formed based on the specific conjugation between avidin‐coated PLGA NP and biotin‐anti‐CD4 for targeted delivery of IL‐2 and TGF‐β.^308^ After intraperitoneal injection of nanoparticles in mice, it was found that the percentage of Treg in CD4+ T cell compartment was significantly elevated within the mesenteric LNs and spleen. In addition, nanoparticles demonstrated a higher suppression toward the proliferation of CD4+ T cells compared with soluble cytokines. Meanwhile, the conjugation with Tregs posed no threat to their function.

## 4 Conclusion and Future Perspectives

Nanomaterial‐based immunotherapy has gone through rapid development and exhibited considerable potential during the past few decades. With constantly improved fabrication methods and design strategies, nanotechnology has been well harnessed in the control and prevention of many diseases through immune regulation. As discussed in this Review, emerging evidence has highlighted the excellent outcomes in tumor therapy by activating APCs and T cells, regulating Tregs, TAMs, and MDSCs in immunosuppressive TME, and synergizing with chemotherapy, phototherapy, and radiotherapy. In the prevention and elimination of infectious viruses and bacteria, nanoparticle‐based vaccine allows higher uptake by APCs and induces improved T cell and B cell responses. In addition, functionalized nanoparticles can regulate the polarization of macrophages and the population of immunosuppressive Tregs to regain immune tolerance in the treatment of inflammatory diseases, transplant rejection, allergies, and autoimmune diseases, including RA, diabetes, MS, and so forth.

Codelivery of tumor antigens and adjuvants in nanosized carriers enhances the efficacies of cancer vaccines. More recently, a new trend in cancer immunotherapy involves the recognition of tumor neoantigens, which derived from patient‐specific cancer mutations and can be identified as “nonself” by immune system.^338^ Studies employing exogenous neoantigen in tumor vaccine showed strong immune response against cancer. As discussed above, the HDL‐based nanodiscs encapsulated with neoantigen and adjuvant CpG significantly promoted antigen uptake by APCs and T cell immune response.^135^ In another study, the tumor neoantigen‐coding mRNA delivered by liposomes was efficiently captured by lymphoid‐resident DCs and induced strong effector T cell activation and memory T cell response. This strategy triggered IFN‐α expression and tumor neoantigen‐specific T cell response in three melanoma patients.^339^ Although tumor neoantigen can be an ideal candidate for personalized cancer immunotherapy, they are rare in some cancer types with low mutations, and thus a combination with radiotherapy or chemotherapy is preferred to increase the mutation load as well as tumor neoantigens. In addition, the process to identify and synthesize neoantigen peptides is time‐consuming, and new technique and method are urgently needed to shorten this period of time.^180^

More recently, Song et al. pointed out the importance of pattern recognition receptors (PRRs) expressed in innate immune cells and the potent ability of PRR agonists as adjuvants in activating innate immune system and instructing adaptive immune response.^340^ The PRR agonist can be delivered with tumor antigens by nanoplatform as nanovaccines, which achieved successful tumor inhibition in animal models. The “in situ vaccine” that directly delivers PRR agonist into tumor tissues is able to strengthen the antitumor immune response and alleviate the side effects. Nanomateirals for local injection and controlled drug release are of considerable interest, and are worth deep researches in anti‐inflammatory applications.

Combinatorial treatment that synergizes systemic immunoreaction and external therapies remarkably improves treatment outcomes and in the meantime alleviates adverse effects. ICD inducers, including chemotherapy, phototherapy, and radiotherapy combined with immune modulatory agents have evidently optimized tumor management. To date, researchers have found that manipulation of more than one ICD inducer in immunotherapeutic nanoplatform could achieve thorough tumor ablation. For example, during phototherapy, a part of cancer cells might escape the immune system and cause metastasis. Systemic administration of chemotherapeutics and immune stimulatory agents will overcome this limitation.^219^ Additionally, more investigations should be made on immune process and dynamic change of TME in order to tailor multiple‐therapy regimen to individual patient, which is also helpful to work out a specific therapeutic schedule includes precise timing of each treatment and optimal dosages. For example, a phase III study (NCT00409188) revealed that tecemotide vaccination concurrent with chemoradiatherapy could prolong the overall survival of NSCLC patients from 20.8 to 29.4 months, while sequential chemoradiatherapy administration did not realize any improvement, indicating that the timing of combinatory therapy is an influential factor.^69^

Nanotechnology has been offering great opportunities for the prevention and treatment of infectious diseases. Codelivery of antigens and adjuvants markedly enhances the immunogenicity of microbe components and shows higher efficiency than conventional vaccines that use whole microbes. According to present findings, polymer‐based DNA vaccination holds great promise for combating infectious diseases. Well engineered nanosystems are in need to deliver plasmid DNA in a safe and efficient way, which requires rapid LN trafficking and effective transfection to target immune cells. To date, one major public concern is high antibiotic toxicity and drug resistance after antibiotic treatment. Nanomaterials have emerged as a new antibacterial weapon to complement antibiotics in defending against various microbe infections, including antibiotic resistant bacteria. It has been reported that nanomaterials exert lethality through two pathways, i.e., disruption of cell membranes and production of ROS.^341^ Compared with antibiotics, nanomaterials offer enhanced antimicrobial activity with lower toxicity and avoid causing drug resistance. Beyth et al. reviewed that inorganic nanoparticles consisting of silver, gold, titanium dioxide, zinc oxide, iron oxide, or copper oxide, and organic nanoparticles made of poly(ε‐lysine), polysiloxanes, polyamines, chitosan, or triclosan have shown outstanding antibacterial and antiviral activity.^342^ As a promising approach for infection control, the exact toxicological mechanisms of these nanomaterials and hidden hazard to host cells still need further exploration.

The rapid advancement of biomimetic nanoparticles may provide a new idea for patient‐specific vaccine. Due to the great similarity of OMVs with bacterial membranes, OMVs provide both a wide variety of antigens and immune stimuli, including LPS, flagellin, and monophosphoryl lipid A.^275^ OMVs are also genetically engineered to express mutant antigens for more specific and broad‐spectrum immune protection.^343^, ^344^ In combination with nanotechnology, the stability and immune efficacy of OMVs are expected to be largely improved. With functionalizations, such as targeting moieties and stimuli responsiveness, the bacterial membrane‐coated nanoparticles can be expected to perform controlled and selective immune activation.

Similar to immunostimulation, immunosuppressive function of nanomaterial is largely dependent on physicochemical properties of nanoparticles. Current immunosuppressive therapies cannot fully exclude the risk of causing immunodeficiency, which is accompanied with myelosuppression, excessive sensitivity to opportunistic pathogens, and increased toxicity of nanomaterials. Future studies should put more emphasis on the mechanisms of nanosystem‐regulated immunosuppression as well as the precise control over immune suppression effect by identifying the optimal dosage and administration route. Moreover, considering different immune cells or cell lineages might have very different sensitivities toward a certain nanoparticle type, it is necessary to carefully evaluate the immunological response to nanoparticles by in vitro cell experiments in the first place. Additionally, in order to drive effective immune tolerance, antigens and immunosuppressants must be drained to adjacent LNs and delivered to the right immune cells, which presents a high requirement for targeting ability of nanoplatform.

Antigen selection is another major obstacle in vaccine formulation due to the varying candidate antigens during disease progression, not to mention the huge differences in antigen epitopes among patients. Acquiring protein or peptide antigens from cell membranes or cell lysates can be a possible way, while due to the complexity of antigen mixture, the concentration of individual antigen is limited and may not be enough to yield immune tolerance. Therefore, it still needs further exploration to search more appropriate antigen epitopes for enhanced therapeutic effect.

Although a number of nanoparticle‐based immunotherapies have entered clinical stage in the treatment of cancers and the prevention of infectious diseases (Table 1), the clinical translation of many other immunomodulatory nanosystems is still a major challenge. It requires a great deal of efforts and is largely dependent on many important factors. Safety is the top consideration for application of immunomodulatory nanomaterials. As nanoparticles interact closely with immune system, it requires a standard method to assess their possible immunotoxicity. For example, nanoparticles binding the serum proteins can form a protein corona around the surface. These particles could be recognized by immune system as nonself and induce autoimmunity. Nanomaterial might also be associated with allergic sensitization and hypersensitivity. Other toxicities, such as difficult clearance from human body and generation of ROS, should also be taken into consideration before clinical application. In addition, some nanoparticles may alter cell morphology and cytoskeleton, which can lead to disruption of intracellular signaling pathway. Therefore, the local and systemic toxicity of nanosystems toward normal organs and tissues should be carefully researched and evaluated. In view of the intrinsic adjuvant features of some nanoparticles, their immunogenicity is better to be properly controlled to avoid excessive immune response. At present, the nature‐derived nanomaterials have attracted increasing interest on safety concern, and new synthetic materials with excellent biocompatibility are yet to be discovered. In addition, it requires more precise and detailed investigation on the interaction between nanoparticles and the relevant biological components in systemic environment at different time points after administration. A comprehensive understanding of dynamic process of nanosystem and its function toward immune cells is useful for selecting the most suitable platform for a specific situation and treatment purpose.

A number of nanotechnology‐based vaccines require efficient cross‐presentation of antigens. Fabrication of pH‐sensitive nanodelivery platform for endosomal escape and establishment of aAPCs for direct T cell activation are both reasonable approaches as discussed above. Nanoparticles responding to other features, such as high oxidation in endosomal environment, can also be used as an alternative approach.^345^ In addition, cell‐penetrating peptides are able to promote the release of cargo trapped in endosomes into cytosol.^346^ Multiple mechanisms are involved in this endocytic pathway, such as macropinocytosis, clathrin‐mediated endocytosis, and caveolae/lipid raft‐mediated endocytosis. The usage of cell‐penetrating peptides has been proved effective in delivering a variety of cargoes, including proteins, small molecule therapeutics, and nucleic acids.^347^

It is reckoned that nanoparticles taken by phagocytic cells in mononuclear phagocyte system should be avoided to increase the drug accumulation at the diseased site. However, this process turns out to be beneficial for nanoparticles targeting macrophages or DCs.^348^ Therefore, for different applications, the characteristics of nanoparticles, such as size, surface chemistry, and surface charge, can be tuned to determine homing to or avoiding the endocytosis by the phagocytes.^349^, ^350^ Nanomedicines targeting immune cells in LNs should be designed differently from those targeting immune cells in TME. It is a bigger challenge to deliver nanomedicines to specific immune cell subsets.^351^ Nanomaterials that are functionalized with efficient targeting ligands and/or stimuli‐responsive ability allow targeted delivery and controlled responsive release of antigens, adjuvants, or immunomodulators. To achieve precise transportation of cargoes to specific tissues or cell populations, nanoparticles conjugated with an exclusive ligand or multiple targeting ligands can hopefully enhance the specificity toward target cells. For example, by using “Ab microarray screening,” Yu et al. found that liposome surface coupled with anti‐CD37 and anti‐CD19/anti‐CD20 could target to leukemia cell lines and B chronic lymphocytic leukemia patient cells with higher delivery efficiency and stronger ability to induce apoptosis.^352^

Compared with general functions of nanoparticles as delivery vehicle, including efficient and targeted delivery, controlled cargo release, and alleviated side effects, much less attention has been focused on how the very nature attributes of nanoparticles impact immune system and drug delivery, such as size, shape, elasticity, surface charge, morphology, and adjuvant function. Physical properties of nanoparticles also play an essential part in increasing local distribution and aggregation. For example, it was found that nanoparticles ranging from 10 to 100 nm in diameter had the most efficient drainage to LNs, whereas larger ones would be trapped within extracellular matrix and smaller ones could freely penetrate through LNs, which reduced the chances of being taken up by APCs.^353^ Additionally, shape also matters in engineering aAPCs to activate CD8+ T cells. Sunshine et al. found that ellipsoidal aAPCs showed higher activity than spherical ones, and this trend could be further enhanced by increasing the aspect ratios when antigen dose and particle volume were controlled equivalent.^354^ It can be explained by that long axis of ellipsoidal aAPCs increased the interaction with CD8+ T cells to facilitate their activation. Therefore, physicochemical parameters of nanoparticles should be taken into consideration when choosing appropriate targeted delivery vehicles.

Overall, exploring novel immunotherapies with efficient manipulation of nanosystem is an appealing and promising research field. Although we are now facing many obstacles, we can expect profound clinical improvement and benefit to human health in the near future.

## Conflict of Interest

The authors declare no conflict of interest.

## References

[1] G. P. Dunn , A. T. Bruce , H. Ikeda , L. J. Old , R. D. Schreiber , Nat. Immunol. 2002, 3, 991. 12407406 10.1038/ni1102-991

[2] B. B. Finlay , G. McFadden , Cell 2006, 124, 767. 16497587 10.1016/j.cell.2006.01.034

[3] D. F. Quail , J. A. Joyce , Nat. Med. 2013, 19, 1423. 24202395 10.1038/nm.3394 PMC3954707

[4] R. M. Pearson , L. M. Casey , K. R. Hughes , S. D. Miller , L. D. Shea , Adv. Drug Delivery Rev. 2017, 114, 240. 10.1016/j.addr.2017.04.005 PMC5582017 28414079

[5] A. Ilinskaya , M. Dobrovolskaia , Br. J. Pharmacol. 2014, 171, 3988. 24724793 10.1111/bph.12722 PMC4243973

[6] J. R. Gordon , Y. Ma , L. Churchman , S. A. Gordon , W. Dawicki , Front. Immunol. 2014, 5, 7. 24550907 10.3389/fimmu.2014.00007 PMC3907717

[7] Y. Shen , T. Hao , S. Ou , C. Hu , L. Chen , MedChemComm 2018, 9, 226. 30108916 10.1039/c7md00158d PMC6083789

[8] T. Tazaki , K. Tabata , A. Ainai , Y. Ohara , S. Kobayashi , T. Ninomiya , Y. Orba , H. Mitomo , T. Nakano , H. Hasegawa , K. Ijiro , H. Sawa , T. Suzuki , K. Niikura , RSC Adv. 2018, 8, 16527. 10.1039/c8ra01690a PMC9080258 35540526

[9] H. Kim , L. Niu , P. Larson , T. A. Kucaba , K. A. Murphy , B. R. James , D. M. Ferguson , T. S. Griffith , J. Panyam , Biomaterials 2018, 164, 38. 29482062 10.1016/j.biomaterials.2018.02.034

[10] D. Li , H. Sun , J. Ding , Z. Tang , Y. Zhang , W. Xu , X. Zhuang , X. Chen , Acta Biomater. 2013, 9, 8875. 23831719 10.1016/j.actbio.2013.06.041

[11] W. Xu , J. Ding , L. Li , C. Xiao , X. Zhuang , X. Chen , Chem. Commun. 2015, 51, 6812. 10.1039/c5cc01371b 25787235

[12] D. Li , W. Xu , P. Li , J. Ding , Z. Cheng , L. Chen , L. Yan , X. Chen , Mol. Pharmaceutics 2016, 13, 4231. 10.1021/acs.molpharmaceut.6b00747 PMC5464407 27784155

[13] J. Ding , C. Xiao , Y. Li , Y. Cheng , N. Wang , C. He , X. Zhuang , X. Zhu , X. Chen , J. Controlled Release 2013, 169, 193. 10.1016/j.jconrel.2012.12.006 23247039

[14] J. Chen , J. Ding , W. Xu , T. Sun , H. Xiao , X. Zhuang , X. Chen , Nano Lett. 2017, 17, 4526. 28644032 10.1021/acs.nanolett.7b02129

[15] W. Xu , J. Ding , X. Chen , Biomacromolecules 2017, 18, 3291. 28877434 10.1021/acs.biomac.7b00950

[16] Y. Zhang , L. Cai , D. Li , Y. H. Lao , D. Liu , M. Li , J. Ding , X. Chen , Nano Res. 2018, 11, 4806.

[17] S. Gao , G. Tang , D. Hua , R. Xiong , J. Han , S. Jiang , Q. Zhang , C. Huang , J. Mater. Chem. B 2019, 7, 709. 10.1039/c8tb02491j 32254845

[18] S. Musetti , L. Huang , ACS Nano 2018, 12, 11740. 30508378 10.1021/acsnano.8b05893

[19] E. R. Steenblock , T. M. Fahmy , Mol. Ther. 2008, 16, 765. 18334990 10.1038/mt.2008.11

[20] Y. C. Kim , J. H. Park , M. R. Prausnitz , Adv. Drug Delivery Rev. 2012, 64, 1547. 10.1016/j.addr.2012.04.005 PMC3419303 22575858

[21] H. W. Yang , L. Ye , X. D. Guo , C. Yang , R. W. Compans , M. R. Prausnitz , Adv. Healthcare Mater. 2017, 6, 1600750. 10.1002/adhm.201600750 28075069

[22] J. S. Chahal , O. F. Khan , C. L. Cooper , J. S. McPartlan , J. K. Tsosie , L. D. Tilley , S. M. Sidik , S. Lourido , R. Langer , S. Bavari , H. L. Ploegh , D. G. Anderson , Proc. Natl. Acad. Sci. USA 2016, 113, E4133. 27382155 10.1073/pnas.1600299113 PMC4961123

[23] S. Li , X. Feng , J. Wang , L. He , C. Wang , J. Ding , X. Chen , Nano Res. 2018, 11, 5769.

[24] H. Xiao , L. Yan , E. M. Dempsey , W. Song , R. Qi , W. Li , Y. Huang , X. Jing , D. Zhou , J. Ding , X. Chen , Prog. Polym. Sci. 2018, 87, 70.

[25] Y. Wang , Z. Jiang , W. Xu , Y. Yang , X. Zhuang , J. Ding , X. Chen , ACS Appl. Mater. Interfaces 2019, 11, 8725. 30785721 10.1021/acsami.9b01872

[26] Y. Fan , R. Kuai , Y. Xup , L. J. Ochyl , D. J. Irvine , J. J. Moon , Nano Lett. 2017, 17, 7387. 29144754 10.1021/acs.nanolett.7b03218 PMC5821496

[27] X. Song , J. Xu , C. Liang , Y. Chao , Q. Jin , C. Wang , M. Chen , Z. Liu , Nano Lett. 2018, 18, 6360. 30247918 10.1021/acs.nanolett.8b02720

[28] L. He , W. Xu , X. Wang , C. Wang , J. Ding , X. Chen , Biomater. Sci. 2018, 6, 1433. 29620095 10.1039/c8bm00190a

[29] J. Wang , W. Xu , S. Li , H. Qiu , Z. Li , C. Wang , X. Wang , J. Ding , J. Biomed. Nanotechnol. 2018, 14, 2102. 30305217 10.1166/jbn.2018.2624

[30] J. Chen , J. Ding , Y. Zhang , C. Xiao , X. Zhuang , X. Chen , Polym. Chem. 2015, 6, 397.

[31] H. Guo , F. Li , W. Xu , J. Chen , Y. Hou , C. Wang , J. Ding , X. Chen , Adv. Sci. 2018, 5, 1800004. 10.1002/advs.201800004 PMC6010003 29938183

[32] Y. Zhang , F. Wang , M. Li , Z. Yu , R. Qi , J. Ding , Z. Zhang , X. Chen , Adv. Sci. 2018, 5, 1700821. 10.1002/advs.201700821 PMC5980114 29876208

[33] L. Luo , C. Zhu , H. Yin , M. Jiang , J. Zhang , B. Qin , Z. Luo , X. Yuan , J. Yang , W. Li , Y. Du , J. You , ACS Nano 2018, 12, 7647. 30020768 10.1021/acsnano.8b00204

[34] J. Nam , S. Son , L. J. Ochyl , R. Kuai , A. Schwendeman , J. J. Moon , Nat. Commun. 2018, 9, 1074. 29540781 10.1038/s41467-018-03473-9 PMC5852008

[35] C. Wang , Y. Ye , Q. Hu , A. Bellotti , Z. Gu , Adv. Mater. 2017, 29, 1606036. 10.1002/adma.201606036 28556553

[36] S. O. Stead , S. Kireta , S. J. P. McInnes , F. D. Kette , K. N. Sivanathan , J. Kim , E. J. Cueto‐Diaz , F. Cunin , J. O. Durand , C. J. Drogemuller , R. P. Carroll , N. H. Voelcker , P. T. Coates , ACS Nano 2018, 12, 6637. 29979572 10.1021/acsnano.8b01625

[37] R. Yang , J. Xu , L. Xu , X. Sun , Q. Chen , Y. Zhao , R. Peng , Z. Liu , ACS Nano 2018, 12, 5121. 29771487 10.1021/acsnano.7b09041

[38] G. Shi , C. Zhang , R. Xu , J. Niu , H. Song , X. Zhang , W. Wang , Y. Wang , C. Li , X. Wei , D. Kong , Biomaterials 2017, 113, 191. 27816821 10.1016/j.biomaterials.2016.10.047

[39] R. Heo , J. S. Park , H. J. Jang , S. H. Kim , J. M. Shin , Y. D. Suh , J. H. Jeong , D. G. Jo , J. H. Park , J. Controlled Release 2014, 192, 295. 10.1016/j.jconrel.2014.07.057 25109660

[40] M. Yang , J. Ding , Y. Zhang , F. Chang , J. Wang , Z. Gao , X. Zhuang , X. Chen , J. Mater. Chem. B 2016, 4, 2102. 10.1039/c5tb02479j 32263177

[41] M. Yang , J. Ding , X. Feng , F. Chang , Y. Wang , Z. Gao , X. Zhuang , X. Chen , Theranostics 2017, 7, 97. 28042319 10.7150/thno.16844 PMC5196888

[42] B. Bahmani , M. Uehara , L. Jiang , F. Ordikhani , N. Banouni , T. Ichimura , Z. Solhjou , G. J. Furtmuller , G. Brandacher , D. Alvarez , U. H. von Andrian , K. Uchimura , Q. Xu , I. Vohra , O. A. Yilmam , Y. Haik , J. Azzi , V. Kasinath , J. S. Bromberg , M. M. McGrath , R. Abdi , J. Clin. Invest. 2018, 128, 4770. 30277476 10.1172/JCI120923 PMC6205374

[43] W. Ou , R. K. Thapa , L. Jiang , Z. C. Soe , M. Gautam , J. H. Chang , J. H. Jeong , S. K. Ku , H. G. Choi , C. S. Yong , J. O. Kim , J. Controlled Release 2018, 281, 84. 10.1016/j.jconrel.2018.05.018 29777794

[44] L. Ma , T. W. Liu , M. A. Wallig , I. T. Dobrucki , L. W. Dobrucki , E. R. Nelson , K. S. Swanson , A. M. Smith , ACS Nano 2016, 10, 6952. 27281538 10.1021/acsnano.6b02878

[45] R. Heo , D. G. You , W. Um , K. Y. Choi , S. Jeon , J. S. Park , Y. Choi , S. Kwon , K. Kim , I. C. Kwon , D. G. Jo , Y. M. Kang , J. H. Park , Biomaterials 2017, 131, 15. 28371624 10.1016/j.biomaterials.2017.03.044

[46] C. Tsopelas , R. Sutton , J. Nucl. Med. 2002, 43, 1377. 12368377

[47] J. Chen , J. Ding , Y. Wang , J. Cheng , S. Ji , X. Zhuang , X. Chen , Adv. Mater. 2017, 29, 1701170. 10.1002/adma.201701170 28632302

[48] J. Ding , W. Xu , Y. Zhang , D. Sun , C. Xiao , D. Liu , X. Zhu , X. Chen , J. Controlled Release 2013, 172, 444. 10.1016/j.jconrel.2013.05.029 23742879

[49] Z. Jiang , J. Chen , L. Cui , X. Zhuang , J. Ding , X. Chen , Small Methods 2018, 2, 1700307.

[50] Z. Jiang , Y. Liu , X. Feng , J. Ding , J. Funct. Polym. 2019, 32, 13.

[51] J. Ding , F. Shi , D. Li , L. Chen , X. Zhuang , X. Chen , Biomater. Sci. 2013, 1, 633. 10.1039/c3bm60024f 32481836

[52] D. Li , J. Han , J. Ding , L. Chen , X. Chen , Carbohydr. Polym. 2017, 161, 33. 28189244 10.1016/j.carbpol.2016.12.070

[53] X. Feng , D. Li , J. Han , X. Zhuang , J. Ding , Mater. Sci. Eng., C 2017, 76, 1121. 10.1016/j.msec.2017.03.201 28482476

[54] C. Zhang , G. Shi , J. Zhang , H. Song , J. Niu , S. Shi , P. Huang , Y. Wang , W. Wang , C. Li , D. Kong , J. Controlled Release 2017, 256, 170. 10.1016/j.jconrel.2017.04.020 28414151

[55] J. Ding , J. Chen , D. Li , C. Xiao , J. Zhang , C. He , X. Zhuang , X. Chen , J. Mater. Chem. B 2013, 1, 69. 10.1039/c2tb00063f 32260614

[56] K. Cheng , Y. Ding , Y. Zhao , S. Ye , X. Zhao , Y. Zhang , T. Ji , H. Wu , B. Wang , G. J. Anderson , L. Ren , G. Nie , Nano Lett. 2018, 18, 3250. 29683683 10.1021/acs.nanolett.8b01071

[57] L. Guo , D. D. Yan , D. Yang , Y. Li , X. Wang , O. Zalewski , B. Yan , W. Lu , ACS Nano 2014, 8, 5670. 24801008 10.1021/nn5002112 PMC4072412

[58] Q. Chen , L. Xu , C. Liang , C. Wang , R. Peng , Z. Liu , Nat. Commun. 2016, 7, 13193. 27767031 10.1038/ncomms13193 PMC5078754

[59] D. Li , G. Zhang , W. Xu , J. Wang , Y. Wang , L. Qiu , J. Ding , X. Yang , Theranostics 2017, 7, 4029. 29109796 10.7150/thno.19538 PMC5667423

[60] L. Galluzzi , J. M. Bravo‐San Pedro , G. Kroemer , Nat. Cell Biol. 2014, 16, 728. 25082195 10.1038/ncb3005

[61] R. A. Madan , B. Turkbey , L. M. Lepone , R. N. Donahue , I. Grenga , S. Borofsky , J. Clin. Oncol. 2017, 35, 30.

[62] E. Rossmann , A. Österborg , E. Löfvenberg , A. Choudhury , U. Forssmann , A. von Heydebreck , A. Schröder , H. Mellstedt , Hum. Vaccines Immunother. 2014, 10, 3394. 10.4161/hv.29918 PMC4514088 25483677

[63] S. S. Ramalingam , P. Mitchell , J. F. Vansteenkiste , J. Debus , W. J. Curran , M. A. Socinski , C. Heiwig , M. H. Falk , C. A. Butts , J. Clin. Oncol. 2014, 32, TPS7608.

[64] N. Katakami , T. Hida , H. Nokihara , F. Imamura , H. Sakai , S. Atagi , M. Nishio , T. Kashii , M. Satouchi , C. Helwig , M. Watanabe , T. Tamura , Lung Cancer 2017, 105, 23. 28236981 10.1016/j.lungcan.2017.01.007

[65] Y. L. Wu , K. Park , R. A. Soo , Y. Sun , K. Tyroller , D. Wages , G. Ely , J. C. H. Yang , T. Mok , BMC Cancer 2011, 11, 430. 21982342 10.1186/1471-2407-11-430 PMC3203100

[66] C. Butts , R. N. Murray , C. J. Smith , P. M. Ellis , K. Jasas , A. Maksymiuk , G. Goss , G. Ely , F. Beier , D. Soulières , Clin. Lung Cancer 2010, 11, 391. 21071331 10.3816/CLC.2010.n.101

[67] C. Butts , A. Maksymiuk , G. Goss , D. Soulieres , E. Marshall , Y. Cormier , P. M. Ellis , A. Price , R. Sawhney , F. Beier , M. Falk , N. Murray , J. Cancer Res. Clin. Oncol. 2011, 137, 1337. 21744082 10.1007/s00432-011-1003-3 PMC11828286

[68] M. DeGregorio , L. Soe , M. Wolf , J. Thorac. Dis. 2014, 6, 571. 24976972 10.3978/j.issn.2072-1439.2014.05.15 PMC4073360

[69] P. Mitchell , N. Thatcher , M. A. Socinski , E. Wasilewska‐Tesluk , K. Horwood , A. Szczesna , C. Martin , Y. Ragulin , M. Zukin , C. Helwig , M. Falk , C. Butts , F. A. Shepherd , Ann. Oncol. 2015, 26, 1134. 25722382 10.1093/annonc/mdv104

[70] C. Butts , M. A. Socinski , P. L. Mitchell , N. Thatcher , L. Havel , M. Krzakowski , S. Nawrocki , T. E. Ciuleanu , L. Bosquee , J. Manuel Trigo , A. Spira , L. Tremblay , J. Nyman , R. Ramlau , G. Wickart‐Johansson , P. Ellis , O. Gladkov , J. R. Pereira , W. E. E. Eberhardt , C. Helwig , A. Schroeder , F. A. Shepherd , S. T. Team , Lancet Oncol. 2014, 15, 59.24331154

[71] S. Cecco , E. Muraro , E. Giacomin , D. Martorelli , R. Lazzarini , P. Baldo , R. Dolcetti , Curr. Cancer Drug Targets 2011, 11, 85. 21062241 10.2174/156800911793743664

[72] L. Heesen , R. Jabulowsky , C. Loquai , J. Utikal , C. Gebhardt , J. Hassel , R. Kaufmann , A. Pinter , E. Derhovanessian , M. Diken , L. Kranz , H. Haas , S. Attig , A. Kuhn , P. Langguth , D. Schwarck‐Kokarakis , D. Jaeger , S. Grabbe , O. Tuereci , U. Sahin , Ann. Oncol. 2017, 28, 49P.

[73] N. L. Berinstein , M. Karkada , M. A. Morse , J. J. Nemunaitis , G. Chatta , H. Kaufman , K. Odunsi , R. Nigam , L. Sammatur , L. D. MacDonald , G. M. Weir , M. M. Stanford , M. Mansour , J. Transl. Med. 2012, 10, 156. 22862954 10.1186/1479-5876-10-156 PMC3479010

[74] N. L. Berinstein , M. Karkada , A. M. Oza , K. Odunsi , J. A. Villella , J. J. Nemunaitis , M. A. Morse , T. Pejovic , J. Bentley , M. Buyse , R. Nigam , G. M. Weir , L. D. MacDonald , T. Quinton , R. Rajagopalan , K. Sharp , A. Penwell , L. Sammatur , T. Burzykowski , M. M. Stanford , M. Mansour , OncoImmunology 2015, 4, e1026529. 26405584 10.1080/2162402X.2015.1026529 PMC4570133

[75] N. L. Berinstein , R. H. C. Van Der Jagt , M. C. Cheung , R. Buckstein , M. Karkada , T. Quinton , L. MacDonald , M. Stanford , R. Nigam , M. Mansour , J. Clin. Oncol. 2016, 34, e14578.

[76] M. Aoki , S. Ueda , H. Nishikawa , S. Kitano , M. Hirayama , H. Ikeda , H. Toyoda , K. Tanaka , M. Kanai , A. Takabayashi , H. Imai , T. Shiraishi , E. Sato , H. Wada , E. Nakayama , Y. Takei , N. Katayama , H. Shiku , S. Kageyama , Vaccine 2009, 27, 6854. 19761832 10.1016/j.vaccine.2009.09.018

[77] S. Kageyama , H. Wada , K. Muro , Y. Niwa , S. Ueda , H. Miyata , S. Takiguchi , S. H. Sugino , Y. Miyahara , H. Ikeda , N. Imai , E. Sato , T. Yamada , M. Osako , M. Ohnishi , N. Harada , T. Hishida , Y. Doki , H. Shiku , J. Transl. Med. 2013, 11, 246. 24093426 10.1186/1479-5876-11-246 PMC4015172

[78] H. Wada , E. Sato , A. Uenaka , M. Isobe , R. Kawabata , Y. Nakamura , S. Iwae , K. Yonezawa , M. Yamasaki , H. Miyata , Y. Doki , H. Shiku , A. A. Jungbluth , G. Ritter , R. Murphy , E. W. Hofftnan , L. J. Old , M. Monden , E. Nakayama , Int. J. Cancer 2008, 123, 2362. 18729190 10.1002/ijc.23810

[79] S. K. Libutti , G. F. Paciotti , A. A. Byrnes , H. R. Alexander , W. E. Gannon , M. Walker , G. D. Seidel , N. Yuldasheva , L. Tamarkin , Clin. Cancer Res. 2010, 16, 6139. 20876255 10.1158/1078-0432.CCR-10-0978 PMC3004980

[80] E. Hamilton , K. Blackwell , A. C. Hobeika , T. M. Clay , G. Broadwater , X. Ren , W. Chen , H. Castro , F. Lehmann , N. Spector , J. Wei , T. Osada , H. K. Lyerly , J. Transl. Med. 2012, 10, 28. 22325452 10.1186/1479-5876-10-28 PMC3306270

[81] M. Colombel , A. Heidenreich , L. Martinez‐Pineiro , M. Babjuk , I. Korneyev , C. Surcel , P. Yakovlev , R. Colombo , P. Radziszewski , F. Witjes , R. Schipper , P. Mulders , W. P. J. Witjes , Eur. Urol. 2014, 65, 509. 24268503 10.1016/j.eururo.2013.10.056

[82] S. A. Limentani , M. Campone , T. Dorval , G. Curigliano , R. de Boer , C. Vogel , S. White , T. Bachelot , J. L. Canon , M. Disis , Breast Cancer Res. Treat. 2016, 156, 319. 26993131 10.1007/s10549-016-3751-x

[83] J. Malhotra , D. Odea , J. E. Gomez , Lung Cancer Manage. 2015, 4, 31.

[84] R. Gutzmer , L. Rivoltini , E. Levchenko , A. Testori , J. Utikal , P. A. Ascierto , L. Demidov , J. J. Grob , R. Ridolfi , D. Schadendorf , P. Queirolo , A. Santoro , C. Loquai , B. Dreno , A. Hauschild , E. Schultz , T. P. Lesimple , N. Vanhoutte , B. Salaun , M. Gillet , S. Jarnjak , P. M. De Sousa Alves , J. Louahed , V. G. Brichard , F. F. Lehmann , ESMO Open 2016, 1, e000068. 27843625 10.1136/esmoopen-2016-000068 PMC5070281

[85] A. D. Cohen , N. Lendvai , S. Nataraj , N. Imai , A. A. Jungbluth , I. Tsakos , A. Rahman , A. H. C. Mei , H. Singh , K. Zarychta , S. Kim‐Schulze , A. Park , R. Venhaus , K. Alpaugh , S. Gnjatic , H. J. Cho , Cancer Immunol. Res. 2019, 7, 658. 30745365 10.1158/2326-6066.CIR-18-0198

[86] B. Dreno , J. F. Thompson , B. M. Smithers , M. Santinami , T. Jouary , R. Gutzmer , E. Levchenko , P. Rutkowski , J. J. Grob , S. Korovin , K. Drucis , F. Grange , L. Machet , P. Hersey , I. Krajsova , A. Testori , R. Conry , B. Guillot , W. H. J. Kruit , L. Demidov , J. A. Thompson , I. Bondarenko , J. Jaroszek , S. Puig , G. Cinat , A. Hauschild , J. J. Goeman , H. C. van Houwelingen , F. Ulloa‐Montoya , A. Callegaro , B. Dizier , B. Spiessens , M. Debois , V. G. Brichard , J. Louahed , P. Therasse , C. Debruyne , J. M. Kirkwood , Lancet Oncol. 2018, 19, 916. 29908991 10.1016/S1470-2045(18)30254-7

[87] C. L. Slingluff Jr. , G. R. Petroni , W. C. Olson , M. E. Smolkin , K. A. Chianese‐Bullock , I. S. Mauldin , K. T. Smith , D. H. Deacon , N. E. Varhegyi , S. B. Donnelly , C. M. Reed , K. Scott , N. V. Galeassi , W. W. Grosh , Cancer Immunol., Immunother. 2016, 65, 25. 26581199 10.1007/s00262-015-1770-9 PMC5010424

[88] J. L. McQuade , J. Homsi , C. A. Torres‐Cabala , R. Bassett , R. M. Popuri , M. L. James , L. M. Vence , W. J. Hwu , BMC Cancer 2018, 18, 1274. 30567529 10.1186/s12885-018-5193-9 PMC6300080

[89] T. Gargett , M. N. Abbas , P. Rolan , J. D. Price , K. M. Gosling , A. Ferrante , A. Ruszkiewicz , I. I. C. Atmosukarto , J. Altin , C. R. Parish , M. P. Brown , Cancer Immunol., Immunother. 2018, 67, 1461. 30014244 10.1007/s00262-018-2207-z PMC11028356

[90] J. S. Cebon , G. A. McArthur , W. Chen , I. D. Davis , M. E. Gore , J. F. Thompson , M. Millward , M. P. N. Findlay , R. Dunbar , C. H. H. Ottensmeier , R. R. Venhaus , P. D. Nathan , A. G. Dalgleish , V. Cerundolo , E. Maraskovsky , W. Hopkins , J. Marsden , B. M. Smithers , P. Hersey , T. R. J. Evans , J. Clin. Oncol. 2014, 32, 9050.

[91] O. Klein , I. D. Davis , G. A. McArthur , L. Chen , A. Haydon , P. Parente , N. Dimopoulos , H. Jackson , K. Xiao , E. Maraskovsky , W. Hopkins , R. Stan , W. Chen , J. Cebon , Cancer Immunol., Immunother. 2015, 64, 507. 25662405 10.1007/s00262-015-1656-x PMC11029160

[92] P. Tyagi , J. L. Santos , Drug Discovery Today 2018, 23, 1053. 29326081 10.1016/j.drudis.2018.01.017

[93] J. A. Kulkarni , P. R. Cullis , R. van der Meel , Nucleic Acid Ther. 2018, 28, 146. 29683383 10.1089/nat.2018.0721

[94] S. M. Goldinger , R. Dummer , P. Baumgaertner , D. Mihic‐Probst , K. Schwarz , A. Hammann‐Haenni , J. Willers , C. Geldhof , J. O. Prior , T. M. Kuendig , O. Michielin , M. F. Bachmann , D. E. Speiser , Eur. J. Immunol. 2012, 42, 3049. 22806397 10.1002/eji.201142361 PMC3549564

[95] E. Romano , H. Bichat , A. Stravodimou , P. Romero , S. E. Daniel , F. Triebel , O. Michielin , A. Harari , S. Leyvraz , J. Clin. Oncol. 2013, 31, e20011.

[96] D. Christensen , M. Henriksen‐Lacey , A. T. Kamath , T. Lindenstrom , K. S. Korsholm , J. P. Christensen , A. F. Rochat , P. H. Lambert , P. Andersen , C. A. Siegrist , Y. Perrie , E. M. Agger , J. Controlled Release 2012, 160, 468. 10.1016/j.jconrel.2012.03.016 22709414

[97] D. Christensen , K. S. Korsholm , P. Andersen , E. M. Agger , Expert Rev. Vaccines 2011, 10, 513. 21506648 10.1586/erv.11.17

[98] S. H. Park , S. K. Yang , S. K. Park , J. W. Kim , D. H. Yang , K. W. Jung , K. J. Kim , B. D. Ye , J. S. Byeon , S. J. Myung , J. H. Kim , Inflammatory Bowel Dis. 2014, 20, 69. 10.1097/01.MIB.0000437736.91712.a1 24284413

[99] K. Van Herck , A. Hens , I. De Coster , A. Vertruyen , J. Tolboom , M. Sarnecki , P. Van Damme , Pediatr. Infectious Dis. J. 2015, 34, e85. 25389920 10.1097/INF.0000000000000616

[100] H. H. Askling , L. Rombo , R. van Vollenhoven , I. Hallén , Å. Thörner , M. Nordin , C. Herzog , A. Kantele , Travel Med. Infectious Dis. 2014, 12, 134. 24529746 10.1016/j.tmaid.2014.01.005

[101] S. Buhler , V. K. Jaeger , S. Adler , B. Bannert , C. Brummerhoff , A. Ciurea , O. Distler , J. Franz , C. Gabay , N. Hagenbuch , C. Herzog , P. Hasler , K. Kling , D. Kyburz , R. Muller , M. J. Nissen , C. A. Siegrist , P. M. Villiger , U. A. Walker , C. Hatz , Rheumatology 2019, 10.1093/rheumatology/kez045. 30877773

[102] S. Esposito , P. Marchisio , V. Montinaro , S. Bianchini , G. J. Weverling , E. Pariani , A. Amendola , V. Fabiano , V. Pivetti , A. Zanetti , G. V. Zuccotti , Vaccine 2012, 30, 7005. 23059357 10.1016/j.vaccine.2012.09.069

[103] P. G. Cech , T. Aebi , M. S. Abdallah , M. Mpina , E. B. Machunda , N. Westerfeld , S. A. Stoffel , R. Zurbriggen , G. Pluschke , M. Tanner , C. Daubenberger , B. Genton , S. Abdulla , PLoS One 2011, 6, e22273. 21799810 10.1371/journal.pone.0022273 PMC3142124

[104] A. Loew‐Baselli , B. G. Pavlova , S. Fritsch , E. M. Poellabauer , W. Draxler , O. Kistner , U. Behre , R. Angermayr , J. Neugebauer , K. Kirsten , E. Foerster‐Waldl , R. Koellges , H. J. Ehrlich , P. N. Barrett , Vaccine 2012, 30, 5956. 22846396 10.1016/j.vaccine.2012.07.039

[105] J. T. van Dissel , S. A. Joosten , S. T. Hoff , D. Soonawala , C. Prins , D. A. Hokey , D. M. O'Dee , A. Graves , B. Thierry‐Carstensen , L. V. Andreasen , M. Ruhwald , A. W. de Visser , E. M. Agger , T. H. M. Ottenhoff , I. Kromann , P. Andersen , Vaccine 2014, 32, 7098. 25454872 10.1016/j.vaccine.2014.10.036

[106] F. Rose , K. Karlsen , P. R. Jensen , R. U. Jakobsen , G. K. Wood , K. D. Rand , H. Godiksen , P. Andersen , F. Follmann , C. Foged , J. Pharm. Sci. 2018, 107, 1690. 29452143 10.1016/j.xphs.2018.02.005

[107] V. R. G. Roman , K. J. Jensen , S. S. Jensen , C. Leo‐Hansen , S. Jespersen , D. d. S. Te , C. M. Rodrigues , C. M. Janitzek , L. Vinner , T. L. Katzenstein , P. Andersen , I. Kromann , L. V. Andreasen , I. Karlsson , A. Fomsgaard , AIDS Res. Hum. Retroviruses 2013, 29, 1504. 23634822 10.1089/AID.2013.0076

[108] I. Karlsson , L. Brandt , L. Vinner , I. Kromann , L. V. Andreasen , P. Andersen , J. Gerstoft , G. Kronborg , A. Fomsgaard , Clin. Immunol. 2013, 146, 120. 23314272 10.1016/j.clim.2012.12.005

[109] S. ZollaPazner , C. Alving , R. Belshe , P. Berman , S. Burda , P. Chigurupati , M. L. Clements , A. M. Duliege , J. L. Excler , C. Hioe , J. Kahn , M. J. McElrath , S. Sharpe , F. Sinangil , K. Steimer , M. C. Walker , N. Wassef , S. Xu , J. Infectious Dis. 1997, 175, 764. 9086128 10.1086/513969

[110] H. G. Kelly , S. J. Kent , A. K. Wheatley , Expert Rev. Vaccines 2019, 18, 269. 30707635 10.1080/14760584.2019.1578216

[111] T. Rampling , K. J. Ewer , G. Bowyer , C. M. Bliss , N. J. Edwards , D. Wright , R. O. Payne , N. Venkatraman , E. de Barra , C. M. Snudden , I. D. Poulton , H. de Graaf , P. Sukhtankar , R. Roberts , K. Ivinson , R. Weltzin , B. Y. Rajkumar , U. Wille‐Reece , C. K. Lee , C. F. Ockenhouse , R. E. Sinden , S. Gerry , A. M. Lawrie , J. Vekemans , D. Morelle , M. Lievens , R. W. Ballou , G. S. Cooke , S. N. Faust , S. Gilbert , A. V. S. Hill , J. Infectious Dis. 2016, 214, 772. 27307573 10.1093/infdis/jiw244 PMC4978377

[112] A. Olotu , F. Clement , E. Jongert , J. Vekemans , P. Njuguna , F. M. Ndungu , K. Marsh , G. Leroux‐Roels , P. Bejon , PLoS One 2014, 9, e115126. 25506706 10.1371/journal.pone.0115126 PMC4266636

[113] G. M. Warimwe , H. A. Fletcher , A. Olotu , S. T. Agnandji , A. V. S. Hill , K. Marsh , P. Bejon , BMC Med. 2013, 11, 184. 23962071 10.1186/1741-7015-11-184 PMC3765422

[114] A. Horowitz , J. C. R. Hafalla , E. King , J. Lusingu , D. Dekker , A. Leach , P. Moris , J. Cohen , J. Vekemans , T. Villafana , P. H. Corran , P. Bejon , C. J. Drakeley , L. von Seidlein , E. M. Riley , J. Immunol. 2012, 188, 5054. 22504653 10.4049/jimmunol.1102710 PMC3378032

[115] G. Bowyer , A. Grobbelaar , T. Rampling , N. Venkatraman , D. Morelle , R. W. Ballou , A. V. S. Hill , K. J. Ewer , Front. Immunol. 2018, 9, 1660. 30090099 10.3389/fimmu.2018.01660 PMC6068239

[116] B. Lell , S. Agnandji , I. von Glasenapp , S. Haertle , S. Oyakhiromen , S. Issifou , J. Vekemans , A. Leach , M. Lievens , M. C. Dubois , M. A. Demoitie , T. Carter , T. Villafana , W. R. Ballou , J. Cohen , P. G. Kremsner , PLoS One 2009, 4, e7611. 19859560 10.1371/journal.pone.0007611 PMC2763199

[117] S. T. Agnandji , R. Fendel , M. Mestre , M. Janssens , J. Vekemans , J. Held , F. Gnansounou , S. Haertle , I. von Glasenapp , S. Oyakhirome , L. Mewono , P. Moris , M. Lievens , M. A. Demoitie , P. M. Dubois , T. Villafana , E. Jongert , A. Olivier , J. Cohen , M. Esen , P. G. Kremsner , B. Lell , B. Mordmueller , PLoS One 2011, 6, e18559. 21494604 10.1371/journal.pone.0018559 PMC3073948

[118] D. Oyen , J. L. Torres , U. Wille‐Reece , C. F. Ockenhouse , D. Emerling , J. Glanville , W. Volkmuth , Y. Flores‐Garcia , F. Zavala , A. B. Ward , C. R. King , I. A. Wilson , Proc. Natl. Acad. Sci. USA 2018, 115, E5838. 29138320 10.1073/pnas.1715812114 PMC5715787

[119] K. E. Kester , J. F. Cummings , O. Ofori‐Anyinam , C. F. Ockenhouse , U. Krzych , P. Moris , R. Schwenk , R. A. Nielsen , Z. Debebe , E. Pinelis , L. Juompan , J. Williams , M. Dowler , V. A. Stewart , R. A. Wirtz , M. C. Dubois , M. Lievens , J. Cohen , W. R. Ballou , D. G. Heppner Jr., Rts , S. V. E. Grp , J. Infectious Dis. 2009, 200, 337. 19569965 10.1086/600120

[120] D. Witte , N. A. Cunliffe , A. M. Turner , E. Ngulube , O. Ofori‐Anyinam , J. Vekemans , P. Chimpeni , M. Lievens , T. P. Wilson , J. Njiram'madzi , Y. G. Mendoza , A. Leach , Pediatr. Infectious Dis. J. 2018, 37, 483. 29432383 10.1097/INF.0000000000001937

[121] D. N. Khalil , E. L. Smith , R. J. Brentjens , J. D. Wolchok , Nat. Rev. Clin. Oncol. 2016, 13, 273. 26977780 10.1038/nrclinonc.2016.25 PMC5551685

[122] B. Wiemann , C. O. Starnes , Pharmacol. Ther. 1994, 64, 529. 7724661 10.1016/0163-7258(94)90023-x

[123] A. M. Grimaldi , M. Incoronato , M. Salvatore , A. Soricelli , Nanomedicine 2017, 12, 2349. 28868980 10.2217/nnm-2017-0208

[124] E. M. E. Verdegaal , N. F. C. C. de Miranda , M. Visser , T. Harryvan , M. M. van Buuren , R. S. Andersen , S. R. Hadrup , C. E. van der Minne , R. Schotte , H. Spits , J. B. A. G. Haanen , E. H. W. Kapiteijn , T. N. Schumacher , S. H. van der Burg , Nature 2016, 536, 91. 27350335 10.1038/nature18945

[125] D. O'Sullivan , E. L. Pearce , Trends Immunol. 2015, 36, 71. 25601541 10.1016/j.it.2014.12.004 PMC4323623

[126] B. Feng , F. Zhou , B. Hou , D. Wang , T. Wang , Y. Fu , Y. Ma , H. Yu , Y. Li , Adv. Mater. 2018, 30, 1803001. 10.1002/adma.201803001 30063262

[127] A. D. Fesnak , C. H. June , B. L. Levine , Nat. Rev. Cancer 2016, 16, 566. 27550819 10.1038/nrc.2016.97 PMC5543811

[128] Q. Zhang , W. Wei , P. Wang , L. Zuo , F. Li , J. Xu , X. Xi , X. Gao , G. Ma , H. Xie , ACS Nano 2017, 11, 10724. 28921946 10.1021/acsnano.7b04955

[129] C. S. Chiang , Y. J. Lin , R. Lee , Y. H. Lai , H. W. Cheng , C. H. Hsieh , W. C. Shyu , S. Y. Chen , Nat. Nanotechnol. 2018, 13, 746. 29760523 10.1038/s41565-018-0146-7

[130] K. Cho , X. Wang , S. Nie , Z. Chen , D. M. Shin , Clin. Cancer Res. 2008, 14, 1310. 18316549 10.1158/1078-0432.CCR-07-1441

[131] F. B. Bombelli , C. A. Webster , M. Moncrieff , V. Sherwood , Lancet Oncol. 2014, 15, e22. 24384491 10.1016/S1470-2045(13)70333-4

[132] Q. Sun , Z. Zhou , N. Qiu , Y. Shen , Adv. Mater. 2017, 29, 1606628. 10.1002/adma.201606628 28234430

[133] F. Qiu , K. W. Becker , F. C. Knight , J. J. Baljon , S. Sevimli , D. Shae , P. Gilchuk , S. Joyce , J. T. Wilson , Biomaterials 2018, 182, 82. 30107272 10.1016/j.biomaterials.2018.07.052 PMC6778406

[134] S. Keller , J. T. Wilson , G. I. Patilea , H. B. Kern , A. J. Convertine , P. S. Stayton , J. Controlled Release 2014, 191, 24. 10.1016/j.jconrel.2014.03.041 PMC4156909 24698946

[135] R. Kuai , L. J. Ochyl , K. S. Bahjat , A. Schwendeman , J. J. Moon , Nat. Mater. 2017, 16, 489. 28024156 10.1038/nmat4822 PMC5374005

[136] R. A. Rosalia , L. J. Cruz , S. van Duikeren , A. T. Tromp , A. L. Silva , W. Jiskoot , T. de Gruijl , C. Lowik , J. Oostendorp , S. H. van der Burg , F. Ossendorp , Biomaterials 2015, 40, 88. 25465442 10.1016/j.biomaterials.2014.10.053

[137] H. Liu , K. D. Moynihan , Y. Zheng , G. L. Szeto , A. V. Li , B. Huang , D. S. Van Egeren , C. Park , D. J. Irvine , Nature 2014, 507, 519. 24531764 10.1038/nature12978 PMC4069155

[138] S. Y. Kim , Y. W. Noh , T. H. Kang , J. E. Kim , S. Kim , S. H. Um , D. B. Oh , Y. M. Park , Y. T. Lim , Biomaterials 2017, 130, 56. 28364631 10.1016/j.biomaterials.2017.03.034

[139] Y. Min , K. C. Roche , S. Tian , M. J. Eblan , K. P. McKinnon , J. M. Caster , S. Chai , L. E. Herring , L. Zhang , T. Zhang , J. M. DeSimone , J. E. Tepper , B. G. Vincent , J. S. Serody , A. Z. Wang , Nat. Nanotechnol. 2017, 12, 877. 28650437 10.1038/nnano.2017.113 PMC5587366

[140] H. Yuan , W. Jiang , C. A. von Roemeling , Y. Qie , X. Liu , Y. Chen , Y. Wang , R. E. Wharen , K. Yun , G. Bu , K. L. Knutson , B. Y. S. Kim , Nat. Nanotechnol. 2017, 12, 763. 28459470 10.1038/nnano.2017.69

[141] T. R. Fadel , F. A. Sharp , N. Vudattu , R. Ragheb , J. Garyu , D. Kim , E. Hong , N. Li , G. L. Haller , L. D. Pfefferle , S. Justesen , K. C. Herold , T. M. Fahmy , Nat. Nanotechnol. 2014, 9, 639. 25086604 10.1038/nnano.2014.154

[142] X. Zhang , C. Wang , J. Wang , Q. Hu , B. Langworthy , Y. Ye , W. Sun , J. Lin , T. Wang , J. Fine , H. Cheng , G. Dotti , P. Huang , Z. Gu , Adv. Mater. 2018, 30, 1707112. 10.1002/adma.201707112 29656492

[143] S. Li , Y. Liu , C. Xu , S. Shen , R. Sun , X. Du , J. Xia , Y. Zhu , J. Wang , J. Controlled Release 2016, 231, 17. 10.1016/j.jconrel.2016.01.044 26829099

[144] C. Wang , W. Sun , Y. Ye , Q. Hu , H. N. Bomba , Z. Gu , Nat. Biomed. Eng. 2017, 1, UNSP 0011.

[145] Y. Mi , C. C. Smith , F. Yang , Y. Qi , K. C. Roche , J. S. Serody , B. G. Vincent , A. Z. Wang , Adv. Mater. 2018, 30, 1706098. 10.1002/adma.201706098 PMC6003883 29691900

[146] C. B. Rodell , S. P. Arlauckas , M. F. Cuccarese , C. S. Garris , R. L. M. S. Ahmed , R. H. Kohler , M. J. Pittet , R. Weissleder , Nat. Biomed. Eng. 2018, 2, 578. 10.1038/s41551-018-0236-8 PMC6192054 31015631

[147] Y. W. Choo , M. Kang , H. Y. Kim , J. Han , S. Kang , J. R. Lee , G. J. Jeong , S. P. Kwon , S. Y. Song , S. Go , M. Jung , J. Hong , B. S. Kim , ACS Nano 2018, 12, 8977. 30133260 10.1021/acsnano.8b02446

[148] A. K. Fuchs , T. Syrovets , K. A. Haas , C. Loos , A. Musyanovych , V. Mailaender , K. Landfester , T. Simmet , Biomaterials 2016, 85, 78. 26854393 10.1016/j.biomaterials.2016.01.064

[149] V. Kodali , M. H. Littke , S. C. Tilton , J. G. Teeguarden , L. Shi , C. W. Frevert , W. Wang , J. G. Pounds , B. D. Thrall , ACS Nano 2013, 7, 6997. 23808590 10.1021/nn402145t PMC3756554

[150] S. Zanganeh , G. Hutter , R. Spitler , O. Lenkov , M. Mahmoudi , A. Shaw , J. S. Pajarinen , H. Nejadnik , S. Goodman , M. Moseley , L. M. Coussens , H. E. Daldrup‐Link , Nat. Nanotechnol. 2016, 11, 986. 27668795 10.1038/nnano.2016.168 PMC5198777

[151] M. P. Plebanek , D. Bhaumik , P. J. Bryce , C. S. Thaxton , Mol. Cancer Ther. 2018, 17, 686. 29282300 10.1158/1535-7163.MCT-17-0981 PMC5935575

[152] M. S. Sasso , G. Lollo , M. Pitorre , S. Solito , L. Pinton , S. Valpione , G. Bastiat , S. Mandruzzato , V. Bronte , I. Marigo , J. P. Benoit , Biomaterials 2016, 96, 47. 27135716 10.1016/j.biomaterials.2016.04.010

[153] Y. Wang , W. Song , M. Hu , S. An , L. Xu , J. Li , K. A. Kinghorn , R. Liu , L. Huang , Adv. Funct. Mater. 2018, 28, 1802847.

[154] Z. Xu , Y. Wang , L. Zhang , L. Huang , ACS Nano 2014, 8, 3636. 24580381 10.1021/nn500216y PMC4004320

[155] D. Schmid , C. G. Park , C. A. Hartl , N. Subedi , A. N. Cartwright , R. B. Puerto , Y. Zheng , J. Maiarana , G. J. Freeman , K. W. Wucherpfennig , D. J. Irvine , M. S. Goldberg , Nat. Commun. 2017, 8, 1747. 29170511 10.1038/s41467-017-01830-8 PMC5700944

[156] M. Kong , J. Tang , Q. Qiao , T. Wu , Y. Qi , S. Tan , X. Gao , Z. Zhang , Theranostics 2017, 7, 3276. 28900509 10.7150/thno.19987 PMC5595131

[157] W. Tang , J. Yang , Y. Yuan , Z. Zhao , Z. Lian , G. Liang , Nanoscale 2017, 9, 6529. 28466929 10.1039/c6nr09895a

[158] J. Xue , Z. Zhao , L. Zhang , L. Xue , S. Shen , Y. Wen , Z. Wei , L. Wang , L. Kong , H. Sun , Q. Ping , R. Mo , C. Zhang , Nat. Nanotechnol. 2017, 12, 692. 28650441 10.1038/nnano.2017.54

[159] S. Li , Y. Zhang , J. Wang , Y. Zhao , T. Ji , X. Zhao , Y. Ding , X. Zhao , R. Zhao , F. Li , X. Yang , S. Liu , Z. Liu , J. Lai , A. K. Whittaker , G. J. Anderson , J. Wei , G. Nie , Nat. Biomed. Eng. 2017, 1, 667. 31015598 10.1038/s41551-017-0115-8

[160] W. Song , J. Kuang , C. Li , M. Zhang , D. Zheng , X. Zeng , C. Liu , X. Zhang , ACS Nano 2018, 12, 1978. 29420012 10.1021/acsnano.7b09112

[161] G. Yang , L. Xu , J. Xu , R. Zhang , G. Song , Y. Chao , L. Feng , F. Han , Z. Dong , B. Li , Z. Liu , Nano Lett. 2018, 18, 2475. 29565139 10.1021/acs.nanolett.8b00040

[162] C. Shi , T. Liu , Z. Guo , R. Zhuang , X. Zhang , X. Chen , Nano Lett. 2018, 18, 7330. 30339753 10.1021/acs.nanolett.8b03568

[163] L. Meng , Y. Cheng , X. Tong , S. Gan , Y. Ding , Y. Zhang , C. Wang , L. Xu , Y. Zhu , J. Wu , Y. Hu , A. Yuan , ACS Nano 2018, 12, 8308. 30102510 10.1021/acsnano.8b03590

[164] Q. Chen , J. Chen , Z. Yang , J. Xu , L. Xu , C. Liang , X. Han , Z. Liu , Adv. Mater. 2019, 31, 1802228. 10.1002/adma.201802228 30663118

[165] Y. Tao , E. Ju , Z. Liu , K. Dong , J. Ren , X. Qu , Biomaterials 2014, 35, 6646. 24818880 10.1016/j.biomaterials.2014.04.073

[166] J. Banchereau , R. M. Steinman , Nature 1998, 392, 245. 9521319 10.1038/32588

[167] T. Kawai , S. Akira , Nat. Immunol. 2010, 11, 373. 20404851 10.1038/ni.1863

[168] A. C. Gomes , M. Mohsen , M. F. Bachmann , Vaccines 2017, 5, 6. 10.3390/vaccines5010006 PMC5371742 28216554

[169] D. Hawiger , K. Inaba , Y. Dorsett , M. Guo , K. Mahnke , M. Rivera , J. V. Ravetch , R. M. Steinman , M. C. Nussenzweig , J. Exp. Med. 2001, 194, 769. 11560993 10.1084/jem.194.6.769 PMC2195961

[170] C. Foged , B. Brodin , S. Frokjaer , A. Sundblad , Int. J. Pharm. 2005, 298, 315. 15961266 10.1016/j.ijpharm.2005.03.035

[171] Y. J. Kwon , E. James , N. Shastri , J. M. J. Frechet , Proc. Natl. Acad. Sci. USA 2005, 102, 18264. 16344458 10.1073/pnas.0509541102 PMC1317987

[172] A. Smith , M. Perelman , M. Hinchcliffe , Hum. Vaccines Immunother. 2014, 10, 797. 10.4161/hv.27449 PMC4130252 24346613

[173] S. T. Reddy , A. Rehor , H. G. Schmoekel , J. A. Hubbell , M. A. Swartz , J. Controlled Release 2006, 112, 26. 10.1016/j.jconrel.2006.01.006 16529839

[174] S. T. Reddy , A. J. Van Der Vlies , E. Simeoni , V. Angeli , G. J. Randolph , C. P. O'Neil , L. K. Lee , M. A. Swartz , J. A. Hubbell , Nat. Biotechnol. 2007, 25, 1159. 17873867 10.1038/nbt1332

[175] N. L. Bennewitz , J. E. Babensee , Biomaterials 2005, 26, 2991. 15603794 10.1016/j.biomaterials.2004.08.023

[176] H. Gong , J. Xiang , L. Xu , X. Song , Z. Dong , R. Peng , Z. Liu , Nanoscale 2015, 7, 19282. 26530014 10.1039/c5nr06081h

[177] K. A. Collins , R. Snaith , M. G. Cottingham , S. C. Gilbert , A. V. S. Hill , Sci. Rep. 2017, 7, e34445. 10.1038/srep46621 PMC5395940 28422178

[178] M. Obeid , A. Tesniere , F. Ghiringhelli , G. M. Fimia , L. Apetoh , J. L. Perfettini , M. Castedo , G. Mignot , T. Panaretakis , N. Casares , D. Metivier , N. Larochette , P. van Endert , F. Ciccosanti , M. Piacentini , L. Zitvogel , G. Kroemer , Nat. Med. 2007, 13, 54. 17187072 10.1038/nm1523

[179] M. P. Chao , S. Jaiswal , R. Weissman‐Tsukamoto , A. A. Alizadeh , A. J. Gentles , J. Volkmer , K. Weiskopf , S. B. Willingham , T. Raveh , C. Y. Park , R. Majeti , I. L. Weissman , Sci. Transl. Med. 2010, 2, 63ra94. 10.1126/scitranslmed.3001375 PMC4126904 21178137

[180] G. Zhu , F. Zhang , Q. Ni , G. Niu , X. Chen , ACS Nano 2017, 11, 2387. 28277646 10.1021/acsnano.7b00978

[181] P. Guermonprez , L. Saveanu , M. Kleijmeer , J. Davoust , P. van Endert , S. Amigorena , Nature 2003, 425, 397. 14508489 10.1038/nature01911

[182] S. Burgdorf , C. Schoelz , A. Kautz , R. Tampe , C. Kurts , Nat. Immunol. 2008, 9, 558. 18376402 10.1038/ni.1601

[183] L. M. Kaminskas , C. J. H. Porter , Adv. Drug Delivery Rev. 2011, 63, 890. 10.1016/j.addr.2011.05.016 21683746

[184] A. Stano , E. A. Scott , K. Y. Dane , M. A. Swartz , J. A. Hubbell , Biomaterials 2013, 34, 4339. 23478034 10.1016/j.biomaterials.2013.02.024

[185] J. Zhang , L. Wu , F. Meng , Z. Wang , C. Deng , H. Liu , Z. Zhong , Langmuir 2012, 28, 2056. 22188099 10.1021/la203843m

[186] A. J. Convertine , D. S. W. Benoit , C. L. Duvall , A. S. Hoffman , P. S. Stayton , J. Controlled Release 2009, 133, 221. 10.1016/j.jconrel.2008.10.004 PMC3110267 18973780

[187] M. Oelke , M. V. Maus , D. Didiano , C. H. June , A. Mackensen , J. P. Schneck , Nat. Med. 2003, 9, 619. 12704385 10.1038/nm869

[188] B. Prakken , M. Wauben , D. Genini , R. Samodal , J. Barnett , A. Mendivil , L. Leoni , S. Albani , Nat. Med. 2000, 6, 1406. 11100129 10.1038/82231

[189] K. Perica , A. Tu , A. Richter , J. G. Bieler , M. Edidin , J. P. Schneck , ACS Nano 2014, 8, 2252. 24564881 10.1021/nn405520d PMC4004316

[190] A. K. Kosmides , R. A. Meyer , J. W. Hickey , K. Aje , K. N. Cheung , J. J. Green , J. P. Schneck , Biomaterials 2017, 118, 16. 27940380 10.1016/j.biomaterials.2016.11.038 PMC5207804

[191] M. V. Maus , A. K. Thomas , D. G. B. Leonard , D. Allman , K. Addya , K. Schlienger , J. L. Riley , C. H. June , Nat. Biotechnol. 2002, 20, 143. 11821859 10.1038/nbt0202-143

[192] Y. Ishida , Y. Agata , K. Shibahara , T. Honjo , EMBO J. 1992, 11, 3887. 1396582 10.1002/j.1460-2075.1992.tb05481.x PMC556898

[193] J. A. James , T. Gross , R. H. Scofield , J. B. Harley , J. Exp. Med. 1995, 181, 453. 7530756 10.1084/jem.181.2.453 PMC2191871

[194] M. F. Krummel , J. P. Allison , J. Exp. Med. 1996, 183, 2533. 8676074 10.1084/jem.183.6.2533 PMC2192613

[195] J. E. Rosenberg , J. Hoffman‐Censits , T. Powles , M. S. van der Heijden , A. V. Balar , A. Necchi , N. Dawson , P. H. O'Donnell , A. Balmanoukian , Y. Loriot , S. Srinivas , M. M. Retz , P. Grivas , R. W. Joseph , M. D. Galsky , M. T. Fleming , D. P. Petrylak , J. L. Perez‐Gracia , H. A. Burris , D. Castellano , C. Canil , J. Bellmunt , D. Bajorin , D. Nickles , R. Bourgon , G. M. Frampton , N. Cui , S. Mariathasan , O. Abidoye , G. D. Fine , R. Dreicer , Lancet 2016, 387, 1909. 26952546 10.1016/S0140-6736(16)00561-4 PMC5480242

[196] T. L. Whiteside , Semin. Cancer Biol. 2006, 16, 3.16153857

[197] L. Cheng , Y. Wang , L. Huang , Mol. Ther. 2017, 25, 1665. 28284981 10.1016/j.ymthe.2017.02.007 PMC5498801

[198] D. A. A. Vignali , L. W. Collison , C. J. Workman , Nat. Rev. Immunol. 2008, 8, 523. 18566595 10.1038/nri2343 PMC2665249

[199] H. Jonuleit , T. Bopp , C. Becker , Nanomedicine 2016, 11, 2699. 27654070 10.2217/nnm-2016-0197

[200] N. Mueller‐Hermelink , H. Braumueller , B. Pichler , T. Wieder , R. Mailhammer , K. Schaak , K. Ghoreschi , A. Yazdi , R. Haubner , C. A. Sander , R. Mocikat , M. Schwaiger , I. Foerster , R. Huss , W. A. Weber , M. Kneilling , M. Roecken , Cancer Cell 2008, 13, 507. 18538734 10.1016/j.ccr.2008.04.001

[201] K. Sato , N. Sato , B. Xu , Y. Nakamura , T. Nagaya , P. L. Choyke , Y. Hasegawa , H. Kobayashi , Sci. Transl. Med. 2016, 8, 352ra110. 10.1126/scitranslmed.aaf6843 PMC7780242 27535621

[202] W. Song , S. N. Musetti , L. Huang , Biomaterials 2017, 148, 16. 28961532 10.1016/j.biomaterials.2017.09.017 PMC5762124

[203] A. Sica , A. Saccani , B. Bottazzi , N. Polentarutti , A. Vecchi , J. Van Damme , A. Mantovani , J. Immunol. 2000, 164, 762. 10623821 10.4049/jimmunol.164.2.762

[204] B. Z. Qian , J. W. Pollard , Cell 2010, 141, 39. 20371344 10.1016/j.cell.2010.03.014 PMC4994190

[205] S. K. Biswas , A. Mantovani , Nat. Immunol. 2010, 11, 889. 20856220 10.1038/ni.1937

[206] T. Chanmee , P. Ontong , K. Konno , N. Itano , Cancers 2014, 6, 1670. 25125485 10.3390/cancers6031670 PMC4190561

[207] H. Wang , D. J. Mooney , Nat. Mater. 2018, 17, 761. 30104668 10.1038/s41563-018-0147-9

[208] A. A. Shvedova , A. V. Tkach , E. R. Kisin , T. Khaliullin , S. Stanley , D. W. Gutkin , A. Star , Y. Chen , G. V. Shurin , V. E. Kagan , M. R. Shurin , Small 2013, 9, 1691. 22996965 10.1002/smll.201201470 PMC3624038

[209] W. Park , Y. J. Heo , D. K. Han , Biomater. Res. 2018, 22, 24. 30275967 10.1186/s40824-018-0133-y PMC6158870

[210] F. Su , K. R. Kozak , S. Imaizumi , F. Gao , M. W. Amneus , V. Grijalva , C. Ng , A. Wagner , G. Hough , G. Farias‐Eisner , Proc. Natl. Acad. Sci. USA 2010, 107, 19997. 21041624 10.1073/pnas.1009010107 PMC2993420

[211] M. Zamanian‐Daryoush , D. Lindner , T. C. Tallant , Z. Wang , J. Buffa , E. Klipfell , Y. Parker , D. Hatala , P. Parsons‐Wingerter , P. Rayman , M. S. S. Yusufishaq , E. A. Fisher , J. D. Smith , J. Finke , J. A. DiDonato , S. L. Hazen , J. Biol. Chem. 2013, 288, 21237. 23720750 10.1074/jbc.M113.468967 PMC3774392

[212] E. Suzuki , V. Kapoor , A. S. Jassar , L. R. Kaiser , S. M. Albelda , Clin. Cancer Res. 2005, 11, 6713. 16166452 10.1158/1078-0432.CCR-05-0883

[213] J. Hillion , S. S. Smail , F. Di Cello , A. Belton , S. N. Shah , T. Huso , A. Schuldenfrei , D. M. Nelson , L. Cope , N. Campbell , C. Karikari , A. Aderinto , A. Maitra , D. L. Huso , L. M. S. Resar , Pancreatology 2012, 12, 372. 22898640 10.1016/j.pan.2012.05.005 PMC3466102

[214] S. Zelenay , A. G. van der Veen , J. P. Boettcher , K. J. Snelgrove , N. Rogers , S. E. Acton , P. Chakravarty , M. R. Girotti , R. Marais , S. A. Quezada , E. Sahai , C. Reis e Sousa , Cell 2015, 162, 1257. 26343581 10.1016/j.cell.2015.08.015 PMC4597191

[215] L. Xian , D. Georgess , T. Huso , L. Cope , A. Belton , Y. T. Chang , W. Kuang , Q. Gu , X. Zhang , S. Senger , A. Fasano , D. L. Huso , A. J. Ewald , L. M. S. Resar , Nat. Commun. 2017, 8, 15008. 28452345 10.1038/ncomms15008 PMC5414379

[216] J. M. Yingling , K. L. Blanchard , J. S. Sawyer , Nat. Rev. Drug Discovery 2004, 3, 1011. 15573100 10.1038/nrd1580

[217] R. B. Holmgaard , D. Zamarin , Y. Li , B. Gasmi , D. H. Munn , J. P. Allison , T. Merghoub , J. D. Wolchok , Cell Rep. 2015, 13, 412. 26411680 10.1016/j.celrep.2015.08.077 PMC5013825

[218] D. H. Munn , A. L. Mellor , J. Clin. Invest. 2007, 117, 1147. 17476344 10.1172/JCI31178 PMC1857253

[219] S. K. Rajendrakumar , S. Uthaman , C. S. Cho , I. K. Park , Biomacromolecules 2018, 19, 1869. 29677439 10.1021/acs.biomac.8b00460

[220] L. A. Emens , G. Middleton , Cancer Immunol. Res. 2015, 3, 436. 25941355 10.1158/2326-6066.CIR-15-0064 PMC5012642

[221] D. Zheng , J. Chen , J. Zhu , L. Rong , B. Li , Q. Lei , J. Fan , M. Zou , C. Li , S. Cheng , Z. Xu , X. Zhang , Nano Lett. 2016, 16, 4341. 27327876 10.1021/acs.nanolett.6b01432

[222] L. Galluzzi , A. Buque , O. Kepp , L. Zitvogel , G. Kroemer , Nat. Rev. Immunol. 2017, 17, 97. 27748397 10.1038/nri.2016.107

[223] K. Kono , K. Mimura , R. Kiessling , Cell Death Dis. 2013, 4, e688. 23788045 10.1038/cddis.2013.207 PMC3702303

[224] H. Y. Yoon , S. T. SeIvan , Y. Yang , M. J. Kim , D. K. Yi , I. C. Kwon , K. Kim , Biomaterials 2018, 178, 597. 29576282 10.1016/j.biomaterials.2018.03.036

[225] D. V. Krysko , A. D. Garg , A. Kaczmarek , O. Krysko , P. Agostinis , P. Vandenabeele , Nat. Rev. Cancer 2012, 12, 860. 23151605 10.1038/nrc3380

[226] R. R. Love , H. Leventhal , D. V. Easterling , D. R. Nerenz , Cancer 1989, 63, 604. 2912536 10.1002/1097-0142(19890201)63:3<604::aid-cncr2820630334>3.0.co;2-2

[227] P. Y. Teo , C. Yang , L. M. Whilding , A. C. Parente‐Pereira , J. Maher , A. J. T. George , J. L. Hedrick , Y. Y. Yang , S. Ghaem‐Maghami , Adv. Healthcare Mater. 2015, 4, 1180. 10.1002/adhm.201500089 25866054

[228] W. Song , L. Shen , Y. Wang , Q. Liu , T. J. Goodwin , J. Li , O. Dorosheva , T. Liu , R. Liu , L. Huang , Nat. Commun. 2018, 9, 2237. 29884866 10.1038/s41467-018-04605-x PMC5993831

[229] R. Masetti , F. Vendemini , D. Zama , C. Biagi , P. Gasperini , A. Pession , Expert Rev. Anticancer Ther. 2012, 12, 1191. 23098119 10.1586/era.12.101

[230] A. Gros , V. Syvannarath , L. Lamrani , V. Ollivier , S. Loyau , T. Goerge , B. Nieswandt , M. Jandrot‐Perrus , B. Ho‐Tin‐Noe , Blood 2015, 126, 1017. 26036804 10.1182/blood-2014-12-617159

[231] Y. Boulaftali , P. R. Hess , T. M. Getz , A. Cholka , M. Stolla , N. Mackman , A. P. Owens III , J. Ware , M. L. Kahn , W. Bergmeier , J. Clin. Invest. 2013, 123, 908. 23348738 10.1172/JCI65154 PMC3561801

[232] K. Ni , G. Lan , S. S. Veroneau , X. Duan , Y. Song , W. Lin , Nat. Commun. 2018, 9, 4321. 30333489 10.1038/s41467-018-06655-7 PMC6193046

[233] J. T. Robinson , S. M. Tabakman , Y. Liang , H. Wang , H. Sanchez Casalongue , D. Vinh , H. Dai , J. Am. Chem. Soc. 2011, 133, 6825. 21476500 10.1021/ja2010175

[234] R. Vankayala , K. C. Hwang , Adv. Mater. 2018, 30, 1706320. 10.1002/adma.201706320 29577458

[235] M. Korbelik , W. Zhang , S. Merchant , Cancer Immunol., Immunother. 2011, 60, 1431. 21644033 10.1007/s00262-011-1047-x PMC11028986

[236] P. Mroz , J. T. Hashmi , Y. Y. Huang , N. Lang , M. R. Hamblin , Expert Rev. Clin. Immunol. 2011, 7, 75. 21162652 10.1586/eci.10.81 PMC3060712

[237] E. E. Sweeney , J. Cano‐Mejia , R. Fernandes , Small 2018, 14, 1800678. 10.1002/smll.201800678 29665282

[238] C. N. Coleman , Radiother. Oncol. 1993, 28, 1. 8234865 10.1016/0167-8140(93)90179-c

[239] R. E. Roses , J. Datta , B. J. Czerniecki , Radiat. Res. 2014, 182, 211. 24992163 10.1667/RR13495.1 PMC4143901

[240] M. A. Postow , M. K. Callahan , C. A. Barker , Y. Yamada , J. Yuan , S. Kitano , Z. Mu , T. Rasalan , M. Adamow , E. Ritter , N. Engl. J. Med. 2012, 366, 925. 22397654 10.1056/NEJMoa1112824 PMC3345206

[241] D. Schaue , M. W. Xie , J. A. Ratikan , W. H. McBride , Front. Oncol. 2012, 2, 90. 22912933 10.3389/fonc.2012.00090 PMC3421147

[242] H. E. Barker , J. T. Paget , A. A. Khan , K. J. Harrington , Nat. Rev. Cancer 2015, 15, 409. 26105538 10.1038/nrc3958 PMC4896389

[243] J. M. Brown , W. R. Wilson , Nat. Rev. Cancer 2004, 4, 437. 15170446 10.1038/nrc1367

[244] M. S. Draz , Y. J. Wang , F. F. Chen , Y. Xu , H. Shafiee , Adv. Funct. Mater. 2017, 27, 1604139. 29180949 10.1002/adfm.201604139 PMC5701658

[245] M. Vetro , D. Safari , S. Fallarini , K. Salsabila , M. Lahmann , S. Penadés , L. Lay , M. Marradi , F. Compostella , Nanomedicine 2017, 12, 13. 27879152 10.2217/nnm-2016-0306

[246] Y. Liu , H. Wang , D. Li , Y. Tian , W. Liu , L. Zhang , W. Zheng , Y. Hao , J. Liu , Z. Yang , Y. Shao , X. Jiang , Nanoscale Horiz. 2016, 1, 135. 10.1039/c5nh00064e 32260635

[247] L. Deng , T. Mohan , T. Z. Chang , G. X. Gonzalez , Y. Wang , Y. M. Kwon , S. M. Kang , R. W. Compans , J. A. Champion , B. Wang , Nat. Commun. 2018, 9, 359. 29367723 10.1038/s41467-017-02725-4 PMC5783933

[248] J. Yang , S. M. Shim , T. Q. Nguyen , E. H. Kim , K. Kim , Y. T. Lim , M. H. Sung , R. Webby , H. Poo , Sci. Rep. 2017, 7, 44839. 28322289 10.1038/srep44839 PMC5359587

[249] C. A. Schütz , L. Juillerat‐Jeanneret , H. Mueller , I. Lynch , M. Riediker , Nanomedicine 2013, 8, 449. 23477336 10.2217/nnm.13.8

[250] D. J. Irvine , M. C. Hanson , K. Rakhra , T. Tokatlian , Chem. Rev. 2015, 115, 11109. 26154342 10.1021/acs.chemrev.5b00109 PMC4688911

[251] Y. Liu , Y. L. Balachandran , D. Li , Y. Shao , X. Jiang , ACS Nano 2016, 10, 3589. 26844372 10.1021/acsnano.5b08025

[252] Y. Tian , H. Wang , Y. Liu , L. Mao , W. Chen , Z. Zhu , W. Liu , W. Zheng , Y. Zhao , D. Kong , Z. Yang , W. Zhang , Y. Shao , X. Jiang , Nano Lett. 2014, 14, 1439. 24564254 10.1021/nl404560v

[253] X. Wei , G. Zhang , D. Ran , N. Krishnan , R. H. Fang , W. Gao , S. A. Spector , L. Zhang , Adv. Mater. 2018, 30, 1802233. 10.1002/adma.201802233 PMC6334303 30252965

[254] R. B. Jones , S. Mueller , S. Kumari , V. Vrbanac , S. Genel , A. M. Tager , T. M. Allen , B. D. Walker , D. J. Irvine , Biomaterials 2017, 117, 44. 27936416 10.1016/j.biomaterials.2016.11.048 PMC5204257

[255] T. Tokatlian , B. J. Read , C. A. Jones , D. W. Kulp , S. Menis , J. Y. H. Chang , J. M. Steichen , S. Kumari , J. D. Allen , E. L. Dane , A. Liguori , M. Sangesland , D. Lingwood , M. Crispin , W. R. Schief , D. J. Irvine , Science 2019, 363, 649. 30573546 10.1126/science.aat9120 PMC6420719

[256] L. Wang , T. Z. Chang , Y. He , J. R. Kim , S. Wang , T. Mohan , Z. Berman , S. M. Tompkins , R. A. Tripp , R. W. Compans , J. A. Champion , B. Wang , Nanomed.: Nanotechnol., Biol. Med. 2017, 13, 253.

[257] L. Deng , T. Z. Chang , Y. Wang , S. Li , S. Wang , S. Matsuyama , G. Yu , R. W. Compans , J. Li , M. R. Prausnitz , J. A. Champion , B. Wang , Proc. Natl. Acad. Sci. USA 2018, 115, E7758. 30065113 10.1073/pnas.1805713115 PMC6099848

[258] F. Dakterzada , A. M. Mobarez , M. H. Roudkenar , A. Mohsenifar , Vaccine 2016, 34, 1472. 26868080 10.1016/j.vaccine.2016.01.041

[259] C. M. J. Hu , R. H. Fang , B. T. Luk , L. Zhang , Nat. Nanotechnol. 2013, 8, 933. 24292514 10.1038/nnano.2013.254 PMC3878426

[260] F. Wang , R. H. Fang , B. T. Luk , C. M. J. Hu , S. Thamphiwatana , D. Dehaini , P. Angsantikul , A. V. Kroll , Z. Pang , W. Gao , W. Lu , L. Zhang , Adv. Funct. Mater. 2016, 26, 1628. 27325913 10.1002/adfm.201505231 PMC4912041

[261] X. Wei , J. Gao , F. Wang , M. Ying , P. Angsantikul , A. V. Kroll , J. Zhou , W. Gao , W. Lu , R. H. Fang , L. Zhang , Adv. Mater. 2017, 29, 1701644. 10.1002/adma.201701644 PMC5581250 28656663

[262] W. Gao , R. H. Fang , S. Thamphiwatana , B. T. Luk , J. Li , P. Angsantikul , Q. Zhang , C. M. J. Hu , L. Zhang , Nano Lett. 2015, 15, 1403. 25615236 10.1021/nl504798g PMC4399974

[263] Y. Liu , C. Chen , Adv. Drug Delivery Rev. 2016, 103, 76. 10.1016/j.addr.2016.02.010 26952542

[264] P. R. Harrigan , M. Whaley , J. S. G. Montaner , AIDS 1999, 13, F59. 10371167 10.1097/00002030-199905280-00001

[265] P. R. Harrigan , R. S. Hogg , W. W. Y. Dong , B. Yip , B. Wynhoven , J. Woodward , C. J. Brumme , Z. L. Brumme , T. Mo , C. S. Alexander , J. S. G. Montaner , J. Infectious Dis. 2005, 191, 339. 15633092 10.1086/427192

[266] G. K. Lewis , M. Pazgier , A. L. DeVico , Immunol. Rev. 2017, 275, 271. 28133809 10.1111/imr.12510 PMC5642910

[267] R. H. Fang , A. V. Kroll , W. Gao , L. Zhang , Adv. Mater. 2018, 30, 1706759. 10.1002/adma.201706759 PMC5984176 29582476

[268] J. Ingale , A. Stano , J. Guenaga , S. K. Sharma , D. Nemazee , M. B. Zwick , R. T. Wyatt , Cell Rep. 2016, 15, 1986. 27210756 10.1016/j.celrep.2016.04.078 PMC4889521

[269] F. Krammer , P. Palese , Nat. Rev. Drug Discovery 2015, 14, 167. 25722244 10.1038/nrd4529

[270] M. L. Bookstaver , S. J. Tsai , J. S. Bromberg , C. M. Jewell , Trends Immunol. 2018, 39, 135. 29249461 10.1016/j.it.2017.10.002 PMC5914493

[271] S. Shiosaka , H. Kiyama , A. Wanaka , T. Masaya , Brain Res. 1986, 382, 399. 2875768 10.1016/0006-8993(86)91352-1

[272] A. F. Radovic‐Moreno , N. Chernyak , C. C. Mader , S. Nallagatla , R. S. Kang , L. Hao , D. A. Walker , T. L. Halo , T. J. Merkel , C. H. Rische , S. Anantatmula , M. Burkhart , C. A. Mirkin , S. M. Gryaznov , Proc. Natl. Acad. Sci. USA 2015, 112, 3892. 25775582 10.1073/pnas.1502850112 PMC4386353

[273] D. M. Morens , G. K. Folkers , A. S. Fauci , Nature 2004, 430, 242. 15241422 10.1038/nature02759 PMC7094993

[274] R. P. Mishra , E. Oviedo‐Orta , P. Prachi , R. Rappuoli , F. Bagnoli , Curr. Opin. Microbiol. 2012, 15, 596. 22981392 10.1016/j.mib.2012.08.002

[275] L. C. W. Lin , S. Chattopadhyay , J. C. Lin , C. M. J. Hu , Adv. Healthcare Mater. 2018, 7, 1701395.

[276] M. Zaman , M. F. Good , I. Toth , Methods 2013, 60, 226. 23623821 10.1016/j.ymeth.2013.04.014

[277] A. Bafica , C. A. Scanga , C. G. Feng , C. Leifer , A. Cheever , A. Sher , J. Exp. Med. 2005, 202, 1715. 16365150 10.1084/jem.20051782 PMC2212963

[278] P. M. Moyle , W. Dai , Y. Zhang , M. R. Batzloff , M. F. Good , I. Toth , Bioconjugate Chem. 2014, 25, 965. 10.1021/bc500108b 24712905

[279] V. Pavot , N. Climent , N. Rochereau , F. Garcia , C. Genin , G. Tiraby , F. Vernejoul , E. Perouzel , T. Lioux , B. Verrier , S. Paul , Biomaterials 2016, 75, 327. 26539801 10.1016/j.biomaterials.2015.10.034

[280] M. Shoham , Future Med. Chem. 2011, 3, 775. 21644821 10.4155/fmc.11.43

[281] P. O'Hanley , G. Lalonde , G. Ji , Infect. Immun. 1991, 59, 1153. 1671776 10.1128/iai.59.3.1153-1161.1991 PMC258381

[282] B. T. Edelson , E. R. Unanue , Immunity 2001, 14, 503. 11371353 10.1016/s1074-7613(01)00139-x

[283] C. M. J. Hu , R. H. Fang , J. Copp , B. T. Luk , L. Zhang , Nat. Nanotechnol. 2013, 8, 336. 23584215 10.1038/nnano.2013.54 PMC3648601

[284] Y. Chen , M. Chen , Y. Zhang , J. H. Lee , T. Escajadillo , H. Gong , R. H. Fang , W. Gao , V. Nizet , L. Zhang , Adv. Healthcare Mater. 2018, 7, 1701366. 10.1002/adhm.201701366 PMC6041168 29436150

[285] B. Mellbye , M. Schuster , mBio 2011, 2, e00131. 21990612 10.1128/mBio.00131-11 PMC3190357

[286] T. N. Ellis , M. J. Kuehn , Microbiol. Mol. Biol. Rev. 2010, 74, 81. 20197500 10.1128/MMBR.00031-09 PMC2832350

[287] J. Holst , D. Martin , R. Arnold , C. C. Huergo , P. Oster , J. O'Hallahan , E. Rosenqvist , Vaccine 2009, 27, B3. 19481313 10.1016/j.vaccine.2009.04.071

[288] J. S. Chahal , T. Fang , A. W. Woodham , O. F. Khan , J. Ling , D. G. Anderson , H. L. Ploegh , Sci. Rep. 2017, 7, 252. 28325910 10.1038/s41598-017-00193-w PMC5427874

[289] C. Hartono , T. Muthukumar , M. Suthanthiran , Cold Spring Harbor Perspect. Med. 2013, 3, a015487. 10.1101/cshperspect.a015487 PMC3753725 24003247

[290] B. N. Cronstein , Pharmacol. Rev. 2005, 57, 163. 15914465 10.1124/pr.57.2.3

[291] E. B. Geer , J. Islam , C. Buettner , Endocrinol. Metab. Clin. North Am. 2014, 43, 75. 24582093 10.1016/j.ecl.2013.10.005 PMC3942672

[292] K. Ghoreschi , P. Thomas , S. Breit , M. Dugas , R. Mailhammer , W. van Eden , R. van der Zee , T. Biedermann , J. Prinz , M. Mack , U. Mrowietz , E. Christophers , D. Schlondorff , G. Plewig , C. A. Sander , M. Rocken , Nat. Med. 2003, 9, 40. 12461524 10.1038/nm804

[293] Z. Zidek , P. Anzenbacher , E. Kmonickova , Br. J. Pharmacol. 2009, 157, 342. 19371342 10.1111/j.1476-5381.2009.00206.x PMC2707982

[294] E. Blanco , A. Hsiao , A. P. Mann , M. G. Landry , F. Meric‐Bernstam , M. Ferrari , Cancer Sci. 2011, 102, 1247. 21447010 10.1111/j.1349-7006.2011.01941.x PMC11158341

[295] T. J. Beldman , M. L. Senders , A. Alaarg , C. Perez‐Medina , J. Tang , Y. Zhao , F. Fay , J. Deichmoller , B. Born , E. Desclos , N. N. van der Wel , R. A. Hoebe , F. Kohen , E. Kartvelishvily , M. Neeman , T. Reiner , C. Calcagno , Z. A. Fayad , M. P. J. de Winther , E. Lutgens , W. J. M. Mulder , E. Kluza , ACS Nano 2017, 11, 5785. 28463501 10.1021/acsnano.7b01385 PMC5492212

[296] T. M. Raimondo , D. J. Mooney , Proc. Natl. Acad. Sci. USA 2018, 115, 10648. 30275293 10.1073/pnas.1806908115 PMC6196479

[297] D. Kwon , B. G. Cha , Y. Cho , J. Min , E. B. Park , S. J. Kang , J. Kim , Nano Lett. 2017, 17, 2747. 28422506 10.1021/acs.nanolett.6b04130

[298] F. Deng , S. He , S. Cui , Y. Shi , Y. Tan , Z. Li , C. Huang , D. Liu , F. Zhi , L. Peng , J. Crohn's Colitis 2019, 13, 482. 30445446 10.1093/ecco-jcc/jjy181

[299] J. Gan , Y. Dou , Y. Li , Z. Wang , L. Wang , S. Liu , Q. Li , H. Yu , C. Liu , C. Han , Z. Huang , J. Zhang , C. Wang , L. Dong , Biomaterials 2018, 178, 95. 29920405 10.1016/j.biomaterials.2018.06.015

[300] H. Kang , H. J. Jung , S. K. Kim , D. S. H. Wong , S. Lin , G. Li , V. P. Dravid , L. Bian , ACS Nano 2018, 12, 5978. 29767957 10.1021/acsnano.8b02226

[301] R. Nosratabadi , M. Rastin , M. Sankian , D. Haghmorad , M. Mahmoudi , Nanomed.: Nanotechnol., Biol. Med. 2016, 12, 1961. 10.1016/j.nano.2016.04.001 27107531

[302] Y. Xiong , W. Gao , F. Xia , Y. Sun , L. Sun , L. Wang , S. Ben , S. E. Turvey , H. Yang , Q. Li , Adv. Healthcare Mater. 2018, 7, 1800510. 10.1002/adhm.201800510 30101578

[303] R. A. Maldonado , R. A. LaMothe , J. D. Ferrari , A. H. Zhang , R. J. Rossi , P. N. Kolte , A. P. Griset , C. O'Neil , D. H. Altreuter , E. Browning , L. Johnston , O. C. Farokhzad , R. Langer , D. W. Scott , U. H. von Andrian , T. K. Kishimoto , Proc. Natl. Acad. Sci. USA 2015, 112, E156. 25548186 10.1073/pnas.1408686111 PMC4299193

[304] R. A. LaMothe , P. N. Kolte , V. Trinh , J. D. Ferrari , T. C. Gelsinger , J. Wong , V. T. Chan , S. Ahmed , A. Srinivasan , P. Deitemeyer , R. A. Maldonado , T. K. Kishimoto , Front. Immunol. 2018, 9, 281. 29552007 10.3389/fimmu.2018.00281 PMC5840162

[305] S. Prasad , T. Neef , D. Xu , J. R. Podojil , D. R. Getts , L. D. Shea , S. D. Miller , J. Autoimmun. 2018, 89, 112. 29258717 10.1016/j.jaut.2017.12.010 PMC5902637

[306] S. Tsai , A. Shameli , J. Yamanouchi , X. Clemente‐Casares , J. Wang , P. Serra , Y. Yang , Z. Medarova , A. Moore , P. Santamaria , Immunity 2010, 32, 568. 20381385 10.1016/j.immuni.2010.03.015

[307] X. Clemente‐Casares , J. Blanco , P. Ambalavanan , J. Yamanouchi , S. Singha , C. Fandos , S. Tsai , J. Wang , N. Garabatos , C. Izquierdo , S. Agrawal , M. B. Keough , V. W. Yong , E. James , A. Moore , Y. Yang , T. Stratmann , P. Serra , P. Santamaria , Nature 2016, 530, 434. 26886799 10.1038/nature16962

[308] M. D. McHugh , J. Park , R. Uhrich , W. Gao , D. A. Horwitz , T. M. Fahmy , Biomaterials 2015, 59, 172. 25974747 10.1016/j.biomaterials.2015.04.003 PMC5997248

[309] T. A. Wynn , A. Chawla , J. W. Pollard , Nature 2013, 496, 445. 23619691 10.1038/nature12034 PMC3725458

[310] Y. Liu , X. Zou , Y. Chai , Y. Yao , Int. J. Biol. Sci. 2014, 10, 520. 24910531 10.7150/ijbs.8879 PMC4046879

[311] J. Han , Y. S. Kim , M. Y. Lim , H. Y. Kim , S. Kong , M. Kang , Y. W. Choo , J. H. Jun , S. Ryu , H. Y. Jeong , J. Park , G. J. Jeong , J. C. Lee , G. H. Eom , Y. Ahn , B. S. Kim , ACS Nano 2018, 12, 1959. 29397689 10.1021/acsnano.7b09107

[312] J. Min , K. Y. Choi , E. C. Dreaden , R. F. Padera , R. D. Braatz , M. Spector , P. T. Hammond , ACS Nano 2016, 10, 4441. 26923427 10.1021/acsnano.6b00087 PMC6501197

[313] K. Masuko , M. Murata , K. Yudoh , T. Kato , H. Nakamura , Int. J. Gen. Med. 2009, 2, 77. 20360891 10.2147/ijgm.s5495 PMC2840553

[314] E. J. Oh , K. Park , K. S. Kim , J. Kim , J. A. Yang , J. H. Kong , M. Y. Lee , A. S. Hoffman , S. K. Hahn , J. Controlled Release 2010, 141, 2. 10.1016/j.jconrel.2009.09.010 19758573

[315] M. Kratz , B. R. Coats , K. B. Hisert , D. Hagman , V. Mutskov , E. Peris , K. Q. Schoenfelt , J. N. Kuzma , I. Larson , P. S. Billing , R. W. Landerholm , M. Crouthamel , D. Gozal , S. Hwang , P. K. Singh , L. Becker , Cell Metab. 2014, 20, 614. 25242226 10.1016/j.cmet.2014.08.010 PMC4192131

[316] M. K. Racke , A. Bonomo , D. E. Scott , B. Cannella , A. Levine , C. S. Raine , E. M. Shevach , M. Rocken , J. Exp. Med. 1994, 180, 1961. 7525845 10.1084/jem.180.5.1961 PMC2191757

[317] K. Krishnan , B. Arnone , A. Buchman , Inflammatory Bowel Dis. 2011, 17, 410. 10.1002/ibd.21316 20848489

[318] G. Curtale , M. Mirolo , T. A. Renzi , M. Rossato , F. Bazzoni , M. Locati , Proc. Natl. Acad. Sci. USA 2013, 110, 11499. 23798430 10.1073/pnas.1219852110 PMC3710884

[319] L. Peng , H. Zhang , Y. Hao , F. Xu , J. Yang , R. Zhang , G. Lu , Z. Zheng , M. Cui , C. Qi , C. Chen , J. Wang , Y. Hu , D. Wang , S. Pierce , L. Li , H. Xiong , EBioMedicine 2016, 14, 83. 27825654 10.1016/j.ebiom.2016.10.041 PMC5161420

[320] T. T. Lee , J. R. García , J. I. Paez , A. Singh , E. A. Phelps , S. Weis , Z. Shafiq , A. Shekaran , A. Del Campo , A. J. García , Nat. Mater. 2015, 14, 352. 25502097 10.1038/nmat4157 PMC4336636

[321] S. Sakaguchi , T. Yamaguchi , T. Nomura , M. Ono , Cell 2008, 133, 775. 18510923 10.1016/j.cell.2008.05.009

[322] M. Romano , S. L. Tung , L. A. Smyth , G. Lombardi , Transplant Int. 2017, 30, 745. 10.1111/tri.12909 28012226

[323] A. Chaudhry , R. M. Samstein , P. Treuting , Y. Liang , M. C. Pils , J. M. Heinrich , R. S. Jack , F. T. Wunderlich , J. C. Bruening , W. Mueller , A. Y. Rudensky , Immunity 2011, 34, 566. 21511185 10.1016/j.immuni.2011.03.018 PMC3088485

[324] S. Sakaguchi , N. Sakaguchi , M. Asano , M. Itoh , M. Toda , J. Immunol. 1995, 155, 1151. 7636184

[325] P. M. Tiwari , K. Vig , V. A. Dennis , S. R. Singh , Nanomaterials 2011, 1, 31. 28348279 10.3390/nano1010031 PMC5315048

[326] M. A. Kovach , T. J. Standiford , Int. Immunopharmacol. 2011, 11, 1399. 21624505 10.1016/j.intimp.2011.05.013 PMC3575025

[327] J. Carbone , N. Del Pozo , A. Gallego , E. Sarmiento , Expert Rev. Anti‐Infect. Ther. 2011, 9, 405. 21504398 10.1586/eri.10.178

[328] D. S. Riminton , H. P. Hartung , S. W. Reddel , Curr. Opin. Neurol. 2011, 24, 217. 21519254 10.1097/WCO.0b013e328346d47d

[329] C. Keijzer , R. van der Zee , W. van Eden , F. Broere , Front. Immunol. 2013, 4, 245. 23970886 10.3389/fimmu.2013.00245 PMC3747555

[330] B. Bisikirska , J. Colgan , J. Luban , J. A. Bluestone , K. C. Herold , J. Clin. Invest. 2005, 115, 2904. 16167085 10.1172/JCI23961 PMC1201661

[331] A. B. Cosimi , R. B. Colvin , R. C. Burton , R. H. Rubin , G. Goldstein , P. C. Kung , W. P. Hansen , F. L. Delmonico , P. S. Russell , N. Engl. J. Med. 1981, 305, 308. 6454075 10.1056/NEJM198108063050603

[332] S. Miller , R. P. Wetzig , H. N. Claman , J. Exp. Med. 1979, 149, 758. 85683 10.1084/jem.149.3.758 PMC2184831

[333] A. McLarnon , Nat. Rev. Gastroenterol. Hepatol. 2012, 9, 559. 10.1038/nrgastro.2012.167 22926154

[334] P. Desreumaux , A. Foussat , M. Allez , L. Beaugerie , X. Hebuterne , Y. Bouhnik , M. Nachury , V. Brun , H. Bastian , N. Belmonte , M. Ticchioni , A. Duchange , P. Morel‐Mandrino , V. Neveu , N. Clerget‐Chossat , M. Forte , J. F. Colombel , Gastroenterology 2012, 143, 1207. 22885333 10.1053/j.gastro.2012.07.116

[335] S. Singha , K. Shao , Y. Yang , X. Clemente‐Casares , P. Sole , A. Clemente , J. Blanco , Q. Dai , F. Song , S. W. Liu , J. Yamanouchi , C. S. Umeshappa , R. H. Nanjundappa , P. Detampel , M. Amrein , C. Fandos , R. Tanguay , S. Newbigging , P. Serra , A. Khadra , W. C. W. Chan , P. Santamaria , Nat. Nanotechnol. 2017, 12, 701. 28436959 10.1038/nnano.2017.56

[336] E. R. Steenblock , T. Fadel , M. Labowsky , J. S. Pober , T. M. Fahmy , J. Biol. Chem. 2011, 286, 34883. 21849500 10.1074/jbc.M111.276329 PMC3186438

[337] K. Nakamura , A. Kitani , W. Strober , J. Exp. Med. 2001, 194, 629. 11535631 10.1084/jem.194.5.629 PMC2195935

[338] A. Matsiko , Nat. Mater. 2018, 17, 472. 10.1038/s41563-018-0091-8 29795216

[339] L. M. Kranz , M. Diken , H. Haas , S. Kreiter , C. Loquai , K. C. Reuter , M. Meng , D. Fritz , F. Vascotto , H. Hefesha , C. Grunwitz , M. Vormehr , Y. Huesemann , A. Selmi , A. N. Kuhn , J. Buck , E. Derhovanessian , R. Rae , S. Attig , J. Diekmann , R. A. Jabulowsky , S. Heesch , J. Hassel , P. Langguth , S. Grabbe , C. Huber , O. Tuereci , U. Sahin , Nature 2016, 534, 396. 27281205 10.1038/nature18300

[340] W. Song , M. Das , Y. Xu , X. Si , Y. Zhang , Z. Tang , X. Chen , Mater. Today Nano 2019, 5, 100029.

[341] R. Y. Pelgrift , A. J. Friedman , Adv. Drug Delivery Rev. 2013, 65, 1803. 10.1016/j.addr.2013.07.011 23892192

[342] N. Beyth , Y. Houri‐Haddad , A. Domb , W. Khan , R. Hazan , J. Evidence‐Based Complementary Altern. Med. 2015, 2015, 246012. 10.1155/2015/246012 PMC4378595 25861355

[343] D. J. Chen , N. Osterrieder , S. M. Metzger , E. Buckles , A. M. Doody , M. P. DeLisa , D. Putnam , Proc. Natl. Acad. Sci. USA 2010, 107, 3099. 20133740 10.1073/pnas.0805532107 PMC2840271

[344] V. Gujrati , S. Kim , S. H. Kim , J. J. Min , H. E. Choy , S. C. Kim , S. Jon , ACS Nano 2014, 8, 1525. 24410085 10.1021/nn405724x

[345] E. A. Scott , A. Stano , M. Gillard , A. C. Maio‐Liu , M. A. Swartz , J. A. Hubbell , Biomaterials 2012, 33, 6211. 22658634 10.1016/j.biomaterials.2012.04.060

[346] T. Endoh , T. Ohtsuki , Adv. Drug Delivery Rev. 2009, 61, 704. 10.1016/j.addr.2009.04.005 19383521

[347] S. B. Fonseca , M. P. Pereira , S. O. Kelley , Adv. Drug Delivery Rev. 2009, 61, 953. 10.1016/j.addr.2009.06.001 19538995

[348] S. M. Moghimi , H. M. Patel , Adv. Drug Delivery Rev. 1998, 32, 45. 10.1016/s0169-409x(97)00131-2 10837635

[349] E. Blanco , H. Shen , M. Ferrari , Nat. Biotechnol. 2015, 33, 941. 26348965 10.1038/nbt.3330 PMC4978509

[350] Z. Liu , W. Jiang , J. Nam , J. J. Moon , B. Y. S. Kim , Nano Lett. 2018, 18, 6655. 30185039 10.1021/acs.nanolett.8b02340 PMC6238186

[351] W. Jiang , C. A. von Roemeling , Y. Chen , Y. Qie , X. Liu , J. Chen , B. Y. S. Kim , Nat. Biomed. Eng. 2017, 1, UNSP 0029.

[352] B. Yu , Y. Mao , Y. Yuan , C. Yue , X. Wang , X. Mo , D. Jarjoura , M. E. Paulaitis , R. J. Lee , J. C. Byrd , L. J. Lee , N. Muthusamy , Biomaterials 2013, 34, 6185. 23726226 10.1016/j.biomaterials.2013.04.063 PMC3756150

[353] D. J. Irvine , M. A. Swartz , G. L. Szeto , Nat. Mater. 2013, 12, 978. 24150416 10.1038/nmat3775 PMC3928825

[354] J. C. Sunshine , K. Perica , J. P. Schneck , J. J. Green , Biomaterials 2014, 35, 269. 24099710 10.1016/j.biomaterials.2013.09.050 PMC3902087

